# A Computational Framework Discovers New Copy Number Variants with Functional Importance

**DOI:** 10.1371/journal.pone.0017539

**Published:** 2011-03-29

**Authors:** Samprit Banerjee, Derek Oldridge, Maria Poptsova, Wasay M. Hussain, Dimple Chakravarty, Francesca Demichelis

**Affiliations:** 1 Department of Public Health, Weill Cornell Medical College, New York, New York, United States of America; 2 Department of Pathology and Laboratory Medicine, Weill Cornell Medical College, New York, New York, United States of America; 3 Institute for Computational Biomedicine, Weill Cornell Medical College, New York, New York, United States of America; Kyushu Institute of Technology, Japan

## Abstract

Structural variants which cause changes in copy numbers constitute an important component of genomic variability. They account for 0.7% of genomic differences in two individual genomes, of which copy number variants (CNVs) are the largest component. A recent population-based CNV study revealed the need of better characterization of CNVs, especially the small ones (<500 bp).We propose a three step computational framework (Identification of germline Changes in Copy Number or IgC2N) to discover and genotype germline CNVs. First, we detect candidate CNV loci by combining information across multiple samples without imposing restrictions to the number of coverage markers or to the variant size. Secondly, we fine tune the detection of rare variants and infer the putative copy number classes for each locus. Last, for each variant we combine the relative distance between consecutive copy number classes with genetic information in a novel attempt to estimate the reference model bias. This computational approach is applied to genome-wide data from 1250 HapMap individuals. Novel variants were discovered and characterized in terms of size, minor allele frequency, type of polymorphism (gains, losses or both), and mechanism of formation. Using data generated for a subset of individuals by a 42 million marker platform, we validated the majority of the variants with the highest validation rate (66.7%) was for variants of size larger than 1 kb. Finally, we queried transcriptomic data from 129 individuals determined by RNA-sequencing as further validation and to assess the functional role of the new variants. We investigated the possible enrichment for variant's regulatory effect and found that smaller variants (<1 Kb) are more likely to regulate gene transcript than larger variants (p-value = 2.04e-08). Our results support the validity of the computational framework to detect novel variants relevant to disease susceptibility studies and provide evidence of the importance of genetic variants in regulatory network studies.

## Introduction

The extent to which genetic differences among humans are associated with human disease susceptibility is still unknown [1]. Genetic differences, sometimes referred to as genomic variability, can be of several types, including single nucleotide polymorphism (SNPs) and structural variation mainly consisting of DNA copy number changes. Copy number variants (CNV) are defined as intra- or inter- chromosomal duplications or deletions of 1 Kb or larger DNA segments, which vary in copy number among individuals. The definition of CNV is elusive due to the existence of long interspersed nucleotide elements (LINEs) and small (≤1 Kb) insertions and deletions [2].

CNVs are currently estimated to encompass between 6% and 10% of the human reference genome assembly (Database of Genomic Variants (DGV), http://projects.tcag.ca/variation/) [3], [4]. The popular belief used to be that SNPs represent the vast majority (about 0.1% of the total nucleotide content of the genome) of genomic differences in humans. However, recent studies have shown that structural variation can account for variability in as much as 0.7% of the total nucleotide content, of which CNVs are the largest component [4]. Unlike the catalogue of known SNPs, the number and characterization of CNVs in humans remain incomplete. Earlier this year, Conrad et al 2010 [5] presented the most comprehensive population-based CNV map where they have discovered 80–90% of common CNVs (Minor Allele Frequency (MAF)>5%) greater than 1 kb in length and have been able to genotype approximately 40% of these. Nonetheless it is believed that CNVs, especially smaller ones, and INDELs are underrepresented in existing databases and require better characterization [5].

Genomic variants may play an important functional role in the human transcriptome regulation in both normal and disease states. Emerging data suggest that regulatory complexity of the human transcriptome can partially be explained by genetic make-up. Two recent papers from Montgomery et al 2010 [6] and Pickrell et al 2010 [7] evaluated the regulatory effects of SNPs by performing extensive expression Quantitative Trait Locus (eQTL) analysis in Utah residents with Northern and Western European ancestry (CEU) and Yoruba from Ibadan, Nigeria (YRI) populations. They confirmed that the effect of SNPs on gene regulation can be local (i.e., by the disruption or duplication of coding sequence) or long-range and can translate into direct effect (e.g., dosage) or inverse effect based upon the involvement of enhancers or repressors. They demonstrate that SNPs have an effect on protein coding and non-coding genes, on isoform diversity and on transcript diversity, which can translate into protein structure diversity [6], [7]. In 2007 Stranger et al [8] showed that CNVs, in addition to SNPs, have a significant impact on gene expression phenotypes and reported that the signal from the two types of variation had little overlap. These observations, taken together, attest the importance of structural variations as critical to understanding human protein regulation in the normal and disease states.

**Figure 1:**
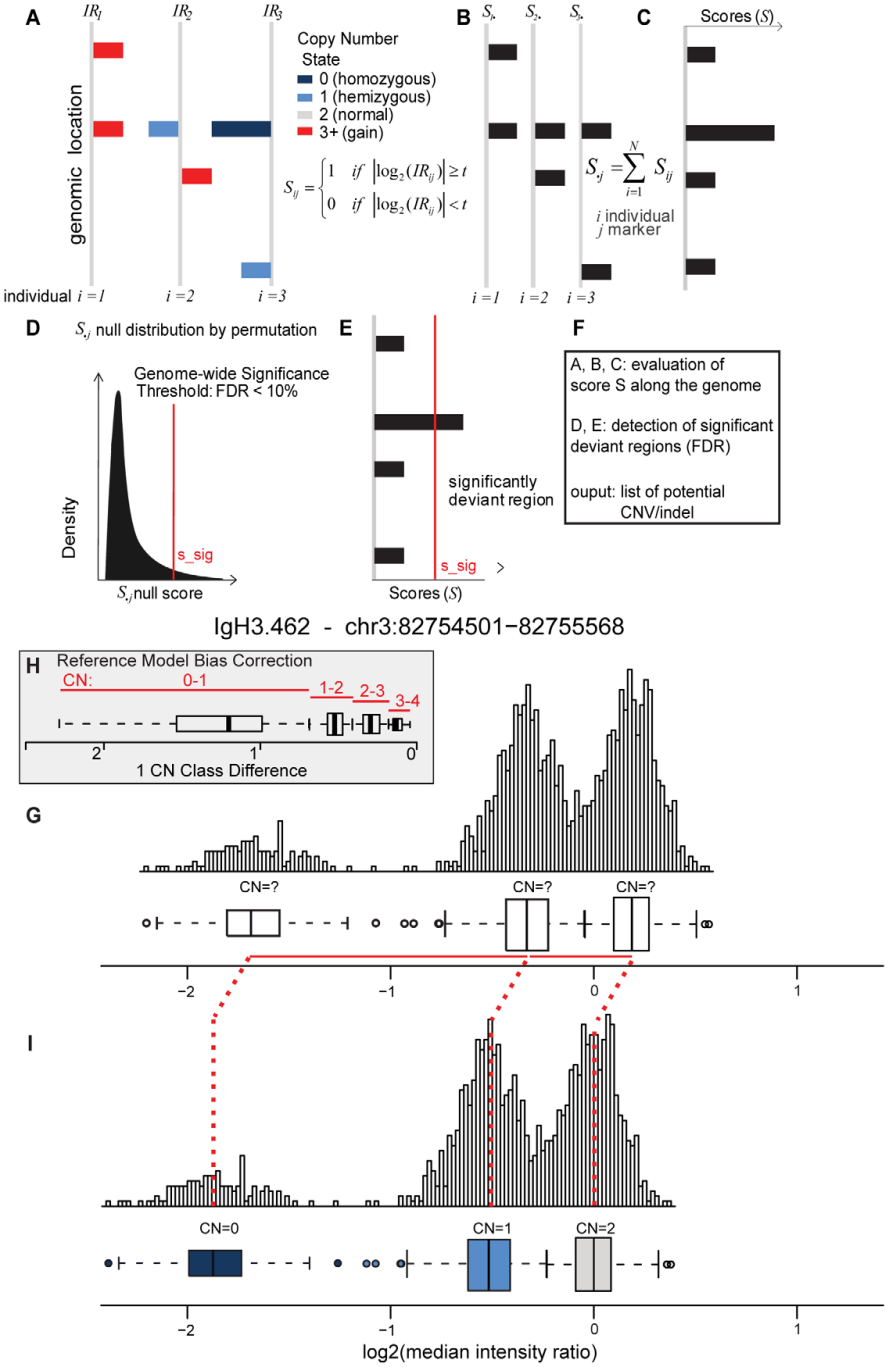
Schematic of the approach used for the Identification of germline Changes in Copy Numbers (IgC2N). IgC2N is a multistep approach, which includes the identification of potential CNV loci along the genome (A–F), a bias correction step (H) and the CN genotyping (I), leveraging the experimental data from many samples. (A–I) The log2 intensity ratio signals or the segmented signal (A) is dichotomized on a marker and sample basis (B). A genome-wide score vector **S** is obtained by summing the transformed signal across all samples on a marker basis (C). The null distribution of the score is obtained by permutations in order to identify the level of significant deviation of the score **S**, S_sig, from the baseline signal. (D) S_sig value corresponding to a pre-specified FDR threshold is applied to the data vector **S** (E). The intermediate output is a collection of putative polymorphic loci across the genome. No restriction on size or coverage is applied (F). A Gaussian Mixture Model (GMM) is applied to predict the CN classes (genotypes not assigned) (G). The distance between the median of consecutive CN classes (1 CN class difference) is compared to the 1 CN class difference of all CNVs, and relative classes are inferred (H). Along with 1 CN class differences, the presence of “0” class and expected direction of bias are also considered to infer the genotypes of these CN classes and the reference model bias is estimated (I).

In this paper, we propose a 3-step method for the Identification of germline Changes in Copy Number, IgC2N, to discover and genotype CNVs and show its application to genome-wide array-based data (**Figure 1**).

There is a large body of literature describing methods to the problem of estimating DNA copy numbers across the genome. Among these, PennCNV [9] and Birdsuite [10] packages are the most comprehensive software tools providing copy number calls for Illumina SNP bead arrays and Affymetrix Genome Wide 6.0 SNP arrays, respectively. PennCNV uses a Hidden Markov Model (HMM) based approach on SNP genotyping data to detect CNV on a sample basis. On the other hand, Birdsuite packages sequentially assigns copy number genotypes across common CNVs (Canary: uses a Gaussian Mixture Model approach and heuristics to determine the CN genotypes), calls genotypes of SNPs (Birdseed), identifies rare CNVs via a hidden Markov model (HMM) approach (Birdseye) on a sample basis and generates an integrated sequence and CN genotype at each locus (Fawkes). Other computational tools to detect CNVs exist [11], [12], [13], [14]. While these approaches determine copy number on an individual sample basis, we propose a method, which borrows information across multiple samples to propose candidate CNV loci, gaining power as the sample size increases. We use smoothed intensity data from multiple samples to distinguish meaningful copy number change signal from random background signal, an approach similar in spirit to the one described in Beroukhim et al [15].

The common practice to eliminate variation due to marker-specific hybridization is to normalize signal intensities with respect to a reference model on a marker basis. The reference model could be a single sample or an average across several samples. Although this successfully eliminates the technical variation, it introduces a bias as the inferred copy number state would be only with respect to the reference model [16]. Given the goal of accounting for this bias and inferring CNV genotypes, we propose steps two and three of our method. In step two, we fit Gaussian Mixture Models using EM-algorithm [17] as implemented in R/CNVtools [18] to each candidate CNV and record the relative distance between consecutive copy number classes. Using this information along with biological information we then estimate the location bias introduced by the reference model or the reference model bias and infer copy number genotypes.

There are three distinct advantages in our approach. First, we use information across multiple samples to discover CNVs which improves power to detect rare CNVs. This aspect will be demonstrated by an extensive simulation study and by the analysis of the HapMap data. Second, we explicitly estimate the reference model bias, which to our knowledge is a novel attempt. Also, previous studies have focused on CNV discovery, with at least one notable exception [19], and not on genotyping owing to technical challenges and lack of a comprehensive computational framework. Genotyping CNVs is extremely important to understand the dosage effect of genes in the context of human diseases. Third, the detection method does not pose any restriction to either the number of markers to define a variant or to the size of the variant.

We apply IgC2N to the genomic profiles of 1250 HapMap individuals. We compare the performance of our method (in terms of CNV detection) on a subset of individuals previously analyzed using Birdsuite [19]. To independently validate the newly detected variants, we query 42 million marker tiling array data [5]. We then assess the functional impact of the variants by analyzing the mRNA levels of 129 HapMap individuals using next generation sequencing data from Pickrell et al [7] and Montgomery et al [6].

Unless explicitly stated we will refer to all variants as CNVs. Throughout the manuscript we will refer variants of size ≤1 kb as short CNVs (sCNVs).

## Results

### Simulation Study

**Figure 2:**
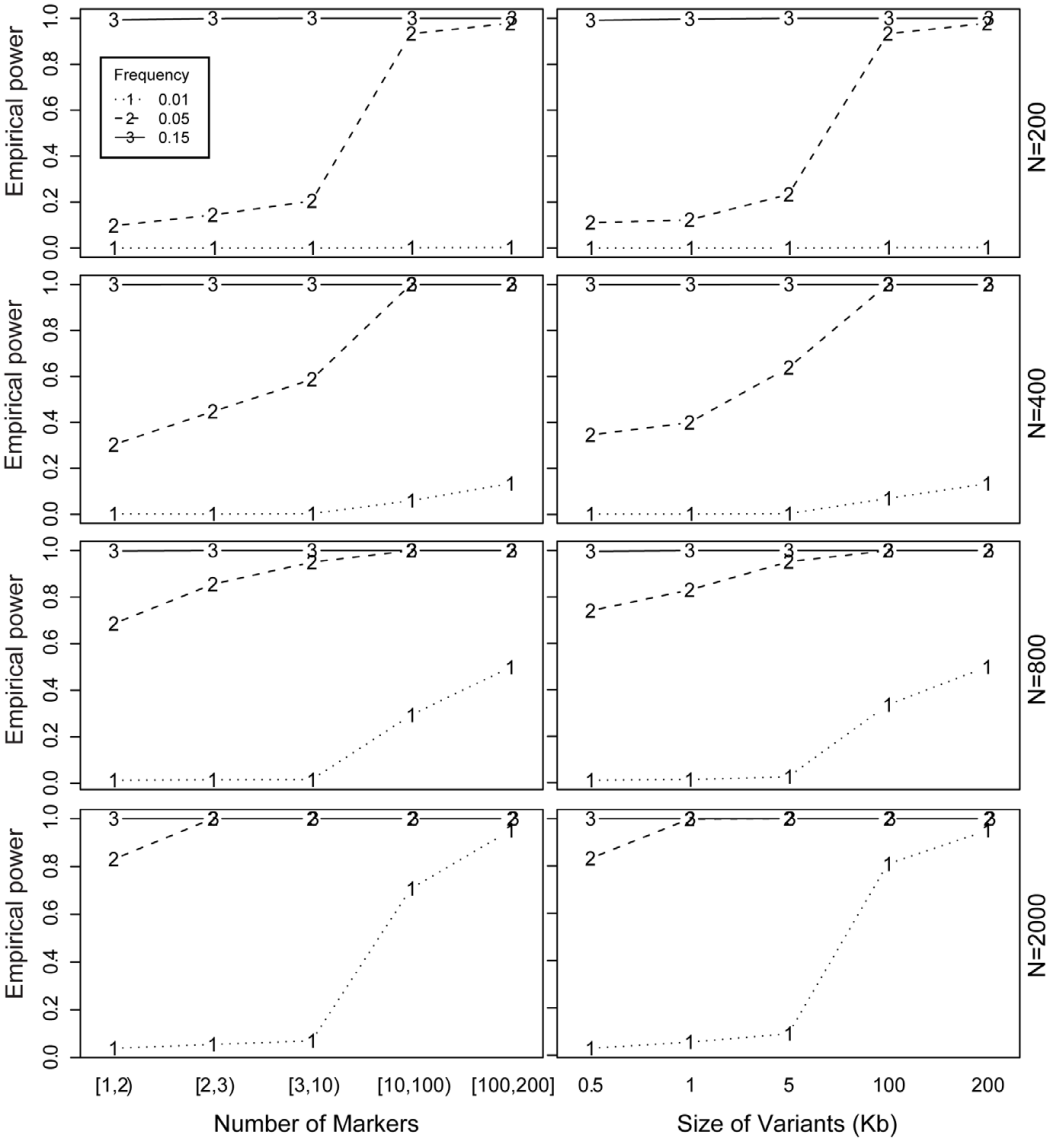
Results of Power Simulation Study as function of Size and Coverage. *In silico* power computations for IgC2N. The panel of 8 plots is organized in rows by sample size of the datasets used for simulations and in columns by the number of markers covering a CNV and size (in kb) of a CNV. Each plot shows the average power to detect CNVs with three different frequencies, i.e. 1%, 5% and 15% for the dotted, dashed and solid lines respectively.

Firstly, we evaluated the predictive performance of IgC2N on simulated data. We simulated datasets with several CNVs spanning a wide range of characteristics in terms of size, incidence (frequency in the population) and type of variant, namely deletion, gain or deletion/gain. To assess the adequate number of datasets to be used in the simulation, we monitored the behavior and stabilization of the false positive rate over simulated datasets (**File S1**) and power (data not shown) by generating up to 500 datasets with a sample size of 200 and found that both stabilize at 100 datasets. **Figure 2** shows the average empirical power (described in details in the Materials and Methods section) for each CNV characteristic (e.g. size and frequency). Note that the size of a CNV is inherently related to the number of markers covering the CNV which depends on the marker distribution of the specific platform in question. **Figure 2** indicates that IgC2N has at least 80% power to detect polymorphisms of frequency 5% in datasets of sample size 200 which are at least 100 kb long or have at least 10 covering markers. On the other hand, with larger sample size (2000 or more) IgC2N has 80% or more power to detect similar CNVs (in terms of size of the CNV and number of covering markers) with frequency of 1% or more. The False Positive Rate (FPR) for detecting CNVs is provided in greater details in **File S1**. Briefly, the mean (maximum) FPR (over 100 datasets) was 0.0072 (0.04), 0.0026 (0.01), 0.0029 (0.01) and 0.0113 (0.03) for sample sizes of 200, 400, 800 and 2000 respectively.

### IgC2N applied to HapMap individuals

The IgC2N pipeline was run on the HapMap phase 3 dataset (1250 samples profiled on Affymetrix 6.0 platform). IgC2N identified 2497 variants including 734 variants which were not reported before (up to the DGV March 2010 release). These 734 CNVs are termed as *novel CNVs*. Before moving to their characterization, we focused on comparing the performance of IgC2N with other competing methods and previously reported CNVs. For direct comparison, we focused on the data reported by McCarroll et al [19] on HapMap phase 2 individuals profiled with the same platform (Affymetrix 6.0). The data were analyzed using the Birdsuite software and 1319 CNVs were reported. Our simulation study indicates that IgC2N has 80% power to detect a CNV with frequency of polymorphism ≥5% with a sample size of 200. We impose this restriction on the set of McCarroll CNVs, i.e we consider McCarroll CNVs for which at least 5% of the samples have CN states other than “2”. We call two CNVs equal if a target CNV overlaps with at least 50% of the reference CNV (McCarroll CNV) in terms of base pairs. IgC2N (on HapMap phase 2) detects 397/457 or 86.87% of McCarroll CNVs while McCarroll detects 602/2070 or 29.08% of IgC2N CNVs. This provides a comparison with a gold standard (Birdsuite) and demonstrates that IgC2N is able to detect majority of the CNVs detected by Birdsuite. 1468 of the 2070 CNVs detected by IgC2N were not reported by McCarroll et al 2008 [19] and 852 (58.04%) of these CNVs were reported in DGV by other investigators indicating the credibility of the IgC2N CNVs. However, for CNVs with polymorphism frequency <5% the overlap between IgC2N and McCarroll is 35.57%. The low overlap is due to the fact that Birdseye detects CNVs on a sample basis and while IgC2N uses multiple samples and therefore is underpowered to detect less frequent CNVs. We evaluated the concordance of CN genotypes (at the level of gains, losses and CN 2) for every HapMap individual for the 397 McCarroll CNVs detected by IgC2N and found that majority of the CNVs showed high degree of concordance (between 90–100%) across samples. Please refer to **File S1** for the histogram of the concordance of CN genotypes.

### HapMap trio discordance

The mother-father-child trios of the HapMap phase 3 data were used to evaluate Mendelian consistency of the data. A CNV is called discordant in a trio if the child of the trio has a polymorphism and neither parent have it. Unless the CNV is *de novo*, a discordant result is indicative of a false positive call in the child or a false negative call in either of the parent. For every CNV the discordant rate (percentage of discordant trios) was evaluated. The average discordant rate for the IgC2N detected CNVs is 30.31% (29.34–31.29% 95% CI) while that for the set of novel CNVs is 27.81% (26.21–29.41% 95% CI). When considering CNVs with at least 1% frequency of polymorphism, the discordant rates reduced to 24.21% (23.41–25.01% 95 CI) and 25.13% (23.02–27.24% 95% CI). The Mendelian discordant rates stratified by CNV frequencies are graphically presented in **File S1**. These discordant rates are significantly lower than that of Birdseye and that expected by chance as presented in [11] and [20] while being comparable with ÇOKGEN [11]. Considering the discordant rate on a trio basis, the average percentage of discordant CNVs for each trio was 18.49% (17.7%–19.28%, 95% CI). These percentages constitute a small fraction of *de novo* CNVs [21] and false positives/false negatives.

### Characterization of the novel CNV

The characterization of 2497 detected variants is summarized in **File S1**. Here we focus on the characterization of the novel CNVs detected by IgC2N. **Table 1** and **Table 2** describe the frequency and the size of the novel CNVs against different types of polymorphisms. Each CNV is classified into *del* (deletion only: where the polymorphisms are deletions only), *gain* (gains only: where the polymorphisms are gains only), *del-gain* (deletion and gain: where the polymorphism are both deletions and gains) only if the overall polymorphic incidence of the variant is more than 5%. The majority (61.48%) of novel *dels* have Minor Allele Frequency (or MAF which is the frequency of the CN state different from the major class) ≤5%, while majority of gains have MAF between 10–30% (**Table 1**). **Table 2** presents the tabulation of size (in Kilobases) of the variant across different types of polymorphisms. Among all the novel variants, the majority (64.31%) are sCNVs (<1 kb in size). Among the novel CNVs, the majority are small in size (1–10 kb) and this pattern is consistent across different types of polymorphisms.

**Table 1:** Frequency of Copy Number Variants.

MAF	(0,0.05]	(0.05,0.1]	(0.1,0.3]	(0.3,0.5]	Total
**All**	417 (56.81%)	88 (11.99%)	174 (23.71%)	55 (7.49%)	734 (100%)
**Del**	249 (61.48%)	59 (14.57%)	73 (18.02%)	24 (5.93%)	405 (100%)
**Gains**	50 (29.76%)	6 (3.57%)	81 (48.21%)	31 (18.45%)	168 (100%)
**Del/Gains**	47 (52.22%)	23 (25.56%)	20 (22.22%)	0 (0%)	90 (100%)
**≤5%**	71 (100%)	0 (0%)	0 (0%)	0 (0%)	71 (100%)

**Table 2:** Size of sCNVs and Copy Number Variants in kb.

	sCNVs	CNV		
Size in kb	(0,1]	(1,10]	(10,100]	Total
**All**	472 (64.31%)	233 (31.74%)	29 (3.95%)	734 (100%)
**Del**	277 (68.4%)	121 (29.88%)	7 (1.73%)	405 (100%)
**Gains**	87 (51.79%)	64 (38.1%)	17 (10.12%)	168 (100%)
**Del/Gains**	62 (68.89%)	27 (30%)	1 (1.11%)	90 (100%)
**≤5%**	46 (64.79%)	21 (29.58%)	4 (5.63%)	71 (100%)

In agreement with previous studies [22], gene and exon overlap analysis showed that 40–48% of CNVs overlap genes for each CNV size range (**Table 3**), while the percentage of variants overlapping exons is overall lower and increases with larger CNVs. Thus the proportion of variants which overlap exons is 2.97% for CNVs smaller than 1 kb, 8.58% for CNVs ranging from 1 Kb to 10 Kb in size, and 17.24% for CNVs larger than 10 kb.

**Table 3:** Gene or Exon overlap with sCNVs or Copy Number Variants.

	sCNVs	CNV		
Size in kb	(0,1]	(1,10]	(10,100]	Total
**Gene Overlap**	189 (40.04%)	95 (40.77%)	14 (48.28%)	298 (40.6%)
**Exon Overlap**	14 (2.97%)	20 (8.58%)	5 (17.24%)	39 (5.31%)
**All**	472 (100%)	233 (100%)	29 (100%)	734 (100%)

We then analyzed the sequences around the putative breakpoints to assess the distribution of the CNV formation mechanisms (**Table 4**). In accordance with previous studies [22] Non Homologous Recombination (NHR) constitutes the major part of all CNV formation mechanisms (69.21%), while Transposable Element Insertion (TEI) and Variable Number of Tandem Repeat (VNTR) take up 20.84% and 6.27%, respectively. Non-allelic homologous recombination (NAHR) events constitute 3.68% of all CNV mechanisms. VNTR events are most likely underrepresented in most of the studies due to the difficulty in querying those sequences. In our set of novel CNVs the major contributors to the VNTR class (9.32%) are sCNVs (size <1 kb) [23].

**Table 4:** Mechanism of formation of sCNVs or Copy Number Variants.

	sCNVs	CNV		
Size in kb	(0,1]	(1,10]	(10,100]	Total
**NAHR**	0 (0%)	24 (10.3%)	3 (10.34%)	27 (3.68%)
**NHR**	327 (69.28%)	157 (67.38%)	24 (82.76%)	508 (69.21%)
**TEI**	101 (21.4%)	50 (21.46%)	2 (6.9%)	153 (20.84%)
**VNTR**	44 (9.32%)	2 (0.86%)	0 (0%)	46 (6.27%)
**All**	472 (100%)	233 (100%)	29 (100%)	734 (100%)

### Validation of the novel (IgC2N discovered) CNVs

**Figure 3:**
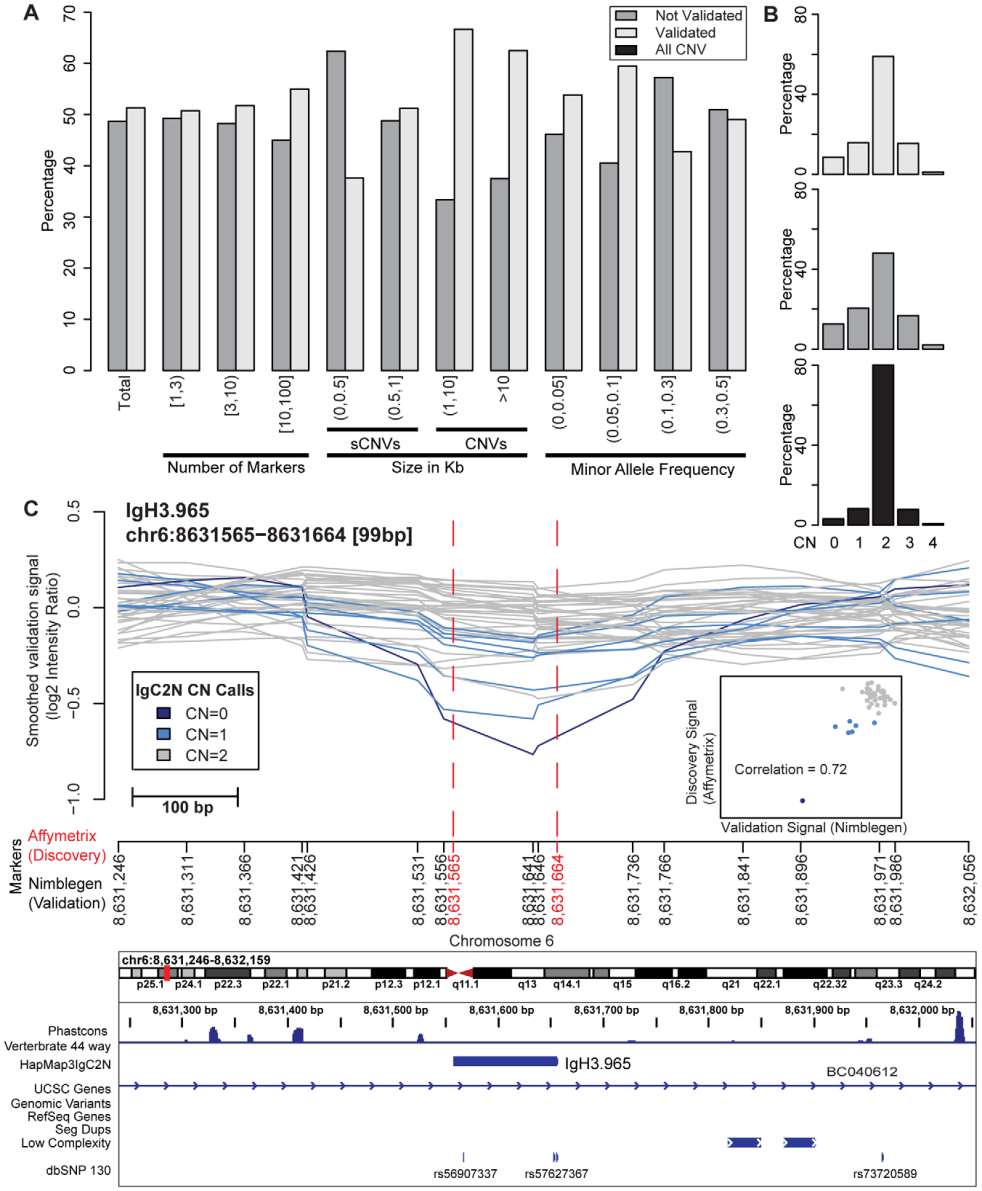
Validation study summary. (A) Barplots of the rate (percentage) of validation categorized with respect to number of marker coverage, size of the variant and its minor allele frequency. (B) The frequency distribution of CN genotypes of validated, not validated and all CNV. (C) An example of a new variant validated by NimbleGen data: The line plots of smoothed intensity signal using 42M NimbleGen platform for each of 40 HapMap samples showing polymorphism for the locus IgH3.965 on Chromosome 6. A scatter plot (inset) of the discovery signal (x-axis) and the validation signal (y-axis) color coded with respect to the IgC2N CN call on the discovery samples.

We assessed the detection power and the performance of IgC2N with respect to McCarroll et al [19]. To further validate our approach and to explicitly assess credibility of the novel 734 CNVs, we utilized the recent data from the Genome Structural Variation (GSV) Consortium (ftp://ftp.sanger.ac.uk/pub4/humgen/cnv/), where 40 HapMap individuals were profiled using a set of tiling arrays with approximately 75 bp resolution. Note that none of the 734 novel CNVs was reported by the original study's analysis of this dataset [5]. For each predicted novel CNV, we considered NimbleGen data within the genomic location and evaluated their correlations with the Affymetrix data used for IgC2N detection on a sample basis. This approach takes into consideration the limited ability of Affymetrix platform to precisely define variant breakpoints and the difference between the discovery and the validation platform probe length (25-mers versus ∼60). We investigated the validation performance on the basis of the discovery platform marker coverage, the predicted variant size and the allele frequency as evaluated in the discovery dataset. The results showed that the best validation rate occurs for CNVs (variants of more than 1 kb in size), with no advantage for very large variants and that neither higher marker coverage nor higher MAF play a role (**Figure 3A**). 66.26% of novel CNVs were validated, whereas the rate for very small ones (≤500 bp) was as low as 37.6%. The genotype distributions of the validated and non validated variants are similar (see **Figure 3B**). An example of a very small variant (∼100 bp) discovered by IgC2N and validated using the independent platform is presented in **Figure 3C**. The smoothed signals from validation platform along the predicted genomic location are plotted for the 40 samples and color coded based on the predicted (IgC2N) CN genotype. The variant resides within an intron of the BC040612 gene. All Repeat Mask sub-tracks other than Low Complexity are omitted as empty.

### Regulatory effect of CNVs and short CNVs

We posited that functionally significant CNVs should contribute to gene regulation. To this end, we explored for association between copy number states and gene transcripts in *cis*. We interrogated the mRNA expression profiles from lymphoblastoid cell lines of 129 HapMap individuals [24] from CEU [6] (N = 60) and YRI [7] (N = 69) populations for association with copy number changes for all the detected variants applying a 2 Mb window. We detected previously reported and unreported CNV-gene expression associations in both populations such as UGT2B17, SIGLEC14, GSTM1 (previously reported [8]) and GSTT1, OR7D2, SIGLEC14, RHD, and IFI27L1 (unreported, to the best of our knowledge) (graphical representation in **File S1**) and a larger number of population specific associations (tabulated data are presented in **File S1**). Overall, larger number of significant associations was detected in the CEU population, possibly due to the higher coverage. To evaluate the extent of novel information with respect to the genetic contribution to the transcriptome, we considered association results both from SNP-gene and clone-gene analysis from Stranger et al [8] and identified ∼250 novel associations (at 10% FDR, tabulated data is presented in **File S1**). Among the strongest associations, we observed outlier expression levels for individuals harboring one or more copy number gains at loci of rare polymorphism (**File S1**). Fifty-seven (out of 298) of the non-validated CNVs based on NimbleGen data showed a statistically significant effect on gene transcript.

**Figure 4:**
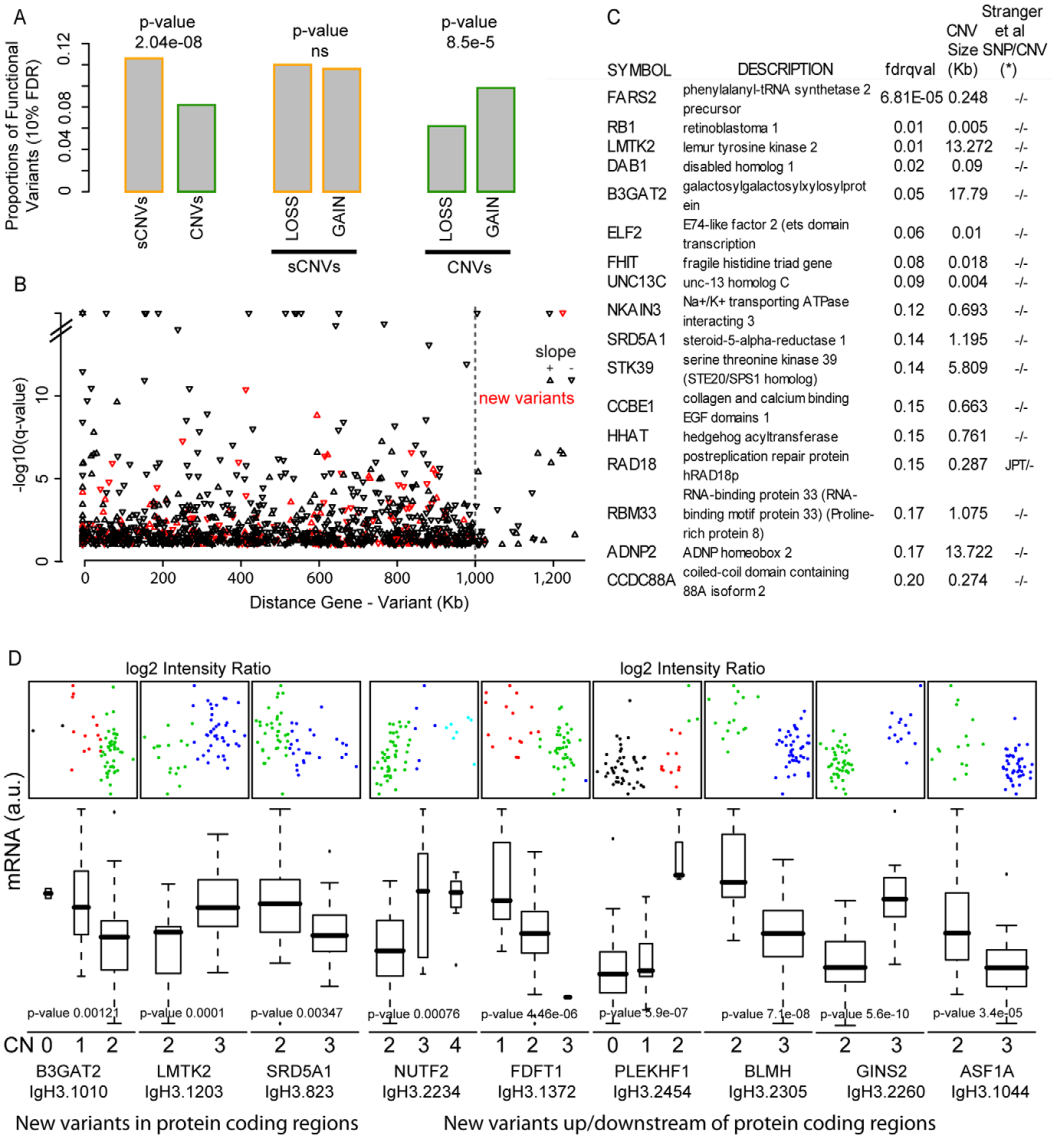
Functional impact of CNVs on human transcriptome. (A) Proportion of functional variants with respect to variant size and type of polymorphisms. Percentages are evaluated with respect to each subclass. (B) Significance of associations with respect to gene-variant distance. The *cis* analysis included 2 Mb windows. Minus log 10 of the q-values are plotted against the distance between the mid points of genes and variants. Up and down arrows depict the direction of the association. Red symbols identify data points corresponding to the new CNVs. (C) List of top ranked associations involving new variant residing within protein coding regions. (D) Examples of new variants showing significant effect on gene transcript. mRNA levels are plotted against the copy number states of new variants identified by IgC2N (box plots) and against the copy number intensity ratios (scatter plots). P-values from the regression analysis against copy number states are reported.

Focusing on CEU population datasets, we then investigated possible enrichment for CNVs regulatory effect with respect to MAF, genomic complexity (segmental duplication), mechanism of formation, variant size and type of polymorphisms within the *cis* analysis window. Small variants are more likely to regulate gene transcript than larger variants (p = 2.04e-08) with no preference in terms of type of polymorphism. Whereas, within variants of size >1 kb, variants involving copy number gains are overall more effective than deletions (p  = 8.5e-05) (**Figure 4A**). No clear pattern for CNV effect versus CNV-gene distance was observed, nor preference in terms of direct or inverse effect (**Figure 4B**).

**Figure 4C** lists the top associations detected for the set of new CNVs located within gene coding regions and **Figure 4D** shows examples of mRNA levels with respect to copy number states for nine new variants. BLMH encodes the enzyme Bleomycin hydrolase that is a cytoplasmic cysteine peptidase [25], [26] and has been associated with the risk of development of Alzheimer's disease [27]. ASF1B belongs to ASF family of proteins and is a histone chaperone that facilitates histone deposition, histone exchange and removal during nucleosomal assembly and disassembly [28]. Interestingly, ASF1A, an ASF1B homolog protein, was also detected as differentially expressed based upon a second variant. To rule out the possibility of potential mis-interpretation of the DNA data due to high homology between the two variants, we mapped the sequence of the 25-mers Affymetrix markers against the human genome and confirmed their specificity on chromosome 6 and chromosome 19, respectively. Other examples include lemur tyrosin kinase (LMTK2), and nuclear transport factor 2 (NUTF2) involved in cancer susceptibility [29] and diabetic retinopathy [30], respectively.

## Discussion

We present a novel computational framework, IgC2N, to identify and genotype copy number variants. We have applied IgC2N to genome-wide Affymetrix data for HapMap phase 3 samples. However, this approach is conceptually not restricted to array based data and can be applied to preprocessed sequencing data.

IgC2N is a three step procedure. The first step, Detection of Candidate CNV Loci, generates a list of putative CNVs. Unlike most common CNV detection methods, it does not detect CNV on a sample basis but combines information across samples, an approach which increases the detection power. The accuracy of CNV breakpoints will depend on the marker density of the platform used, e.g., a high resolution platform or deep DNA sequencing data will provide good accuracy. Even though not explicitly investigated, fine tuning of the significance threshold of the candidate CNV detection loci can be used to query complex CNVs [31] by comparing boundaries of overlapping variants. In addition, the approach does not impose any restriction on the number of covering markers for calling a CNV, which allows for the detection of smaller CNVs with poor marker coverage. Recent studies [32] have shown that smaller CNVs are currently being discovered. The second step, *CN Class Detection*, classifies individuals into copy number classes for the candidate CNVs generated from the first step via an EM approach to a Gaussian Mixture Model problem. We also conduct a recursive outlier detection step to detect rare CN classes or rare CNVs which the classification methods fails to identify. In the third step, we make a novel attempt to estimate the reference model bias by using the relative 1-CN class difference (described in details in Materials and Methods) between loci and some genetic information.

We evaluated the performance of IgC2N through a simulation study and assessed that it has at least 80% power to detect rare (1%) CNVs with sufficient marker coverage or sufficient variant size in datasets with larger sample size (N = 2000) while detecting common CNVs with similar size or marker coverage with datasets of smaller sample size (N = 200).

By applying IgC2N to the HapMap 3 dataset, 734 novel polymorphic loci were identified which were not reported in DGV (as of March, 2010). We characterize this set of novel CNVs based on MAF, size and type of polymorphism (deletion, gain or both). We found that the majority of novel deletions are rare (<5% MAF) while the majority of gains are common (10–30% MAF), possibly reflecting the fact that deletions are easier to detect than gains [22]. In terms of size, the majority of the novel CNVs detected by IgC2N are sCNVs, similar to the findings of Kato et al [32]. An overlap analysis of the novel CNVs with genes and exons showed that the size of a CNV does not increase the likelihood of gene overlap, whereas larger CNVs tend to overlap with more exons. We then investigated the mechanism of formation of these structural variants and found that sCNVs are relatively more likely to be formed by VNTR compared to CNVs while CNVs are more likely to be formed by NAHR. Overall NHR constitutes the major part of all CNV formation mechanisms which is consistent with previous findings [22].

To validate the novel CNVs detected by IgC2N, we queried high resolution NimbleGen data with 75 bp resolution [5]. 66.26% of CNVs (variants with size >1 Kb) were confirmed on a sample basis. We found a lower validation rate for CNVs of size ≤500 bp. Although one would expect a higher false positive rate for very small variants, it is also plausible that the larger probe size of the NimbleGen data (>60-mers versus 25-mers for the discovery platform) would be disadvantageous when positioned across breakpoints. Also, the smaller variants are enriched to be formed by VNTR or TEI and ensuing sequence complexities can explain the low validation rate. Ad hoc qPCR experiments would confirm or refute the existence of the variants that failed NimbleGen validation and also determine the accuracy of the genotyping step.

With the goal of evaluating the detection ability of IgC2N in comparison to other existing methods, we performed overlap analysis with the set of variants detected by IgC2N and the list of variants reported by McCarroll et al that implemented Birdsuite [19]. We were able to detect 86.87% of the CNVs reported by McCarroll et al [19] (applying constraints in terms of power as per IgC2N simulation), while McCarroll et al failed to detect 70.91% of the CNVs detected by IgC2N, 58.04% of which are reported in DGV. We looked at individual-level CN genotype comparison for overlapping CNVs and found that majority of CNVs show high level (between 90–100%) of concordance in genotypes across samples. As a surrogate measure for accuracy of the genotyping algorithm we evaluated Mendelian consistency in HapMap trios. The discordant rates of IgC2N were lower than Birdseye [19] and dChip [33] while being comparable to ÇOKGEN [11] as reported in [20]. These results demonstrate the detection ability and genotyping accuracy of IgC2N.

Finally, when assessing the functional impact of CNVs on the human transcriptome, we found that overall 4.4% gene transcript levels are significantly associated with CNVs at a false discovery rate of 10%, with 23% of the associations not being previously reported. In agreement with previous studies, investigation of the association between transcript and copy number changes in humans [8] and in mice [34] revealed greater functional impact from variants residing outside the protein coding gene locus. Interestingly, small variants were significantly more prone to affect transcript levels suggesting a preferential localization on (long distance) gene enhancers and repressors. In addition, variants involving gains were more likely to be effective than deletions. Based on the patterns of transcript levels versus observed copy number classes, it is apparent that different regulatory elements are partially controlled by genetic variants, either enhancers or repressors. Some examples of deletion/enhancer effect involve the regulation of pleckstrin homology domain containing, family F (with FYVE domain) member 1(PLEKHF1), and Parkinson disease (autosomal recessive, early onset) 7 (PARK7), and of farnesyl-diphosphate farnesyltransferase 1 (FDFT1) as deletion/repressor effect. Associations suggesting gain/repressor effects include BLMH, ASF1A and Mitochondrial ribosomal protein L17 (MRPL17). Gain/enhancer effects include NUTF2 and Transcription factor Dp-1 (TFDP1) (**Figure 4** and **File S1**). Interestingly, strong associations were detected involving outlier transcript levels and rare gain variant (**File S1)** as for chaperonin containing TCP1, subunit 6A (zeta 1) (CCT6A), complement factor D (adipsin) (CDF), and the gene coding the Insulin-like growth factor-binding protein 7 (IGFBP7), recently shown to alter the sensitivity to anticancer therapy [35].

The impact of genetic variants on gene expression represents one mechanism for phenotypic variation observed in humans and other species. To date the number of genetic regulatory effects is unknown, as the extent of genetic structural variants has only begun to be elucidated. SNPs and CNVs represent non-redundant of genetic variation as manifested by the fact that there is only partial overlap between gene expression-CNV and gene expression-SNP correlation [8]. This is not surprising as CNVs and SNPs are not in complete linkage disequilibrium [32]. Kasowski et al [36] demonstrated that a significant fraction (26%–35%) of inter-individual differences in transcription factor binding regions coincides with genetic variation loci, suggesting a crucial role of *cis* elements in the genetics of transcription factors. Altogether, there is increasing interest in identifying genetic variants that show regulatory effect and contribute to the explanation of phenotypic variation of humans. One might argue that discovery of CNV will plateau with the completion of the 1000 genome project (http://www.1000genomes.org/) [37] providing a comprehensive list of CNVs with accurate breakpoints. However, array based data from large collection of individuals would continue to be necessary in studying the relationship between CNVs and different human diseases owing to its cost-effectiveness and methodological improvements on CNV discovery and detection can accelerate the success of large scale disease susceptibility studies.

## Materials and Methods

### Dataset and data preprocessing analysis for 1250 HapMap individuals (discovery platform)

The raw data generated for HapMap Phase 2 individuals was obtained from Affymetrix (Affymetrix, Santa Clara, CA). Additional data from HapMap Phase 3 was obtained from the HapMap Consortium website. All raw data were generated on Affymetrix Genome-Wide Human SNP Array 6.0. The cohort includes 1250 individuals from 11 unique populations, representing several different ethnicities. Of these, we analyzed good quality data representing 87 individuals of African ancestry from Southwest USA (ASW); 178 Utah residents with Northern and Western European ancestry from the CEPH collection (CEU); 90 Han Chinese from Beijing, China (CHB); 90 Chinese from Metropolitan Denver, Colorado (CHD); 90 Gujarati Indians from Houston, Texas (GIH); 91 Japanese from Tokyo, Japan (JPT); 90 Luhya from Webuye, Kenya (LWK); 84 individuals of Mexican ancestry from Los Angeles, California (MEX); 179 Maasai from Kinyawa, Kenya (MKK); 90 Toscans from Italy (TSI); and 180 Yoruba from Ibadan, Nigeria (YRI). We also analyzed data for one non-HapMap individual from the Polymorphism Discovery Resource (NA15510) [5].

The raw intensity data were extracted from the CEL files and preprocessed as previously presented in Oldridge et al [16]. Briefly, raw data was preprocessed according to the Affymetrix CN5 method included in Affymetrix Power Tools (APT) (http://www.affymetrix.com/partners_programs/programs/developer/tools/powertools.affx). Following this step, data points were filtered out based on SNP call rate, call reproducibility, or marker specificity (i.e. markers mapping to more than 4 locations in the hg18 build of the human reference genome) as in Oldridge et al 2010 [16]. Preprocessed data was then segmented using the Circular Binary Segmentation (CBS) algorithm with recommended default settings in Olshen et al 2004 [38].

### Consortium dataset for a subset of 40 HapMap individuals (validation platform)

The preprocessed data, generated with the 42 million marker NimbleGen array set designed for CNV discovery on 19 HapMap CEU, 20 HapMap YRI, and a Polymorphism Discovery Resource individual (NA15510), was downloaded from the GSV. This set of samples is a subset of the 1250 HapMap samples described above.

### RNASeq datasets

mRNA data from 60 CEU and 69 YRI HapMap individuals generated by sequencing technology by Montgomery et al [6] and Pickrell et al [7] were queried to investigate the regulatory effects of CNVs. Sequencing data were originally generated using the Illumina Analyzer II with 36-base and 35 or 46-base pairs, respectively. YRI individual data were downloaded from http://eqtl.uchicago.edu, where CEU individual raw data were obtained from ArrayExpress under accession numbers E-MTAB-197.

### Reference model bias

Every array-based experiment suffers from some technical variation which is introduced due to the differential amount of DNA hybridizing for each marker. To eliminate the variation in the amount of marker-specific hybridization, the common practice is to take the ratio of the intensities with respect to a reference. The reference could be a single sample reference or averaged across several samples. Although this successfully eliminates the technical variation, it introduces a bias as the inferred copy number state would be only with respect to the reference model. In this paper, the bias will be referred to as the *reference model bias* (bias of the copy number state) introduced by the reference model and for a more detailed description refer to Oldridge et al 2010 [16].

Let us denote 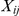 as the signal intensity of the 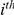 individual and the 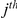 marker. One may construct a reference model as 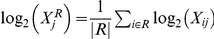 where *R* is considered as the reference set which could be a singleton and 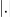 denotes its cardinality. The relative intensity (in the log_2_ scale) of each marker *j* with respect to the reference set is of interest. The hybridization intensity of each marker *j* depends on the amount of DNA hybridization and the copy number state of that genomic region. So it is natural to assume the following model:
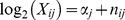
(1)where 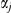 is the marker specific hybridization and 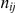 is the copy number state of the genomic region corresponding to the 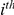 individual and 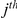 the marker. The general practice is to consider the relative intensity of each marker (*IR_ij_*) with respect to the reference set to cancel out the marker-specific hybridization
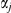
's. However, this introduces a bias represented by the second term in (2).
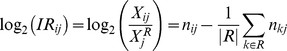
(2)This bias could lead to erroneous inference of copy number states, as depicted in **Figure 1**, unless explicitly modeled or accounted for. We will present here a strategy to detect CNV and provide CN calls accounting for this bias.

### IgC2N Computational Framework

The IgC2N framework has three conceptual steps. First, a set of candidate CNV loci is generated (a schema is presented in **Figure 1**). Secondly, the number of copy number states for each of these candidate CNV loci is determined which provides the set of CNV loci. And finally, we use step 2 to estimate the reference model bias and infer the CN states.

#### Step 1. *Detection of Candidate CNV Loci*

A score 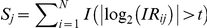 is applied for each marker along the genome. S_j_ counts the number of individuals that have signal intensity beyond a certain threshold t (user supplied) (**Figures 1A–C**). Then a genome-wide significance is calculated for each marker (**Figure 1D**). X and Y chromosomes are omitted in this evaluation to avoid gender complications. The p-values can be generated using either a permutation-based test or a binomial test (described below). Based on the p-values we compute the False Discovery Rates (Benjamini and Hochberg, 1995) or q-values for each marker.

*Permutation-based test:* The scores based on the null distribution are calculated as 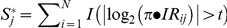 where 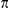 is the permutation group on 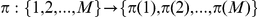 where *M* is the total number of markers. In other words, for each sample the score for all markers are permuted. A limitation of this approach is that the number of permutations is tied to the sample size and low sample size may not provide robust results.

*Binomial test:* Under the null hypothesis of no CNVs, we can assume 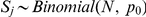 where *N* is the number of samples and *p_0_* is the probability of detecting a copy number event under the null hypothesis. The limitation of this approach is the choice of *p_0_*. We have used 
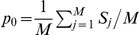
 which is the average score per sample (averages across all markers). We also perform simulations to see the correlation between the permutation-based p-values and the binomial p-values and evaluate our choice of *p_0_* (detail is provided in **File S1**).

Consecutive markers crossing the *q*-value threshold (say 10%) are deemed as candidate CNV loci (**Figure 1E**) and we call these markers significant markers for polymorphism. The breakpoints of the putative CNVs are calculated by the marker positions, that is, for a run of *n* significant markers the CNV locus is determined as [*p_1_*, *p_n_*] where *p_1_* and *p_n_* are the genomic coordinates of the first and last significant marker respectively of that run. Alternatively, one could calculate the breakpoints by considering the position of neighboring markers in order to account for the finite resolution of the platform. For example, one could consider the starting (ending) location as the mid-point between the first (last) significant marker of the current run and the previous (next) marker. Importantly, there are no restrictions on the minimum number of covering markers or on the size of putative CNV loci.

A comprehensive list of CNVs can be created by merging the IgC2N list with other known variants of interest. . However, in this manuscript we present results from the IgC2N detected list of CNVs only.

#### Step 2. *CN Class Detection*

The second step, *CN Class Detection*, generates copy number classes for the candidate CNVs generated from step 1 (**Figure 1H–I**). For each CNV and each individual a summary signal (e.g median) is calculated when more than one marker is present at the locus of interest. The following procedure is applied to the summary signal:

- Naïve CN Class Detection: A Gaussian mixture model with number of components (n.comp) = 1, 2, 3, 4, 5 and 6 is fitted to the data. To model the variance of different mixture component (or n.comp), the same variance for all n.comps except 2 and 3 is used. For n.comp = 2 or 3 estimation of different variance parameters for each component is allowed. The rationale behind this step is this distribution of deletions. In presence of deletions, the variances of the components are quite different and it is impractical to impose of restriction on them. A Gaussian Mixture Model is fit using the Expectation Maximization algorithm as implemented in the R library CNVtools [18]. The following tuning steps are applied:
  - If the EM algorithm does not converge for n.comp = 2 or 3 in (a), then the model is relaxed by assuming the variance of each component as equal (this is tailored for a CNV having gains only).
  - Out of the 6 GMMs fit to the data corresponding to different number of components the one with the lowest BIC (Bayesian Information Criterion, [18]) is selected.
  - If the EM algorithm for the selected n.comp did not converge, a mixture of “t” distributions is fitted which allows longer tails.
  - The predicted class (copy number) is obtained from the posterior probabilities. If there are overlapping intervals of the predicted classes, the n.comp is reduced by 1. If the EM algorithm does not converge with the reduced n.comp they are made non-overlapping by creating new classes.
- *Rare variant or CN Class detection*: A well-known attribute of any probabilistic classification technique is that new classes are not detected for few members. To enhance the chance of detecting rare CNVs and CN classes we perform a recursive outlier detection using Grubbs test [39] for single sample outlier detection. This is performed prior to removing CNVs with 1 detected CN class. Few examples indicating the benefits of the outlier detection step are provided in **File S1**.
- The differences between the median values (across of all samples) of the summary signals for each consecutive CNV class are also stored. We will refer to this distance between the centers of consecutive CN classes as 1-CN class difference here on (see **Figure 1H**). Note that the classification of each individual for each CNV does not correspond to the actual CNV genotype owing to the reference model bias. This naïve classification (or the predicted CNV class) is stored. Among the candidate CNV loci the ones which have more than one predicted class (by GMM) are retained and the rest removed.

#### Step 3. *Bias correction and Copy Number calls*

The third step, *Bias correction and Copy Number calls*, corrects the reference model bias and infers the true copy number genotype. The following steps are undertaken:

- *Expected Direction of Bias*: If the major (in terms of frequency) predicted CN class (from Step 2) is also the biggest (or smallest) integer CN class and the median of this major class close to “0” (the expected median of the CN="2” class), say |distance|≤0.25, then we hypothesize this class to be copy number state “2”. The other classes are inferred relative to this class. This step is motivated for the [0,1,2] and [1,2] ([2,3],[2,3,4]) CN genotype models. For example, if there are frequent deletions the reference model will be expected to have lower intensity signal thereby inducing a positive location bias. The same argument applies in the case of gains and a negative location bias.
- *Zero Presence Test*: In the presence of a CNV with hemizygous deletions (CN="1”) and under the assumption of Hardy-Weinberg Equilibrium, we should expect HW proportions for CN="0”, “1”, “2”. So, we test the observed “0” proportion with the expected HW proportion *p^2^* (where *p* is the allelic proportion of the deletion allele) using a binomial test.
- *1 CN Class Distribution*: Finally we use the difference in medians of consecutive copy number classes and compare it with the 1 CN Class distribution of Step 2c. Using the 1-CN difference distribution (currently that of known CNVs used) we fit a Gaussian Mixture Model to four mixtures (0–1, 1–2, 2–3 and 3–4) (**Figure 1G**).
  - For each CNV the 1-CN difference is computed based on the predicted CNV class; its probability of belonging to a particular 1-CN difference cluster (0–1, 1–2, 2–3 or 3–4) is calculated.
  - Build a (*k*−1)×4 matrix (*k* CN classes predicted and hence *k*−1 differences and 4 corresponding to 0–1,1–2,2–3,3–4) of probabilities. The highest probability of the matrix is chosen and the corresponding column is removed. The next highest probability is chosen for the next assignment.
  - If any assigned class has the copy number class 2 and has more than 5% frequency then this class is used to estimate the true 0. If there is no CN 2 state, the most frequent state is used to perform the centering. If the most frequent state is 0 then second most frequent state is used for centering.

### Simulation Study

The goal of this simulation study was to evaluate the false positive rate and the power to detect CNVs using IgC2N. A few CNV characteristics were identified to be relevant for a CNV detection study: a) CNV size, b) the frequency of the CNV (in the population) and c) its type, namely deletion, gain or both. We considered five different CNV sizes: very small (<<1 kb), small (around 1 kb), medium (5 kb), large (50–100 kb) and very large (>200 kb). For each size we considered different incidences of each CNV namely- very rare (1%), rare (5%) and frequent (15%). For each size and incident CNV we considered three types of CNVs: del (deletion only), gain (gain only) and del-gain (both deletion and gain). For each of these 45 (5×3×3) types of CNVs, we considered a duplicate having 90 total CNVs in each dataset. Note that the size of a CNV is inherently related to the number of markers covering the CNV which depends on the marker distribution of the specific platform in question. We considered four different sample sizes 200, 400, 800 and 2000. Since the goal of our simulation study was to understand the CNV detection capabilities of IgC2N, the third step (CNV genotyping) is omitted to reduce computational burden. Since simulated data is less noisy compared to real data, the smoothing step (by segmenting) is omitted. For each sample size, 100 datasets were generated and analyzed by IgC2N. To investigate if 100 datasets are adequate for the simulations, we generated 500 datasets for sample size of 200 and monitored the behavior of false positive rate and overall power on increasing number of datasets. We noticed that both false positive rate and overall power (data not shown) stabilize after 100 datasets. Hence we found it adequate to generate 100 datasets for all sample sizes. For each iteration or each simulated dataset, we compared the number of CNVs detected with the true CNV list by 1 bp overlap criterion. The number of false positive rate (FPR) for each dataset was calculated by counting the number of CNV detected by IgC2N which do not overlap with the true CNV list and dividing it by the total number of detected CNVs (by IgC2N). The reported FPR is the average FPR across 100 datasets. On the other hand, the empirical power for detecting each CNV was calculated on a CNV basis. The fraction of datasets where a particular CNV is detected by IgC2N is considered as the empirical power of that CNV. The 90 true CNVs are categorized according to different characteristics and the power of each category is the average power across those CNVs.

The simulation study was restricted to chromosome 1 of the human genome. The marker distribution on chromosome 1 was based on the Affymetrix Genome-Wide Human SNP Array 6.0 marker distribution as we evaluate IgC2N on HapMap samples run on the Affymetrix SNP Array 6.0 platform.

### Independent Platform Validation

To validate the novel CNVs on the basis of the NimbleGen dataset, we adopted the following criteria. A CNV is considered validated if the correlation between the Affymetrix median signal and a NimbleGen marker within the predicted genomic location is 0.5 or more. Ninety-seven CNVs were validated using this criterion. When the correlation falls between [0.3, 0.5), we adopted the following strategy: i) consider the outliers (observations falling outside 1.5× Inter-Quartile-Range) for the NimbleGen marker data for each CNV and based upon the outlier sign, we consider it as gains or deletions; ii) for each CNV we consider the concordance in the existence of gain or deletion between the Affymetrix and NimbleGen data. If concordant, the CNV is considered validated. Finally, two independent observers (F.D and S.B) visually inspected the validated and non-validated CNVs to correct for uneven calls. This procedure was applied to all but 94 novel CNVs (no NimbleGen coverage).

### Mechanism of formation of variants and gene-exon overlap

In order to characterize CNV in terms of formation mechanisms we applied classification methods similar to those implemented in other studies [5], [22]. The CNV formation mechanisms are usually classified into 4 major groups: (1) non-allelic homologous recombination (NAHR), which is homologous recombination between homologous sequences in different genomic positions; (2) non-homologous recombination (NHR) that can proceed as one of the ways for DNA double-strand break repair, and is implemented either through non-homologous end joining or through microhomology-mediated end joining [40]; (3) variable number of tandem repeats (VNTR), which result from expansion or contraction of repeat elements; (4) transposable elements insertion (TEI), which includes different classes of transposable elements, mostly SINE, LINE, but also other smaller transposon groups.

To infer the mechanisms of CNV formation, we extracted the CNV coordinates applying 500 bp flanks, which we will call extended CNV loci. The choice of flank size was based on the estimation of breakpoint accuracy, which is to be estimated on average 1 kb for Affymetrix Genome-Wide Human SNP Array 6.0. The parameter sensitivity analysis performed in [22] with fixed size flanking regions showed that within an extended parameter space and in the proximity of the chosen parameters the mechanism formation results are relatively insensitive to adjustment. To account for the lower breakpoint accuracy of the platform used in our study, we extended the parameter sensitivity analysis to include flanking regions ranging within 100–2,000 bp. Our results demonstrated relative insensitivity to the size of flanking regions for VNTR and NAHR events, however, for TEI the maximum frequency was observed around 500 bp. Further expansion of the area to 2000 bp decreased the frequency.

The extended CNV loci were further investigated for the presence of characteristic features for each type of mechanisms. To be classified as an VNTR-mediated event, we required >50% of an extended CNV locus to be covered by short tandem repeats, loci of low complexity or satellite DNA as it is defined by RepeatMasker (http://www.repeatmasker.org, ver. 3.2.8, RepBase library Release 20090604). NAHR-mediated events were inferred from a search of homologous blocks in the areas around the breakpoints (+/−500 bp) of minimum length of 30 bp and minimum sequence identity of 85%. TEI events were identified by the presence of transposable elements that must cover at least 50% of the extended CNV locus. We allowed for 500 bp distance between transposable elements. Transposable elements were identified with the program RepeatMasker (http://www.repeatmasker.org). Finally, all CNVs lacking classification features described above were classified as NHR-mediated events.

### Gene expression data analysis

CEU sequencing data were processed applying RSEQtools [41]. We performed *cis* analysis applying 1 Mb flanks to each variant accordingly to [8] and tested for dosage effect and allelic effect of transcript levels versus the copy number states in a linear model. In addition, we evaluated the correlation between the transcript levels and the log2 values of the copy number intensity ratios, and, for CNVs with two copy number states, the Wilcoxon's signed rank test was used. In the absence of normality of transcript levels (evaluated by Shapiro-Wilk's test), the last two tests were taken into account for the evaluation of the functional impact of the variants. In order to account for multiple hypotheses testing, we computed the False Discovery Rates (Benjamini and Hochberg, 1995). The analysis was independently performed for the two datasets, CEU and YRI. The distance between variants and genes was defined as the absolute difference between the midpoint of the gene transcription starting and ending sites and the midpoint of the variant genomic coordinates. Proportion test was applied to asses for significant differences in functional variant proportions with respect to size and type of polymorphism.

All coordinates are expressed using the hg18 assembly. IgC2N code and test dataset is available at http://icb.med.cornell.edu/faculty/demichelis/lab/~IgC2N.html or upon request to the authors. IgC2N has been developed in R 2.9.0. The new CNVs information is available from DGV (Database of Genomic Variants).

## Supporting Information

File S1 File S1 contains nine supplemental figures, nine supplemental tables and a supporting methods section.
(DOC)
