# Supplementary material for: A Computational Framework Discovers New Copy Number Variants with Functional Importance
Source: PLoS One. 2011 Mar 29;6(3):e17539. doi: 10.1371/journal.pone.0017539 (PMC3066184; doi:10.1371/journal.pone.0017539)
Supplement: File S1 — contains nine supplemental figures, nine supplemental tables and a supporting methods section. (DOC) [file pone.0017539.s001.doc]

**SUPPORTING INFORMATION**

**A Computational Framework Discovers New Copy Number Variants with Functional Importance**

Samprit Banerjee^1^, Derek Oldridge^2^, Maria Poptsova^2^, Wasay M. Hussain^2^, Dimple Chakravarty^2^, and Francesca Demichelis^2,3*^

^1^Department of Public Health, ^2^Department of Pathology and Laboratory Medicine, ^3^Institute for Computational Biomedicine, Weill Cornell Medical College, New York, NY, USA

**Supporting Information**

This document contains supporting information including figures, tables and methods and is organized as follows:

1. **Supporting Figures:**
2. **Figure S1: Stabilization of False Positive Rates over simulated datasets.** The false positive rate (FPR) for simulation of N=200 plotted against the first 25 up to 500 datasets. The FPR stabilizes after 100 datasets.
3. **Figure S2: False Positive Rate for IgC2N**. The box plots represent FPR calculated for each of the 100 simulated datasets for each sample size.
4. **Figure S3: Concordance of CN genotypes.** The histogram gives the distribution of proportion of concordance in CN genotypes determined by IgC2N and Birdsuite. This is evaluated on a list of CNVs, which are detected by both methods on HapMap data.
5. **Figure S4**: **Mendelian Discordance**. Average discordance rate (percentage of discordant HapMap trios) for CNVs in different strata of polymorphism frequency.
6. **Figure S5: Examples of significant and concordant gene-variant associations in two populations.** Left and right panels correspond to Caucasian (CEU) and Yorubian (YRI) populations, respectively.
7. **Figure S6: Examples of outliers expression levels associated with non-common copy number gains.** The following genes are represented: chaperonin containing TCP1, subunit 6A (zeta 1) (CCT6A), complement factor D (adipsin) (CDF), and the gene coding the Insulin-like growth factor-binding protein 7 (IGFBP7).
8. **Figure S7: Examples of known variants and previously unreported association with gene transcript levels.** Transcription factor Dp-1 (TFDP1, upper panels) and Mitochondrial ribosomal protein L17 (MRPL17, lower panels).
9. **Figure S8**: **Examples of the outlier detection step.** Left panel shows an example of a variant which would not have been detected as a variant by IgC2N without the outlier detection step owing to the low frequency of polymorphism. Right panel shows an example of a variant where a rare CN class (homozygous deletion) would not have been detected by IgC2N without the outlier detection step.
10. **Supporting Tables:**
    1. **Table S1**: False Positive Rate of Simulations
    2. **Table S2:** Frequency of CNV for all (2497) IgC2N detected CNVs
    3. **Table S3**: Size in Kb for all (2497) IgC2N detected CNVs
    4. **Table S4**: Gene or Exon overlap for all (2497) IgC2N detected CNVs
    5. **Table S5**: Mechanism of formation for all (2497) IgC2N detected CNVs
    6. **Table S6**: Top Gene-Variant Associations in YRI population
    7. **Table S7**: Significant Gene-Variant Associations in CEU population (10% FDR)
11. **Supporting Methods:**
    1. *Permutation and Binomial Test Comparison:*
       1. **Table S8**: Comparison of P-values Between Permutation and Binomial Tests
    2. *Genotyping Errors*
       1. **Table S9**: Genotyping Errors
       2. **Figure S9: Examples of 0-1 and 3-4 genotyping errors.** Left panel shows an example of 3-4 genotyping error while the right panel shows that of 0-1 genotyping error.

**Supporting Figures**

**Figure S1: Stabilization of False Positive Rates over simulated datasets.** The false positive rate (FPR) for simulation of N=200 plotted against the first 25 up to 500 datasets. The FPR stabilizes after 100 datasets.

**Figure S2: False Positive Rate for IgC2N**. The box plots represent FPR calculated for each of the 100 simulated datasets for each sample size.


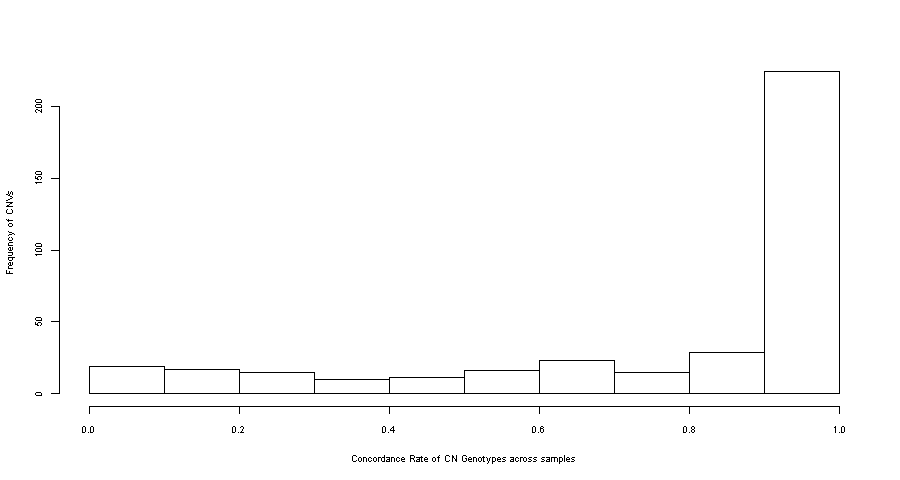


**Figure S3: Concordance of CN genotypes.** The histogram gives the distribution of proportion of concordance in CN genotypes determined by IgC2N and Birdsuite. This is evaluated on a list of CNVs, which are detected by both methods on HapMap data.

**
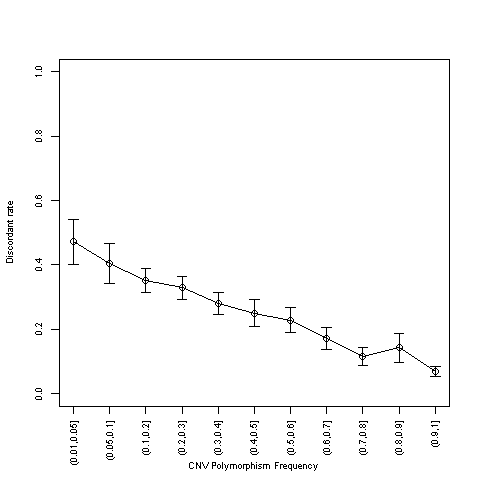
**

**Figure S4**: **Mendelian Discordance**. Average discordance rate (percentage of discordant HapMap trios) for CNVs in different strata of polymorphism frequency.


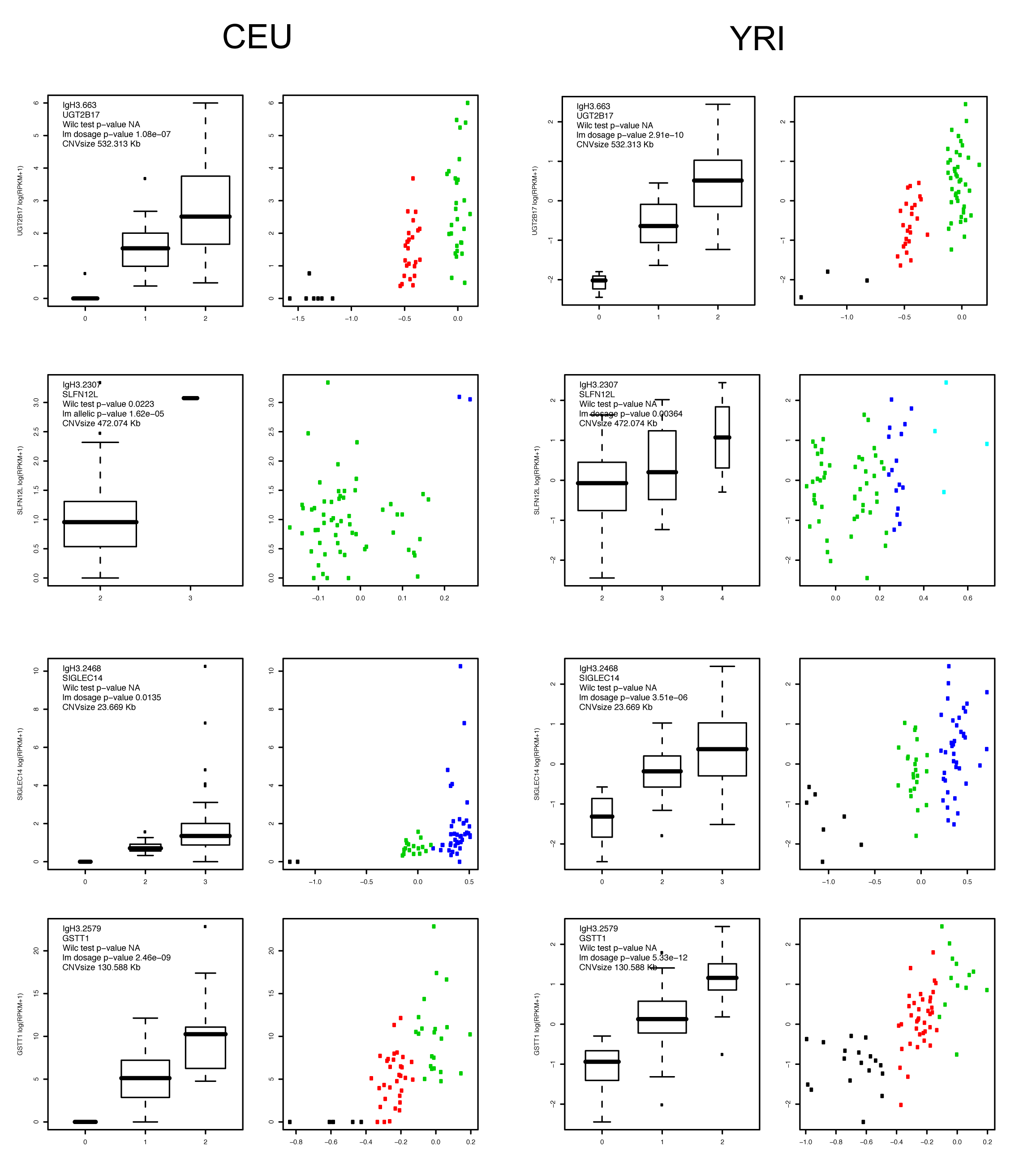


**Figure S5: Examples of significant and concordant gene-variant associations in two populations.** Left and right panels correspond to Caucasian (CEU) and Yorubian (YRI) populations, respectively.


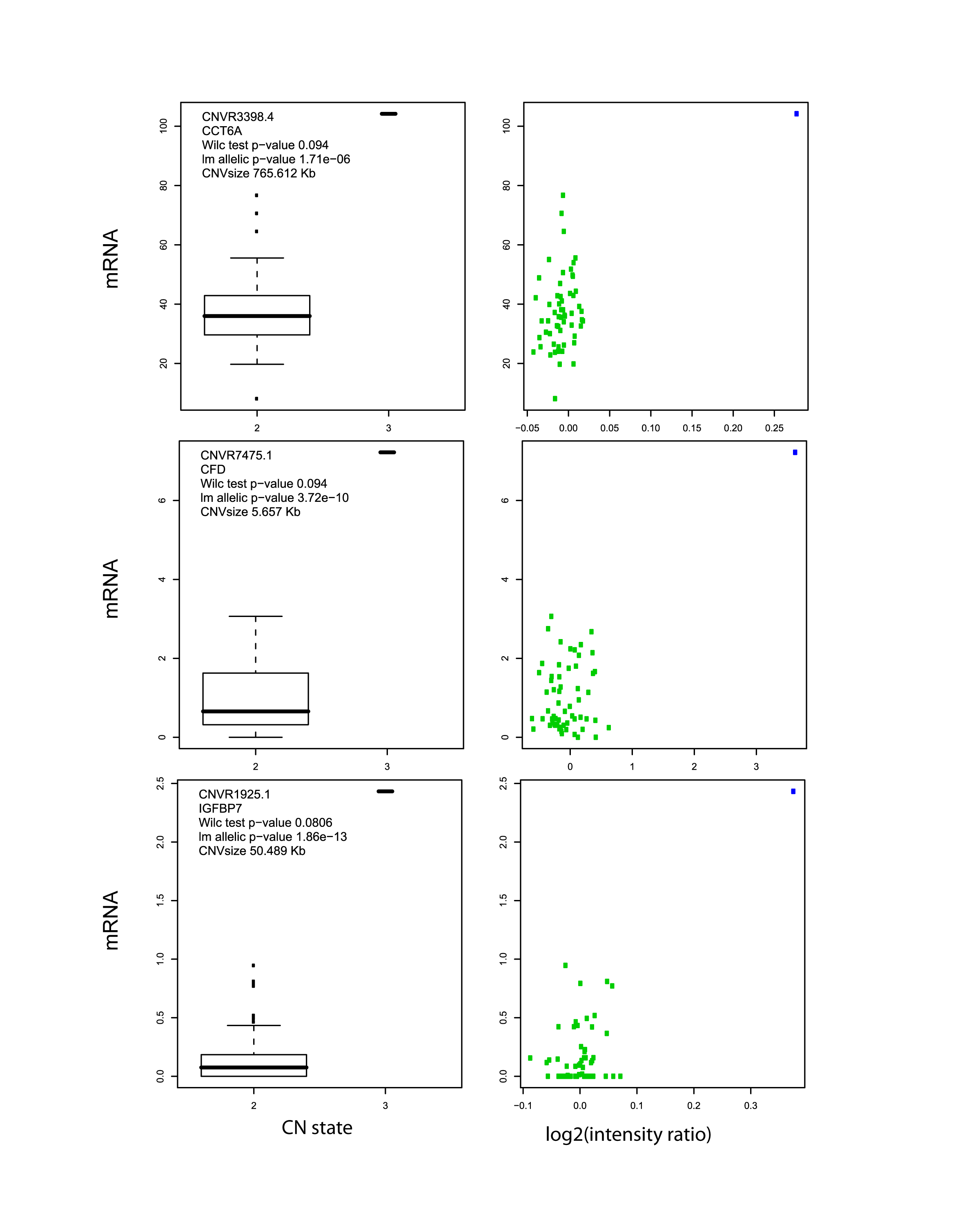


**Figure S6: Examples of outliers expression levels associated with non-common copy number gains.** The following genes are represented: chaperonin containing TCP1, subunit 6A (zeta 1) (CCT6A), complement factor D (adipsin) (CDF), and the gene coding the Insulin-like growth factor-binding protein 7 (IGFBP7).


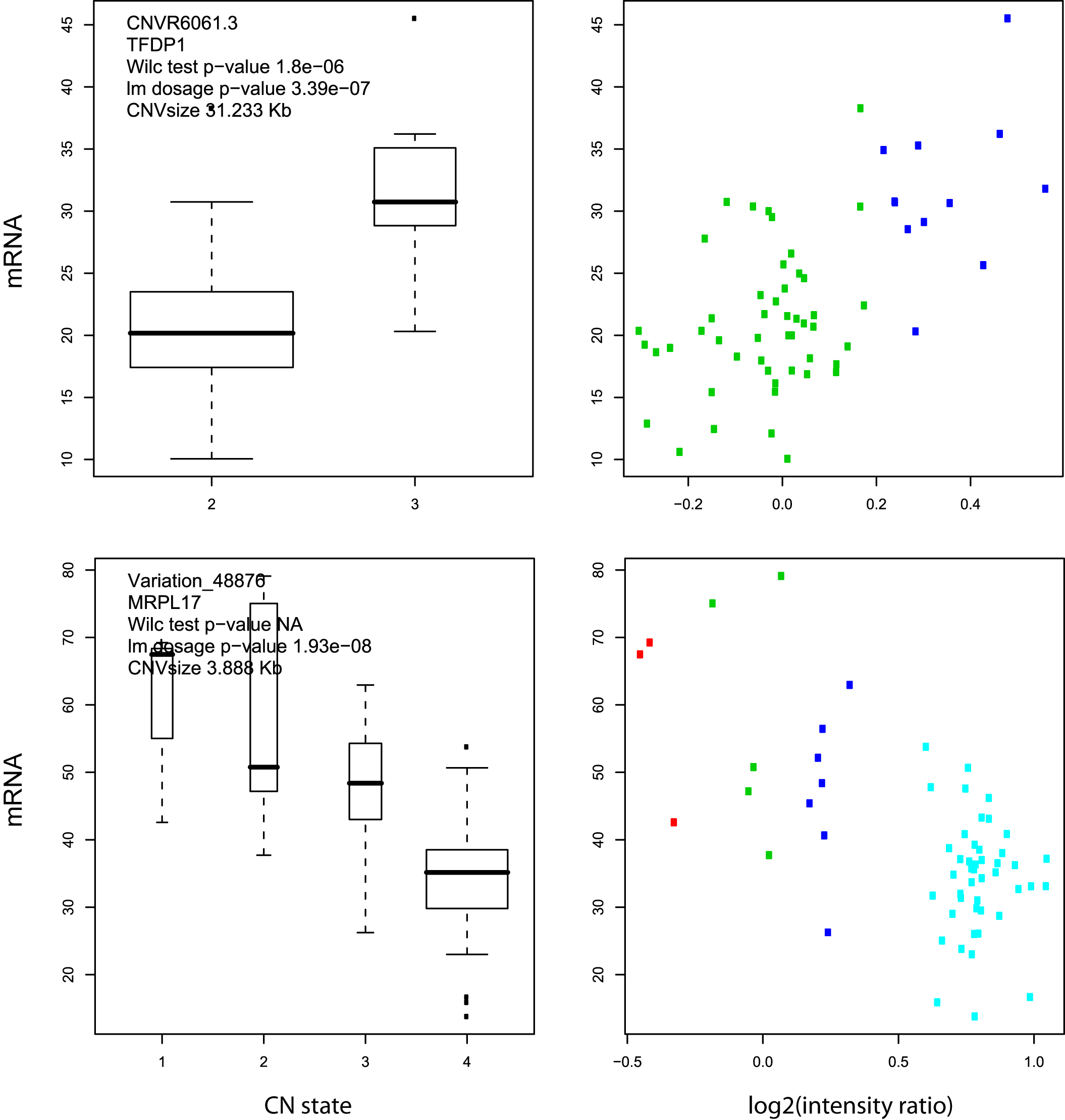


**Figure S7: Examples of known variants and previously unreported association with gene transcript levels.** Transcription factor Dp-1 (TFDP1, upper panels) and Mitochondrial ribosomal protein L17 (MRPL17, lower panels).

|  |  |
| --- | --- |

**Figure S8**: **Examples of the outlier detection step.** Left panel shows an example of a variant which would not have been detected as a variant by IgC2N without the outlier detection step owing to the low frequency of polymorphism. Right panel shows an example of a variant where a rare CN class (homozygous deletion) would not have been detected by IgC2N without the outlier detection step.

**Supporting Tables**

**Table S1:** False Positive Rate of Simulations

|  | **Min.** | **1st Qu.** | **Median** | **Mean** | **3rd Qu.** | **Max.** |
| --- | --- | --- | --- | --- | --- | --- |
| **FPR (N=200)** | 0 | 0 | 0.00813 | 0.007213 | 0.009615 | 0.04098 |
| **FPR (N=400)** | 0 | 0 | 0.003548 | 0.002582 | 0.004012 | 0.01261 |
| **FPR (N=800)** | 0 | 0.002049 | 0.002963 | 0.002852 | 0.003098 | 0.01156 |
| **FPR (N=2000)** | 0.008929 | 0.009975 | 0.01036 | 0.01134 | 0.01099 | 0.02941 |

**Table S2:** **Frequency of CNV for all (2497) IgC2N detected CNVs**

| **MAF** | **(0,0.05]** | **(0.05,0.1]** | **(0.1,0.3]** | **(0.3,0.5]** | **Total** |
| --- | --- | --- | --- | --- | --- |
| **All** | 1760 (70.48%) | 229 (9.17%) | 397 (15.9%) | 111 (4.45%) | 2497 (100%) |
| **Del** | 834 (67.2%) | 171 (13.78%) | 198 (15.95%) | 38 (3.06%) | 1241 (100%) |
| **Gain** | 154 (37.93%) | 15 (3.69%) | 164 (40.39%) | 73 (17.98%) | 406 (100%) |
| **Del/Gain** | 105 (57.38%) | 43 (23.5%) | 35 (19.13%) | 0 (0%) | 183 (100%) |
| **≤5%** | 667 (100%) | 0 (0%) | 0 (0%) | 0 (0%) | 667 (100%) |

**Table S3:** **Size in Kb for all (2497) IgC2N detected CNVs**

|  | **sCNVs** | **CNV** | | |  |
| --- | --- | --- | --- | --- | --- |
| **Size in Kb** | (0,1] | (1,10] | (10,100] | >100 | Total |
| **All** | 1085 (43.45%) | 871 (34.88%) | 435 (17.42%) | 106 (4.25%) | 2497 (100%) |
| **Del** | 657 (52.94%) | 427 (34.41%) | 136 (10.96%) | 21 (1.69%) | 1241 (100%) |
| **Gain** | 168 (41.38%) | 149 (36.7%) | 71 (17.49%) | 18 (4.43%) | 406 (100%) |
| **Del/Gain** | 111 (60.66%) | 60 (32.79%) | 10 (5.46%) | 2 (1.09%) | 183 (100%) |
| **≤5%** | 149 (22.34%) | 235 (35.23%) | 218 (32.68%) | 65 (9.75%) | 667 (100%) |

**Table S4: Gene or Exon overlap for all (2497) IgC2N detected CNVs**

|  | **sCNVs** | **CNV** | |  |
| --- | --- | --- | --- | --- |
| **Size in Kb** | (0,1] | (1,10] | (10,100] | Total |
| **Gene Overlap** | 396 (36.5%) | 290 (33.3%) | 159 (36.55%) | 84 (79.25%) |
| **Exon Overlap** | 24 (2.21%) | 72 (8.27%) | 100 (22.99%) | 84 (79.25%) |
| **All** | 1085 (43.45%) | 871 (34.88%) | 435 (17.42%) | 106 (4.25%) |

**Table S5: Mechanism of formation for all (2497) IgC2N detected CNVs**

|  | **sCNVs** | **CNV** | | |  |
| --- | --- | --- | --- | --- | --- |
| **Size in Kb** | (0,1] | (1,10] | (10,100] | >100 | Total |
| **NAHR** | 0 (0%) | 103 (11.83%) | 41 (9.43%) | 10 (9.43%) | 154 (6.17%) |
| **NHR** | 730 (67.28%) | 596 (68.43%) | 386 (88.74%) | 94 (88.68%) | 1806 (72.33%) |
| **TEI** | 243 (22.4%) | 167 (19.17%) | 7 (1.61%) | 0 (0%) | 417 (16.7%) |
| **VNTR** | 112 (10.32%) | 5 (0.57%) | 1 (0.23%) | 2 (1.89%) | 120 (4.81%) |
| **All** | 1085 (43.45%) | 871 (34.88%) | 435 (17.42%) | 106 (4.25%) | 2497 (100%) |

**Table S6: Top Gene-Variant Associations in YRI population**

| **SYMBOL** | **ID** | **Test (D=Dosage, A = Allelic)** | **Min p-value** | **FDR (q-value)** | **Correlation** | **Distance between Variant Gene Centers (Kb)** | **Variant Size (Kb)** | **Chromosome of variant** | **Variant Start Location** | **Variant End Location** | **Novel CNV (by IgC2N)** | **Validated by Nimblegen Platform** | **InStrangerSNP** | **InStrangerCNV** |
| --- | --- | --- | --- | --- | --- | --- | --- | --- | --- | --- | --- | --- | --- | --- |
| GSTT1 | IgH3.2579 | D | 5.33E-12 | 1.83E-07 | 0.689 | 173.772 | 130.588 | 22 | 22601733 | 22732321 | 0 |  | CEU|CHB|JPT|YRI |  |
| RRP7A | IgH3.2597 | D | 2.35E-10 | 3.09E-06 | 0.706 | 56.897 | 74.083 | 22 | 41222277 | 41296360 | 0 |  |  |  |
| UGT2B17 | IgH3.663 | D | 2.91E-10 | 3.33E-06 | 0.679 | 424.191 | 532.313 | 4 | 68943134 | 69475447 | 0 |  | CEU|CHB|JPT | CEU|CHB|JPT|YRI |
| SIGLEC5 | IgH3.2468 | D | 2.27E-09 | 1.95E-05 | -0.608 | 4.2875 | 23.669 | 19 | 56823613 | 56847282 | 0 |  |  | CEU|YRI |
| OR7D2 | IgH3.2427 | D | 8.19E-09 | 5.62E-05 | -0.537 | 24.9455 | 3.449 | 19 | 9135660 | 9139109 | 0 |  |  |  |
| GSTM1 | IgH3.75 | D | 3.79E-08 | 0.000216883 | 0.805 | 29.0895 | 32.552 | 1 | 110022101 | 110054653 | 0 |  | CEU|CHB|JPT|YRI |  |
| SERHL | IgH3.2597 | D | 1.26E-07 | 0.00061803 | 0.587 | 47.283 | 74.083 | 22 | 41222277 | 41296360 | 0 |  |  |  |
| FLJ26850 | IgH3.2466 | D | 2.32E-07 | 0.000995716 | -0.476 | 3.7645 | 0.598 | 19 | 55250339 | 55250937 | 0 |  |  |  |
| UPK3B | IgH3.1186 | D | 9.96E-07 | 0.003799743 | 0.572 | 336.806 | 526.605 | 7 | 75912904 | 76439509 | 0 |  |  |  |
| FYCO1 | IgH3.432 | D | 2.10E-06 | 0.007210356 | 0.57 | 50.535 | 1.877 | 3 | 46024828 | 46026705 | 0 |  |  |  |
| SIGLEC14 | IgH3.2468 | D | 3.51E-06 | 0.010955995 | 0.516 | 28.002 | 23.669 | 19 | 56823613 | 56847282 | 0 |  |  |  |
| LRP5L | IgH3.2582 | D | 5.25E-06 | 0.015021574 | 0.475 | 231.1 | 268.767 | 22 | 23986238 | 24255005 | 0 |  |  |  |
| RHD | IgH3.24 | D | 1.49E-05 | 0.037922811 | 0.492 | 92.222 | 95.086 | 1 | 25455866 | 25550952 | 0 |  |  |  |
| TBC1D5 | IgH3.412 | D | 1.82E-05 | 0.044635535 | 0.541 | 1250.294 | 0.623 | 3 | 16215548 | 16216171 | 0 |  |  |  |
| ZFYVE1 | IgH3.2075 | D | 3.17E-05 | 0.071371826 | -0.28 | 957.5515 | 2.135 | 14 | 73473283 | 73475418 | 1 | 0 |  |  |
| GBA | IgH3.102 | D | 4.80E-05 | 0.098338552 | 0.539 | 24.4635 | 11.916 | 1 | 153455772 | 153467688 | 0 |  |  |  |
| HSF2BP | IgH3.2563 | D | 5.06E-05 | 0.102197198 | 0.434 | 44.331 | 1.939 | 21 | 43795289 | 43797228 | 0 |  | YRI |  |
| CDH24 | CNVR6087.1 | A | 8.10E-05 | 0.142022225 | 0.111 | 416.6425 | 0.459 | 14 | 23008220 | 23008679 | 0 |  |  |  |
| ZNF500 | IgH3.2193 | A | 9.74E-05 | 0.159434757 | -0.403 | 545.3835 | 0.537 | 16 | 5294643 | 5295180 | 0 |  |  |  |
| OR7D2 | IgH3.2426 | D | 0.000103 | 0.164862502 | 0.492 | 44.6515 | 0.225 | 19 | 9114342 | 9114567 | 1 | 1 |  |  |
| DHTKD1 | Variation_29583 | A | 0.000109 | 0.170417003 | 0.331 | 69.5995 | 64.512 | 10 | 12140741 | 12205253 | 0 |  |  |  |
| SNORD76 | IgH3.119 | D | 0.000113 | 0.17397895 | 0.424 | 957.823 | 5.515 | 1 | 173063016 | 173068531 | 0 |  |  |  |
| BAMBI | IgH3.1631 | D | 0.000134 | 0.191030256 | 0.433 | 154.022 | 2.001 | 10 | 29164174 | 29166175 | 1 | 1 |  |  |
| PRPS1L1 | IgH3.1138 | D | 0.000164 | 0.211426849 | 0.336 | 734.149 | 0.391 | 7 | 17299514 | 17299905 | 0 |  |  |  |
| OR2T3 | IgH3.203 | D | 0.000183 | 0.222441143 | -0.41 | 164.1765 | 254.664 | 1 | 246666908 | 246921572 | 0 |  |  |  |
| RASSF7 | IgH3.1713 | A | 0.000189 | 0.225662475 | 0.384 | 124.841 | 0 | 11 | 427656 | 427656 | 0 |  |  |  |
| ZSCAN23 | CNVR2825.1 | D | 0.000196 | 0.229279297 | 0.373 | 432.9165 | 0.779 | 6 | 28947140 | 28947919 | 0 |  |  |  |
| SYCP3 | CNVR5642.1 | A | 0.000203 | 0.232752481 | 0.345 | 892.721 | 0.696 | 12 | 99759594 | 99760290 | 0 |  |  |  |
| CHRNA3 | CNVR6468.1 | D | 0.000213 | 0.237481502 | 0.288 | 903.445 | 1.149 | 15 | 77591716 | 77592865 | 0 |  |  |  |
| IL19 | IgH3.163 | A | 0.000242 | 0.249829581 | -0.116 | 952.82 | 0.114 | 1 | 204108129 | 204108243 | 1 | 1 |  |  |
| WDR25 | Variation_55232 | D | 0.00026 | 0.25661429 | -0.472 | 75.284 | 0.782 | 14 | 99914556 | 99915338 | 0 |  |  |  |
| FAM128A | IgH3.317 | D | 0.000262 | 0.257331509 | 0.529 | 43.5445 | 13.855 | 2 | 131925651 | 131939506 | 0 |  |  |  |
| ANXA2 | IgH3.2147 | D | 0.000263 | 0.257687511 | 0.404 | 506.3325 | 0.614 | 15 | 57946034 | 57946648 | 1 | 1 | JPT |  |
| DYM | CNVR7328.1 | A | 0.000276 | 0.262163267 | -0.217 | 830.1195 | 0.469 | 18 | 45862977 | 45863446 | 0 |  |  |  |
| HLCS | IgH3.2557 | D | 0.000292 | 0.267310075 | -0.347 | 112.5755 | 6.365 | 21 | 37280490 | 37286855 | 0 |  |  |  |
| HHIPL2 | IgH3.177 | D | 0.000324 | 0.27656025 | 0.343 | 334.7855 | 2.17 | 1 | 220441445 | 220443615 | 0 |  |  |  |
| LMNB2 | CNVR7483.1 | A | 0.000324 | 0.27656025 | -0.337 | 100.2605 | 5.94 | 19 | 2296270 | 2302210 | 0 |  |  |  |
| RFPL3 | Variation_42630 | A | 0.000345 | 0.281980086 | 0.294 | 539.927 | 0.513 | 22 | 31625684 | 31626197 | 0 |  |  |  |
| CLCC1 | IgH3.74 | D | 0.00036 | 0.285577872 | -0.404 | 7.0765 | 1.291 | 1 | 109298365 | 109299656 | 0 |  |  |  |
| PRKG1 | IgH3.1648 | D | 0.000368 | 0.287411414 | -0.379 | 405.327 | 3.444 | 10 | 52669510 | 52672954 | 0 |  |  |  |
| HLA-DRB5 | IgH3.985 | D | 0.000384 | 0.290913539 | 0.497 | 159.4045 | 194.936 | 6 | 32537621 | 32732557 | 0 |  | YRI | CHB|JPT|YRI |
| UHRF1BP1 | CNVR2857.1 | D | 0.000394 | 0.292997662 | -0.345 | 103.029 | 0.966 | 6 | 34807974 | 34808940 | 0 |  |  |  |
| STK10 | Variation_51621 | D | 0.000452 | 0.303719082 | -0.331 | 867.218 | 2.165 | 5 | 172343115 | 172345280 | 0 |  |  |  |
| COL5A2 | IgH3.357 | D | 0.000465 | 0.305844515 | -0.377 | 409.409 | 13.461 | 2 | 189276189 | 189289650 | 0 |  |  |  |
| WARS | Variation_55232 | D | 0.000472 | 0.306952152 | -0.417 | 23.3865 | 0.782 | 14 | 99914556 | 99915338 | 0 |  |  |  |
| JMJD7 | Variation_40086 | D | 0.000477 | 0.307728167 | -0.35 | 265.64 | 0.679 | 15 | 39647025 | 39647704 | 0 |  |  |  |
| PLA2G4B | Variation_40086 | D | 0.000477 | 0.307728167 | -0.35 | 276.2845 | 0.679 | 15 | 39647025 | 39647704 | 0 |  |  |  |
| JMJD7-PLA2G4B | Variation_40086 | D | 0.000477 | 0.307728167 | -0.35 | 270.9205 | 0.679 | 15 | 39647025 | 39647704 | 0 |  |  |  |

**Table S7: Significant Gene-Variant Associations in CEU population (10% FDR)**

| **SYMBOL** | **ID** | **Test (D=Dosage, A = Allelic)** | **Min p-value** | **FDR (q-value)** | **Correlation** | **Distance between Variant Gene Centers (Kb)** | **Variant Size (Kb)** | **Chromosome of variant** | **Variant Start Location** | **Variant End Location** | **Novel CNV (by IgC2N)** | **Validated by Nimblegen Platform** | **InStrangerSNP** | **InStrangerCNV** |
| --- | --- | --- | --- | --- | --- | --- | --- | --- | --- | --- | --- | --- | --- | --- |
| S100A7 | IgH3.100 | A | 0 | 0 | -0.404 | 559.5075 | 0.247 | 1 | 151138918 | 151139165 | 0 |  |  |  |
| KRTAP2-4 | IgH3.2311 | A | 0 | 0 | -0.535 | 162.688 | 6.221 | 17 | 36641074 | 36647295 | 0 |  |  |  |
| KRTAP4-3 | IgH3.2311 | A | 0 | 0 | -0.535 | 60.4845 | 6.221 | 17 | 36641074 | 36647295 | 0 |  |  |  |
| FLJ46361 | Variation_33232 | A | 4.00E-57 | 3.46E-53 | -0.588 | 656.073 | 0.654 | 10 | 125183075 | 125183729 | 0 |  |  |  |
| KCNQ3 | CNVR4057.1 | A | 2.57E-45 | 1.78E-41 | -0.1 | 1010.192 | 2.154 | 8 | 134397580 | 134399734 | 0 |  |  |  |
| PIP | IgH3.1246 | D | 1.58E-36 | 9.10E-33 | -0.525 | 159.257 | 0.209 | 7 | 142383973 | 142384182 | 0 |  |  |  |
| OR5B2 | Variation_38860 | A | 8.69E-35 | 4.18E-31 | -0.627 | 547.6695 | 4.436 | 11 | 57401386 | 57405822 | 0 |  |  |  |
| KRTAP1-3 | IgH3.2311 | D | 1.45E-34 | 6.26E-31 | -0.458 | 193.816 | 6.221 | 17 | 36641074 | 36647295 | 0 |  |  |  |
| VAV3 | Variation_48074 | A | 4.06E-31 | 1.45E-27 | -0.5 | 425.494 | 8.958 | 1 | 107691171 | 107700129 | 0 |  |  |  |
| CNTN6 | Variation_8404 | A | 4.83E-31 | 1.67E-27 | 0.8 | 55.1615 | 64.789 | 3 | 1242186 | 1306975 | 0 |  |  |  |
| LIPF | IgH3.1690 | A | 2.19E-28 | 6.88E-25 | -0.35 | 543.7395 | 0.09 | 10 | 90965097 | 90965187 | 0 |  |  |  |
| DGKB | IgH3.1130 | D | 5.12E-28 | 1.47E-24 | -0.362 | 1232.246 | 0.313 | 7 | 13282776 | 13283089 | 1 | 1 |  |  |
| C5orf40 | CNVR2662.1 | A | 2.31E-23 | 6.14E-20 | -0.673 | 519.6435 | 1.764 | 5 | 157223771 | 157225535 | 0 |  |  |  |
| UTS2D | CNVR1674.1 | A | 7.34E-22 | 1.81E-18 | -0.32 | 6.61 | 2.411 | 3 | 192493924 | 192496335 | 0 |  |  |  |
| TSHZ2 | CNVR7864.1 | D | 1.75E-19 | 4.03E-16 | -0.46 | 1195.462 | 2.265 | 20 | 50085498 | 50087763 | 0 |  |  |  |
| SNCG | CNVR4834.1 | A | 2.13E-18 | 4.45E-15 | -0.52 | 772.0485 | 7.459 | 10 | 87942313 | 87949772 | 0 |  |  |  |
| SLC35D3 | Variation_39477 | A | 2.74E-18 | 5.57E-15 | -0.241 | 648.1185 | 1.356 | 6 | 136639341 | 136640697 | 0 |  |  |  |
| OR9I1 | Variation_38860 | A | 5.11E-18 | 9.81E-15 | -0.531 | 243.8515 | 4.436 | 11 | 57401386 | 57405822 | 0 |  |  |  |
| ATOH1 | CNVR2005.1 | A | 4.59E-17 | 8.35E-14 | -0.131 | 886.7515 | 0.874 | 4 | 95856821 | 95857695 | 0 |  |  |  |
| FGF12 | CNVR1674.1 | A | 7.01E-16 | 1.21E-12 | -0.157 | 983.236 | 2.411 | 3 | 192493924 | 192496335 | 0 |  |  |  |
| ELTD1 | CNVR225.2 | D | 2.07E-15 | 3.41E-12 | -0.282 | 22.375 | 4.093 | 1 | 79166231 | 79170324 | 0 |  |  |  |
| WNT9B | CNVR7116.3 | A | 7.30E-15 | 1.15E-11 | -0.221 | 158.317 | 51.468 | 17 | 42480752 | 42532220 | 0 |  |  |  |
| KRTAP9-4 | IgH3.2311 | D | 2.40E-14 | 3.42E-11 | -0.436 | 21.984 | 6.221 | 17 | 36641074 | 36647295 | 0 |  |  |  |
| TMEM179 | IgH3.2106 | D | 2.59E-14 | 3.66E-11 | -0.251 | 273.5625 | 1297.4 | 14 | 105059071 | 106356482 | 0 |  |  |  |
| NRP1 | IgH3.1636 | D | 2.98E-14 | 4.12E-11 | -0.53 | 417.837 | 0.119 | 10 | 34003028 | 34003147 | 1 | 1 |  |  |
| HBM | IgH3.2190 | A | 1.49E-13 | 1.94E-10 | -0.483 | 11.8045 | 0.734 | 16 | 144932 | 145666 | 0 |  |  |  |
| IGFBP7 | CNVR1925.1 | A | 1.86E-13 | 2.38E-10 | 0.724 | 88.0655 | 50.489 | 4 | 57744958 | 57795447 | 0 |  |  |  |
| FAM163B | CNVR4504.1 | A | 1.00E-12 | 1.21E-09 | -0.281 | 319.054 | 5.282 | 9 | 135118152 | 135123434 | 0 |  |  |  |
| SERPINA6 | IgH3.2097 | D | 1.28E-12 | 1.53E-09 | 0.38 | 600.1055 | 1.759 | 14 | 94450874 | 94452633 | 1 | 1 |  |  |
| EYA2 | CNVR7858.1 | D | 2.51E-12 | 2.79E-09 | -0.684 | 879.164 | 1.78 | 20 | 44225509 | 44227289 | 0 |  |  |  |
| KRT39 | IgH3.2311 | D | 2.70E-12 | 2.98E-09 | -0.498 | 265.5315 | 6.221 | 17 | 36641074 | 36647295 | 0 |  |  |  |
| LHFPL3 | IgH3.1208 | A | 3.69E-12 | 3.86E-09 | -0.242 | 198.501 | 12.646 | 7 | 104251113 | 104263759 | 0 |  |  |  |
| LHFPL3 | CNVR3524.1 | A | 3.69E-12 | 3.86E-09 | -0.286 | 667.8475 | 2.847 | 7 | 104715560 | 104718407 | 0 |  |  |  |
| CYLC2 | IgH3.1558 | A | 6.48E-12 | 6.59E-09 | -0.535 | 799.206 | 0.594 | 9 | 105608505 | 105609099 | 0 |  |  |  |
| DRD1 | CNVR2688.1 | D | 1.03E-11 | 1.01E-08 | -0.625 | 435.697 | 4.559 | 5 | 174368607 | 174373166 | 0 |  |  |  |
| HTRA3 | CNVR1805.1 | D | 1.24E-11 | 1.19E-08 | -0.615 | 655.166 | 0.525 | 4 | 7686159 | 7686684 | 0 |  |  |  |
| LCE3C | IgH3.100 | A | 1.57E-11 | 1.46E-08 | -0.346 | 298.821 | 0.247 | 1 | 151138918 | 151139165 | 0 |  |  |  |
| HOXB6 | CNVR7121.1 | A | 1.77E-11 | 1.61E-08 | 0.513 | 28.5885 | 51.609 | 17 | 44087108 | 44138717 | 0 |  |  |  |
| CLCA2 | IgH3.55 | A | 6.31E-11 | 5.47E-08 | -0.382 | 256.037 | 2.36 | 1 | 86935809 | 86938169 | 1 |  |  |  |
| FRMD1 | CNVR3166.2 | A | 6.79E-11 | 5.85E-08 | 0.667 | 766.0495 | 172.42 | 6 | 167529509 | 167701933 | 0 |  |  |  |
| WISP2 | CNVR7855.1 | A | 1.11E-10 | 9.16E-08 | -0.352 | 852.5365 | 3.065 | 20 | 43637651 | 43640716 | 0 |  |  |  |
| FREM2 | CNVR5852.1 | A | 1.14E-10 | 9.38E-08 | -0.198 | 384.2355 | 4.124 | 13 | 38645517 | 38649641 | 0 |  |  |  |
| SNORD116-26 | IgH3.2113 | A | 2.96E-10 | 2.19E-07 | 0.352 | 1223.296 | 438.32 | 15 | 21891651 | 22329972 | 0 |  |  |  |
| JSRP1 | CNVR7475.1 | D | 3.42E-10 | 2.47E-07 | 0.531 | 791.568 | 5.657 | 19 | 1416058 | 1421715 | 0 |  |  |  |
| AVP | CNVR7768.1 | A | 3.63E-10 | 2.59E-07 | -0.542 | 294.655 | 0.679 | 20 | 3307280 | 3307959 | 0 |  |  |  |
| CFD | CNVR7475.1 | A | 3.72E-10 | 2.64E-07 | 0.63 | 600.5925 | 5.657 | 19 | 1416058 | 1421715 | 0 |  |  |  |
| DPPA5 | IgH3.1013 | A | 3.77E-10 | 2.67E-07 | -0.454 | 841.9165 | 0.752 | 6 | 73278550 | 73279302 | 1 | 1 |  |  |
| TAAR8 | IgH3.1053 | D | 4.44E-10 | 3.03E-07 | -0.446 | 872.6565 | 0.186 | 6 | 133788788 | 133788974 | 0 |  |  |  |
| SNORD109B | IgH3.2113 | A | 4.44E-10 | 3.03E-07 | 0.353 | 1165.756 | 438.32 | 15 | 21891651 | 22329972 | 0 |  |  |  |
| SNORD109B | IgH3.2114 | A | 4.44E-10 | 3.03E-07 | 0.284 | 35.7105 | 2.554 | 15 | 22875234 | 22877788 | 0 |  |  |  |
| SNORD116-29 | IgH3.2113 | A | 4.98E-10 | 3.31E-07 | 0.354 | 1230.311 | 438.32 | 15 | 21891651 | 22329972 | 0 |  |  |  |
| SNORD116-29 | IgH3.2114 | A | 4.98E-10 | 3.31E-07 | 0.279 | 28.8445 | 2.554 | 15 | 22875234 | 22877788 | 0 |  |  |  |
| GINS2 | IgH3.2260 | A | 5.65E-10 | 3.68E-07 | 0.668 | 627.387 | 2.23 | 16 | 83648163 | 83650393 | 1 | 1 |  |  |
| MYH13 | IgH3.2284 | D | 7.15E-10 | 4.44E-07 | -0.331 | 620.191 | 1.296 | 17 | 10801816 | 10803112 | 1 | 1 |  |  |
| SNORD116-26 | IgH3.2114 | A | 7.21E-10 | 4.47E-07 | 0.292 | 21.829 | 2.554 | 15 | 22875234 | 22877788 | 0 |  |  |  |
| C21orf45 | CNVR7993.1 | D | 7.36E-10 | 4.54E-07 | -0.618 | 89.872 | 0.775 | 21 | 32478339 | 32479114 | 0 |  |  |  |
| RFXANK | Variation_35615 | A | 1.06E-09 | 6.31E-07 | -0.373 | 501.1065 | 40.148 | 19 | 19690023 | 19730171 | 0 |  |  |  |
| AHSA1 | IgH3.2077 | A | 1.23E-09 | 7.19E-07 | -0.673 | 352.0225 | 0.2 | 14 | 76647920 | 76648120 | 0 |  |  |  |
| EML4 | CNVR765.1 | D | 1.34E-09 | 7.75E-07 | -0.542 | 514.145 | 0.883 | 2 | 41817888 | 41818771 | 0 |  |  |  |
| ATP1B2 | Variation_35517 | D | 1.85E-09 | 1.02E-06 | -0.294 | 750.6985 | 5.201 | 17 | 8251695 | 8256896 | 0 |  |  |  |
| IGFBP3 | IgH3.1159 | A | 1.89E-09 | 1.03E-06 | -0.333 | 399.297 | 0.346 | 7 | 46322352 | 46322698 | 1 | 0 |  |  |
| FFAR3 | IgH3.2456 | D | 2.03E-09 | 1.09E-06 | -0.319 | 196.354 | 6.632 | 19 | 40349239 | 40355871 | 0 |  |  |  |
| SNORD116-11 | IgH3.2113 | A | 2.08E-09 | 1.12E-06 | 0.339 | 1199.724 | 438.32 | 15 | 21891651 | 22329972 | 0 |  |  |  |
| SNORD116-11 | IgH3.2114 | A | 2.08E-09 | 1.12E-06 | 0.294 | 1.743 | 2.554 | 15 | 22875234 | 22877788 | 0 |  |  |  |
| HADH | IgH3.707 | A | 2.23E-09 | 1.18E-06 | -0.652 | 75.9995 | 0.041 | 4 | 109229104 | 109229145 | 1 |  |  |  |
| GSTT1 | IgH3.2579 | D | 2.46E-09 | 1.27E-06 | 0.637 | 173.772 | 130.59 | 22 | 22601733 | 22732321 | 0 |  | CEU|CHB|JPT|YRI | |
| C9orf142 | Variation_57995 | A | 2.50E-09 | 1.29E-06 | -0.399 | 883.8865 | 3.95 | 9 | 139893331 | 139897281 | 0 |  |  |  |
| SIGLEC12 | IgH3.2468 | D | 2.68E-09 | 1.36E-06 | -0.577 | 120.837 | 23.669 | 19 | 56823613 | 56847282 | 0 |  |  |  |
| HULC | CNVR2770.1 | D | 3.13E-09 | 1.56E-06 | -0.465 | 267.5645 | 8.085 | 6 | 8869867 | 8877952 | 0 |  |  |  |
| C1orf54 | Variation_48133 | A | 3.32E-09 | 1.64E-06 | -0.376 | 418.828 | 3.732 | 1 | 148936576 | 148940308 | 0 |  |  |  |
| ALDH1L1 | IgH3.491 | D | 4.09E-09 | 1.99E-06 | -0.55 | 188.865 | 2.58 | 3 | 127156061 | 127158641 | 0 |  |  |  |
| ZNF835 | IgH3.2478 | A | 4.93E-09 | 2.36E-06 | -0.3 | 914.2355 | 0.601 | 19 | 60957476 | 60958077 | 0 |  |  |  |
| C6orf115 | IgH3.1061 | A | 5.86E-09 | 2.75E-06 | 0.574 | 897.866 | 0.631 | 6 | 138501271 | 138501902 | 1 | 0 |  |  |
| RPRM | IgH3.330 | D | 6.21E-09 | 2.90E-06 | 0.43 | 738.089 | 0.273 | 2 | 153304880 | 153305153 | 0 |  |  |  |
| EPHA2 | CNVR85.1 | A | 8.93E-09 | 3.97E-06 | 0.37 | 1012.987 | 4.865 | 1 | 15328730 | 15333595 | 0 |  |  |  |
| SOD1 | CNVR7993.1 | D | 1.04E-08 | 4.51E-06 | -0.584 | 519.492 | 0.775 | 21 | 32478339 | 32479114 | 0 |  |  |  |
| CD1E | IgH3.108 | A | 1.07E-08 | 4.62E-06 | -0.445 | 636.2075 | 0.757 | 1 | 157228651 | 157229408 | 0 |  |  |  |
| CD68 | Variation_35517 | D | 1.09E-08 | 4.69E-06 | -0.187 | 824.254 | 5.201 | 17 | 8251695 | 8256896 | 0 |  |  |  |
| ETV1 | IgH3.1130 | D | 1.11E-08 | 4.76E-06 | -0.278 | 662.4035 | 0.313 | 7 | 13282776 | 13283089 | 1 | 1 |  |  |
| DNASE2B | Variation_22534 | D | 1.26E-08 | 5.27E-06 | -0.588 | 163.6055 | 4.764 | 1 | 84483817 | 84488581 | 0 |  |  |  |
| C7orf65 | IgH3.1162 | A | 1.31E-08 | 5.43E-06 | -0.326 | 904.5525 | 3.184 | 7 | 48570713 | 48573897 | 1 | 1 |  |  |
| ASAP2 | CNVR672.1 | A | 1.31E-08 | 5.43E-06 | -0.369 | 697.4085 | 0.826 | 2 | 8666810 | 8667636 | 0 |  |  |  |
| C17orf66 | IgH3.2307 | D | 1.31E-08 | 5.43E-06 | 0.338 | 23.258 | 472.07 | 17 | 31425819 | 31897893 | 0 |  |  |  |
| PCDH8 | IgH3.1975 | D | 1.47E-08 | 5.94E-06 | -0.296 | 394.1835 | 89.765 | 13 | 51969141 | 52058906 | 0 |  |  |  |
| AGAP11 | CNVR4842.1 | A | 1.54E-08 | 6.15E-06 | 0.09 | 668.131 | 0.535 | 10 | 89408607 | 89409142 | 0 |  |  |  |
| SIGLEC5 | IgH3.2468 | D | 1.56E-08 | 6.21E-06 | -0.612 | 4.2875 | 23.669 | 19 | 56823613 | 56847282 | 0 |  |  | CEU|YRI |
| HAPLN3 | CNVR6504.1 | A | 1.70E-08 | 6.63E-06 | -0.355 | 618.889 | 44.776 | 15 | 87871925 | 87916701 | 0 |  | YRI | |
| GAL3ST1 | IgH3.2586 | A | 1.79E-08 | 6.88E-06 | -0.316 | 913.259 | 3.247 | 22 | 30200632 | 30203879 | 0 |  |  |  |
| FAM49A | IgH3.228 | A | 1.82E-08 | 6.97E-06 | -0.567 | 272.4665 | 0.664 | 2 | 16925212 | 16925876 | 0 |  |  |  |
| LIPF | IgH3.1689 | A | 1.90E-08 | 7.19E-06 | -0.354 | 508.071 | 7.127 | 10 | 90932947 | 90940074 | 0 |  |  |  |
| MRPL17 | Variation_48876 | D | 1.93E-08 | 7.27E-06 | -0.653 | 295.9385 | 3.888 | 11 | 6957582 | 6961470 | 0 |  |  |  |
| C1orf96 | IgH3.181 | D | 1.95E-08 | 7.32E-06 | 0.435 | 733.107 | 0.203 | 1 | 228267551 | 228267754 | 1 | 0 |  |  |
| COL27A1 | CNVR4450.1 | D | 2.02E-08 | 7.51E-06 | 0.279 | 704.2505 | 1.62 | 9 | 116740484 | 116742104 | 0 |  |  |  |
| YWHAE | Variation_39192 | A | 2.41E-08 | 8.75E-06 | 0.428 | 478.5285 | 34.808 | 17 | 761321 | 796129 | 0 |  |  |  |
| CDC26 | Variation_36907 | A | 2.60E-08 | 9.33E-06 | 0.53 | 765.241 | 1.089 | 9 | 114308704 | 114309793 | 0 |  |  |  |
| MEF2B | Variation_35615 | A | 2.69E-08 | 9.60E-06 | -0.39 | 540.212 | 40.148 | 19 | 19690023 | 19730171 | 0 |  |  |  |
| ASF1B | IgH3.2436 | A | 2.79E-08 | 9.90E-06 | 0.506 | 736.1325 | 2.181 | 19 | 14837103 | 14839284 | 1 | 1 |  |  |
| S1PR5 | CNVR7533.1 | A | 2.90E-08 | 1.02E-05 | -0.458 | 414.7165 | 2.239 | 19 | 10073175 | 10075414 | 0 |  |  |  |
| RELL2 | Variation_31250 | D | 3.14E-08 | 1.10E-05 | 0.16 | 760.3875 | 10.212 | 5 | 140243476 | 140253688 | 0 |  |  |  |
| MB | CNVR8144.1 | A | 4.61E-08 | 1.57E-05 | -0.623 | 718.023 | 3.999 | 22 | 33625028 | 33629027 | 0 |  |  |  |
| EIF3IP1 | IgH3.1216 | A | 4.81E-08 | 1.63E-05 | -0.297 | 67.457 | 0.629 | 7 | 109454784 | 109455413 | 1 | 1 |  |  |
| GPC6 | Variation_49277 | A | 5.86E-08 | 1.96E-05 | -0.098 | 1517.288 | 6.361 | 13 | 94788141 | 94794502 | 0 |  |  |  |
| C6orf115 | IgH3.1062 | D | 6.07E-08 | 2.02E-05 | -0.63 | 881.704 | 4.111 | 6 | 138519173 | 138523284 | 1 | 1 |  |  |
| BLMH | IgH3.2305 | A | 7.08E-08 | 2.32E-05 | -0.554 | 808.7175 | 0.025 | 17 | 26430004 | 26430029 | 1 |  |  |  |
| SNORA28 | CNVR6266.1 | D | 7.77E-08 | 2.52E-05 | 0.332 | 854.0515 | 0.525 | 14 | 102020212 | 102020737 | 0 |  |  |  |
| PSORS1C1 | IgH3.978 | D | 7.86E-08 | 2.55E-05 | -0.479 | 105.85 | 19.043 | 6 | 31318580 | 31337623 | 0 |  |  |  |
| FUT7 | Variation_57995 | A | 9.15E-08 | 2.91E-05 | -0.183 | 845.5765 | 3.95 | 9 | 139893331 | 139897281 | 0 |  |  |  |
| IFI27L1 | IgH3.2097 | A | 9.20E-08 | 2.92E-05 | -0.125 | 821.893 | 1.759 | 14 | 94450874 | 94452633 | 1 | 1 |  |  |
| UGT2B17 | IgH3.663 | D | 1.08E-07 | 3.35E-05 | 0.618 | 424.191 | 532.31 | 4 | 68943134 | 69475447 | 0 |  | CEU|CHB|JPT | CEU|CHB|JPT|YRI |
| FAM134B | CNVR2379.1 | A | 1.09E-07 | 3.37E-05 | -0.305 | 844.5295 | 14.26 | 5 | 17395748 | 17410008 | 0 |  |  |  |
| UBE2L3 | IgH3.2575 | A | 1.09E-07 | 3.37E-05 | 0.524 | 347.148 | 0.719 | 22 | 20627647 | 20628366 | 0 |  | JPT|YRI | |
| ODAM | IgH3.668 | A | 1.11E-07 | 3.42E-05 | -0.358 | 190.66 | 0.082 | 4 | 70910238 | 70910320 | 1 |  |  |  |
| TAP1 | Variation_32788 | D | 1.17E-07 | 3.58E-05 | -0.419 | 461.883 | 8.577 | 6 | 32467750 | 32476327 | 0 |  |  |  |
| ENPP3 | Variation_36458 | D | 1.37E-07 | 4.13E-05 | -0.213 | 687.9055 | 8.426 | 6 | 132747307 | 132755733 | 0 |  |  |  |
| CRYGB | IgH3.367 | A | 1.42E-07 | 4.27E-05 | -0.39 | 45.6875 | 0.308 | 2 | 208763173 | 208763481 | 1 | 0 |  |  |
| TSTA3 | CNVR4125.1 | A | 2.11E-07 | 6.05E-05 | 0.532 | 885.642 | 6.121 | 8 | 145657105 | 145663226 | 0 |  |  |  |
| ZNF667 | IgH3.2478 | A | 2.17E-07 | 6.20E-05 | -0.21 | 704.3675 | 0.601 | 19 | 60957476 | 60958077 | 0 |  |  |  |
| RHAG | CNVR2892.1 | D | 2.29E-07 | 6.49E-05 | -0.289 | 750.535 | 5.584 | 6 | 48948954 | 48954538 | 0 |  |  |  |
| C3orf55 | IgH3.524 | D | 2.31E-07 | 6.54E-05 | -0.261 | 93.866 | 0.374 | 3 | 158667931 | 158668305 | 0 |  |  |  |
| CKS2 | CNVR4389.1 | D | 2.32E-07 | 6.57E-05 | 0.584 | 643.304 | 2.134 | 9 | 90476448 | 90478582 | 0 |  |  |  |
| FARS2 | IgH3.961 | A | 2.42E-07 | 6.81E-05 | 0.555 | 234.2505 | 0.248 | 6 | 5227572 | 5227820 | 1 | 0 |  |  |
| OSTN | CNVR1674.1 | A | 2.45E-07 | 6.88E-05 | -0.34 | 60.909 | 2.411 | 3 | 192493924 | 192496335 | 0 |  |  |  |
| PRKG1 | CNVR4740.1 | A | 2.58E-07 | 7.18E-05 | -0.167 | 1151.081 | 8.185 | 10 | 54228288 | 54236473 | 0 |  |  |  |
| RRP8 | Variation_48876 | D | 2.65E-07 | 7.34E-05 | -0.615 | 376.081 | 3.888 | 11 | 6957582 | 6961470 | 0 |  |  |  |
| MMRN2 | CNVR4834.1 | A | 2.69E-07 | 7.44E-05 | -0.249 | 757.7585 | 7.459 | 10 | 87942313 | 87949772 | 0 |  |  |  |
| LCE3D | IgH3.100 | D | 3.09E-07 | 8.44E-05 | -0.317 | 319.751 | 0.247 | 1 | 151138918 | 151139165 | 0 |  |  |  |
| TFDP1 | CNVR6061.3 | D | 3.39E-07 | 9.18E-05 | 0.62 | 245.026 | 31.233 | 13 | 113576064 | 113607297 | 0 |  |  |  |
| HPVC1 | IgH3.1167 | A | 3.84E-07 | 0.0001 | -0.254 | 693.0935 | 0.057 | 7 | 53543944 | 53544001 | 0 |  |  |  |
| C9orf40 | Variation_52861 | A | 3.86E-07 | 0.0001 | 0.497 | 593.7695 | 2.693 | 9 | 76162047 | 76164740 | 0 |  |  |  |
| C14orf115 | IgH3.2075 | A | 4.01E-07 | 0.00011 | -0.198 | 418.4755 | 2.135 | 14 | 73473283 | 73475418 | 1 | 0 |  |  |
| PARK7 | IgH3.8 | D | 4.77E-07 | 0.00012 | 0.567 | 299.738 | 0.027 | 1 | 8255866 | 8255893 | 0 |  |  |  |
| PLSCR4 | IgH3.512 | D | 5.06E-07 | 0.00013 | -0.287 | 460.485 | 0.327 | 3 | 146961913 | 146962240 | 0 |  |  |  |
| SNORA21 | CNVR7083.3 | A | 5.20E-07 | 0.00013 | -0.331 | 719.463 | 82.281 | 17 | 33584385 | 33666666 | 0 |  |  |  |
| NUP35 | IgH3.351 | A | 5.79E-07 | 0.00015 | -0.394 | 782.715 | 7.88 | 2 | 184502645 | 184510525 | 0 |  |  |  |
| PLEKHF1 | IgH3.2454 | D | 5.86E-07 | 0.00015 | 0.381 | 906.437 | 0.161 | 19 | 35759712 | 35759873 | 1 | 1 |  |  |
| MRPL40 | IgH3.2570 | A | 6.90E-07 | 0.00017 | -0.201 | 817.543 | 288.31 | 22 | 17128428 | 17416739 | 0 |  |  |  |
| IDI2 | Variation_31707 | A | 7.28E-07 | 0.00018 | 0.446 | 897.735 | 2.575 | 10 | 161875 | 164450 | 0 |  |  |  |
| C1orf57 | CNVR541.1 | A | 7.39E-07 | 0.00018 | 0.134 | 214.535 | 8.988 | 1 | 230956876 | 230965864 | 0 |  |  |  |
| MEA1 | IgH3.990 | A | 7.46E-07 | 0.00018 | 0.439 | 57.7485 | 0.357 | 6 | 43146697 | 43147054 | 1 | 0 |  |  |
| SLC25A39 | IgH3.2319 | A | 7.50E-07 | 0.00019 | 0.478 | 829.3685 | 1.166 | 17 | 40585082 | 40586248 | 0 |  |  |  |
| RWDD2B | IgH3.2553 | A | 9.13E-07 | 0.00022 | 0.558 | 214.1055 | 5.047 | 21 | 29095172 | 29100219 | 1 | 0 |  |  |
| MPZL1 | CNVR396.1 | D | 1.02E-06 | 0.00024 | 0.616 | 844.8785 | 2.432 | 1 | 166838889 | 166841321 | 0 |  |  |  |
| THTPA | Variation_49328 | A | 1.09E-06 | 0.00026 | -0.271 | 763.972 | 5.596 | 14 | 23863602 | 23869198 | 0 |  |  |  |
| TET3 | IgH3.274 | D | 1.15E-06 | 0.00027 | 0.57 | 181.6485 | 11.268 | 2 | 73981869 | 73993137 | 0 |  |  |  |
| DDX4 | IgH3.870 | D | 1.15E-06 | 0.00027 | 0.407 | 583.298 | 0.712 | 5 | 55692636 | 55693348 | 0 |  |  |  |
| MRS2 | IgH3.972 | A | 1.15E-06 | 0.00027 | 0.394 | 533.2775 | 0.021 | 6 | 25055751 | 25055772 | 1 |  |  |  |
| IFI27L2 | IgH3.2097 | A | 1.18E-06 | 0.00027 | -0.075 | 785.2045 | 1.759 | 14 | 94450874 | 94452633 | 1 | 1 |  |  |
| RBKS | IgH3.234 | D | 1.22E-06 | 0.00028 | -0.392 | 617.535 | 0.186 | 2 | 27294806 | 27294992 | 1 | 1 |  |  |
| CTGF | Variation_36458 | D | 1.24E-06 | 0.00028 | -0.242 | 430.484 | 8.426 | 6 | 132747307 | 132755733 | 0 |  |  |  |
| INTS10 | IgH3.1396 | A | 1.35E-06 | 0.0003 | -0.465 | 311.725 | 0.023 | 8 | 20048268 | 20048291 | 0 |  |  |  |
| GINS2 | IgH3.2264 | A | 1.38E-06 | 0.00031 | -0.582 | 183.368 | 0.018 | 16 | 84457812 | 84457830 | 1 | 0 |  |  |
| HEY2 | IgH3.1047 | A | 1.48E-06 | 0.00033 | -0.262 | 894.368 | 4.53 | 6 | 125226163 | 125230693 | 1 | 1 |  |  |
| RFC5 | IgH3.1924 | A | 1.52E-06 | 0.00033 | 0.581 | 795.9005 | 7.297 | 12 | 117746205 | 117753502 | 0 |  |  |  |
| STYK1 | IgH3.1837 | A | 1.55E-06 | 0.00034 | -0.436 | 216.9365 | 9.011 | 12 | 10478050 | 10487061 | 0 |  |  |  |
| KIF26A | Variation_35202 | D | 1.55E-06 | 0.00034 | -0.422 | 328.026 | 9.218 | 14 | 104028535 | 104037753 | 0 |  |  |  |
| FRG2 | Variation_42881 | D | 1.62E-06 | 0.00035 | 0.252 | 977.614 | 0.57 | 4 | 190206632 | 190207202 | 0 |  |  |  |
| FGFRL1 | CNVR1731.1 | D | 1.71E-06 | 0.00037 | -0.507 | 463.4355 | 7.17 | 4 | 543618 | 550788 | 0 |  |  |  |
| CCT6A | CNVR3397.1 | A | 1.71E-06 | 0.00037 | 0.507 | 816.95 | 93.529 | 7 | 55322838 | 55416367 | 0 |  |  |  |
| CCT6A | CNVR3398.4 | A | 1.71E-06 | 0.00037 | 0.59 | 888.5355 | 765.61 | 7 | 55587294 | 56352906 | 0 |  |  |  |
| ILK | Variation_48876 | D | 1.82E-06 | 0.00038 | -0.561 | 370.531 | 3.888 | 11 | 6957582 | 6961470 | 0 |  |  |  |
| BDH1 | IgH3.568 | A | 1.84E-06 | 0.00039 | -0.574 | 954.234 | 0.535 | 3 | 197790186 | 197790721 | 0 |  |  |  |
| MFSD3 | CNVR4125.1 | A | 1.87E-06 | 0.00039 | 0.486 | 52.3335 | 6.121 | 8 | 145657105 | 145663226 | 0 |  |  |  |
| C11orf73 | IgH3.1781 | A | 1.88E-06 | 0.00039 | 0.55 | 172.9315 | 0.569 | 11 | 85885982 | 85886551 | 1 | 0 |  |  |
| ARL5A | IgH3.329 | A | 2.02E-06 | 0.00041 | -0.296 | 984.315 | 0.62 | 2 | 151395485 | 151396105 | 0 |  |  |  |
| C14orf1 | Variation_49412 | A | 2.04E-06 | 0.00042 | 0.533 | 455.03 | 8.194 | 14 | 74741204 | 74749398 | 0 |  |  |  |
| CLNK | Variation_44123 | A | 2.05E-06 | 0.00042 | -0.315 | 451.811 | 1.157 | 4 | 10650599 | 10651756 | 0 |  |  |  |
| YWHAE | IgH3.2276 | A | 2.07E-06 | 0.00042 | -0.514 | 842.123 | 1.333 | 17 | 2065235 | 2066568 | 1 | 0 |  |  |
| REEP1 | IgH3.289 | A | 2.08E-06 | 0.00042 | 0.473 | 195.366 | 0.084 | 2 | 86161135 | 86161219 | 0 |  |  |  |
| MAGEL2 | IgH3.2108 | A | 2.21E-06 | 0.00044 | -0.394 | 811.601 | 30.654 | 15 | 20645664 | 20676318 | 0 |  |  |  |
| TUBB4Q | Variation_42881 | D | 2.22E-06 | 0.00044 | 0.243 | 935.4975 | 0.57 | 4 | 190206632 | 190207202 | 0 |  |  |  |
| AKIRIN2 | IgH3.1024 | A | 2.24E-06 | 0.00045 | -0.511 | 1008.372 | 19.267 | 6 | 87456263 | 87475530 | 0 |  |  |  |
| OR8D1 | Variation_48712 | A | 2.25E-06 | 0.00045 | 0.295 | 267.6495 | 4.386 | 11 | 123419952 | 123424338 | 0 |  |  |  |
| S100A7 | IgH3.99 | A | 2.27E-06 | 0.00045 | -0.529 | 608.3625 | 7.037 | 1 | 151093458 | 151100495 | 0 |  |  |  |
| CRISP3 | Variation_37753 | A | 2.27E-06 | 0.00045 | 0.518 | 276.4025 | 15.73 | 6 | 49539524 | 49555254 | 0 |  |  |  |
| CPA3 | IgH3.515 | A | 2.35E-06 | 0.00046 | -0.323 | 359.4045 | 9.239 | 3 | 150445670 | 150454909 | 0 |  |  |  |
| TCTEX1D2 | IgH3.567 | A | 2.50E-06 | 0.00049 | -0.569 | 255.1085 | 0.147 | 3 | 197260987 | 197261134 | 0 |  |  |  |
| HHEX | Variation_38216 | D | 2.51E-06 | 0.00049 | -0.286 | 292.7445 | 7.365 | 10 | 94153462 | 94160827 | 0 |  |  |  |
| AP2A2 | IgH3.1714 | D | 2.55E-06 | 0.0005 | -0.459 | 639.1105 | 8.783 | 11 | 1602542 | 1611325 | 0 |  |  |  |
| NSMCE1 | CNVR6680.1 | A | 2.63E-06 | 0.00051 | 0.414 | 183.231 | 0.625 | 16 | 26982796 | 26983421 | 0 |  |  |  |
| BRD2 | IgH3.982 | D | 2.81E-06 | 0.00054 | -0.434 | 890.2925 | 26.427 | 6 | 32173758 | 32200185 | 0 |  |  |  |
| HADH | CNVR2038.1 | A | 2.97E-06 | 0.00056 | 0.586 | 782.7805 | 0.859 | 4 | 109936294 | 109937153 | 0 |  |  |  |
| PPP4R4 | IgH3.2097 | A | 3.01E-06 | 0.00057 | -0.062 | 686.8815 | 1.759 | 14 | 94450874 | 94452633 | 1 | 1 |  |  |
| MRPL32 | IgH3.1156 | A | 3.14E-06 | 0.00059 | -0.544 | 836.145 | 0.609 | 7 | 42105380 | 42105989 | 1 | 0 |  |  |
| C22orf15 | IgH3.2576 | D | 3.17E-06 | 0.00059 | -0.462 | 2183.309 | 896.83 | 22 | 20701734 | 21598562 | 0 |  |  |  |
| ARPC4 | IgH3.407 | A | 3.40E-06 | 0.00063 | -0.594 | 885.52 | 0.287 | 3 | 8931131 | 8931418 | 1 | 0 |  |  |
| ADSS | IgH3.194 | A | 3.42E-06 | 0.00063 | -0.586 | 828.5605 | 1.005 | 1 | 241832169 | 241833174 | 1 | 1 | CEU | |
| PSMA7 | CNVR7909.1 | A | 3.43E-06 | 0.00063 | 0.539 | 602.3725 | 0.595 | 20 | 59546452 | 59547047 | 0 |  |  |  |
| TCTEX1D2 | IgH3.568 | A | 3.51E-06 | 0.00064 | -0.577 | 273.8965 | 0.535 | 3 | 197790186 | 197790721 | 0 |  |  |  |
| SOHLH1 | IgH3.1586 | D | 3.61E-06 | 0.00066 | -0.434 | 383.174 | 8.438 | 9 | 137349180 | 137357618 | 0 |  |  |  |
| RNF103 | IgH3.291 | A | 3.84E-06 | 0.00069 | -0.272 | 272.5885 | 1063.5 | 2 | 86953419 | 88016919 | 0 |  |  |  |
| METTL11A | Variation_57977 | A | 3.85E-06 | 0.00069 | -0.311 | 943.8525 | 9.416 | 9 | 132381538 | 132390954 | 0 |  |  |  |
| GPATCH8 | IgH3.2319 | A | 3.86E-06 | 0.00069 | -0.563 | 702.2475 | 1.166 | 17 | 40585082 | 40586248 | 0 |  |  |  |
| OR13C4 | IgH3.1558 | A | 4.19E-06 | 0.00074 | -0.23 | 720.6245 | 0.594 | 9 | 105608505 | 105609099 | 0 |  |  |  |
| OR13C9 | IgH3.1558 | A | 4.19E-06 | 0.00074 | -0.23 | 811.6195 | 0.594 | 9 | 105608505 | 105609099 | 0 |  |  |  |
| CNTN6 | IgH3.394 | A | 4.27E-06 | 0.00075 | -0.496 | 1062.377 | 4.867 | 3 | 205010 | 209877 | 0 |  |  |  |
| FDFT1 | IgH3.1372 | D | 4.46E-06 | 0.00078 | -0.536 | 671.5475 | 6.326 | 8 | 11047528 | 11053854 | 1 | 1 |  |  |
| GADD45B | Variation_40252 | D | 4.47E-06 | 0.00078 | 0.533 | 561.2865 | 0.452 | 19 | 1867135 | 1867587 | 0 |  |  |  |
| UBE2L3 | IgH3.2574 | A | 4.61E-06 | 0.0008 | -0.561 | 489.297 | 2.781 | 22 | 19792233 | 19795014 | 0 |  | JPT|YRI | |
| HCP5 | Variation_32788 | D | 4.76E-06 | 0.00082 | -0.384 | 923.2125 | 8.577 | 6 | 32467750 | 32476327 | 0 |  |  |  |
| FZD2 | CNVR7107.1 | D | 4.93E-06 | 0.00085 | 0.056 | 778.8525 | 8.328 | 17 | 39216753 | 39225081 | 0 |  |  |  |
| BARHL1 | CNVR4504.1 | A | 4.96E-06 | 0.00085 | -0.234 | 663.874 | 5.282 | 9 | 135118152 | 135123434 | 0 |  |  |  |
| MT1X | IgH3.2228 | D | 5.02E-06 | 0.00086 | -0.405 | 933.6575 | 25.58 | 16 | 54353878 | 54379458 | 0 |  |  |  |
| NUDC | IgH3.25 | A | 5.12E-06 | 0.00087 | 0.466 | 269.341 | 15.972 | 1 | 26871787 | 26887759 | 1 | 1 |  |  |
| TGFB1 | CNVR7646.1 | A | 5.30E-06 | 0.00089 | -0.263 | 159.204 | 5.567 | 19 | 46383741 | 46389308 | 0 |  |  |  |
| CDH7 | IgH3.2391 | A | 5.37E-06 | 0.0009 | 0.446 | 286.451 | 7.346 | 18 | 61351033 | 61358379 | 0 |  |  |  |
| RGS2 | IgH3.145 | A | 5.45E-06 | 0.00091 | 0.06 | 375.295 | 0.836 | 1 | 191422123 | 191422959 | 1 | 1 |  |  |
| SNORD93 | IgH3.1141 | D | 5.58E-06 | 0.00093 | -0.382 | 926.228 | 6.31 | 7 | 21939720 | 21946030 | 1 | 1 |  |  |
| ILF2 | Variation_43741 | A | 5.69E-06 | 0.00095 | 0.568 | 440.0225 | 3.779 | 1 | 152347532 | 152351311 | 0 |  |  |  |
| SLC29A1 | IgH3.992 | D | 5.96E-06 | 0.00098 | -0.508 | 766.805 | 0.677 | 6 | 45071713 | 45072390 | 0 |  |  |  |
| HNRNPUL1 | CNVR7646.1 | A | 6.22E-06 | 0.00102 | -0.192 | 101.983 | 5.567 | 19 | 46383741 | 46389308 | 0 |  |  |  |
| EMX2OS | Variation_39063 | D | 6.38E-06 | 0.00104 | 0.414 | 256.2355 | 15.289 | 10 | 119015590 | 119030879 | 0 |  |  |  |
| KLHDC3 | IgH3.990 | A | 6.70E-06 | 0.00108 | 0.41 | 53.037 | 0.357 | 6 | 43146697 | 43147054 | 1 | 0 |  |  |
| HEATR7B2 | Variation_38608 | A | 6.90E-06 | 0.00111 | -0.33 | 707.343 | 16.071 | 5 | 40371232 | 40387303 | 0 |  |  |  |
| PSTK | Variation_33232 | A | 6.96E-06 | 0.00112 | -0.311 | 448.0285 | 0.654 | 10 | 125183075 | 125183729 | 0 |  |  |  |
| ORC4L | Variation_23173 | A | 7.03E-06 | 0.00113 | -0.348 | 799.726 | 8.958 | 2 | 147656248 | 147665206 | 0 |  |  |  |
| C1orf31 | CNVR543.1 | D | 7.24E-06 | 0.00115 | -0.625 | 748.7345 | 0.78 | 1 | 231832888 | 231833668 | 0 |  |  |  |
| CTSB | IgH3.1372 | A | 7.68E-06 | 0.00121 | 0.564 | 705.8835 | 6.326 | 8 | 11047528 | 11053854 | 1 | 1 |  |  |
| APC2 | CNVR7483.1 | A | 7.68E-06 | 0.00121 | -0.544 | 880.605 | 5.94 | 19 | 2296270 | 2302210 | 0 |  |  |  |
| PDC | Variation_43748 | D | 7.70E-06 | 0.00121 | -0.294 | 638.5515 | 1.582 | 1 | 184050339 | 184051921 | 0 |  |  |  |
| RBPJL | CNVR7855.1 | A | 7.80E-06 | 0.00123 | -0.226 | 261.7275 | 3.065 | 20 | 43637651 | 43640716 | 0 |  |  |  |
| C3orf1 | IgH3.488 | A | 8.07E-06 | 0.00126 | 0.529 | 609.2995 | 0.647 | 3 | 121322559 | 121323206 | 1 | 0 |  |  |
| C6orf221 | IgH3.1015 | A | 8.61E-06 | 0.00133 | 0.099 | 632.8915 | 7.312 | 6 | 74766417 | 74773729 | 0 |  |  |  |
| CCL24 | IgH3.1186 | D | 8.80E-06 | 0.00135 | 0.552 | 369.5925 | 526.61 | 7 | 75912904 | 76439509 | 0 |  |  |  |
| DIRAS1 | Variation_40252 | A | 8.85E-06 | 0.00136 | -0.002 | 802.068 | 0.452 | 19 | 1867135 | 1867587 | 0 |  |  |  |
| COPE | Variation_35615 | A | 8.95E-06 | 0.00137 | -0.394 | 788.6885 | 40.148 | 19 | 19690023 | 19730171 | 0 |  |  |  |
| ZWINT | CNVR4755.1 | A | 9.14E-06 | 0.0014 | 0.497 | 484.5235 | 5.131 | 10 | 57307164 | 57312295 | 0 |  |  |  |
| ANKRD11 | CNVR6874.2 | A | 9.44E-06 | 0.00143 | -0.558 | 384.424 | 9.905 | 16 | 87593531 | 87603436 | 0 |  |  |  |
| SNORD68 | CNVR6865.1 | D | 9.48E-06 | 0.00144 | -0.295 | 990.68 | 3.26 | 16 | 87166324 | 87169584 | 0 |  |  |  |
| MAP3K14 | IgH3.2319 | A | 9.83E-06 | 0.00148 | -0.565 | 138.7345 | 1.166 | 17 | 40585082 | 40586248 | 0 |  |  |  |
| MRPS27 | IgH3.880 | A | 1.08E-05 | 0.0016 | 0.551 | 593.161 | 1.065 | 5 | 71008787 | 71009852 | 1 | 1 |  |  |
| OR8B4 | Variation_48712 | D | 1.11E-05 | 0.00163 | 0.24 | 381.753 | 4.386 | 11 | 123419952 | 123424338 | 0 |  |  |  |
| DCLRE1B | IgH3.82 | A | 1.17E-05 | 0.0017 | -0.506 | 929.681 | 0.205 | 1 | 115183672 | 115183877 | 0 |  | CEU|CHB|JPT | |
| DTX2 | IgH3.1186 | D | 1.19E-05 | 0.00173 | 0.479 | 301.476 | 526.61 | 7 | 75912904 | 76439509 | 0 |  |  |  |
| FAM164C | Variation_49412 | D | 1.21E-05 | 0.00175 | 0.435 | 126.8055 | 8.194 | 14 | 74741204 | 74749398 | 0 |  |  |  |
| CBX1 | CNVR7118.2 | A | 1.23E-05 | 0.00177 | -0.512 | 574.258 | 54.963 | 17 | 42971209 | 43026172 | 0 |  |  |  |
| GSTM1 | IgH3.75 | D | 1.24E-05 | 0.00178 | 0.671 | 29.0895 | 32.552 | 1 | 110022101 | 110054653 | 0 |  | CEU|CHB|JPT|YRI | |
| SNORD117 | IgH3.982 | D | 1.27E-05 | 0.00182 | 0.215 | 548.3775 | 26.427 | 6 | 32173758 | 32200185 | 0 |  |  |  |
| FKBP4 | CNVR5393.1 | A | 1.28E-05 | 0.00183 | 0.528 | 749.942 | 3.17 | 12 | 2031250 | 2034420 | 0 |  |  |  |
| TNFAIP8L2 | IgH3.95 | A | 1.35E-05 | 0.00191 | 0.52 | 184.443 | 4.257 | 1 | 149583860 | 149588117 | 0 |  |  |  |
| LCE3E | IgH3.100 | D | 1.36E-05 | 0.00192 | -0.183 | 333.482 | 0.247 | 1 | 151138918 | 151139165 | 0 |  |  |  |
| SF3B14 | Variation_50455 | A | 1.39E-05 | 0.00195 | 0.453 | 856.955 | 4.295 | 2 | 25007490 | 25011785 | 0 |  |  |  |
| SPTBN1 | IgH3.264 | A | 1.40E-05 | 0.00196 | 0.473 | 790.0145 | 0.274 | 2 | 55481143 | 55481417 | 1 | 0 |  |  |
| DUSP19 | IgH3.351 | A | 1.41E-05 | 0.00197 | -0.485 | 836.457 | 7.88 | 2 | 184502645 | 184510525 | 0 |  |  |  |
| APOA1BP | IgH3.104 | A | 1.46E-05 | 0.00202 | -0.532 | 384.689 | 22.842 | 1 | 155225558 | 155248400 | 1 | 1 | YRI | |
| MFAP2 | IgH3.20 | A | 1.47E-05 | 0.00203 | -0.41 | 927.724 | 0.597 | 1 | 18105149 | 18105746 | 1 | 1 |  |  |
| ZNF493 | IgH3.2442 | D | 1.47E-05 | 0.00203 | -0.502 | 444.394 | 0.843 | 19 | 20933627 | 20934470 | 1 | 1 | CEU|YRI | |
| BAT5 | Variation_32788 | A | 1.48E-05 | 0.00204 | -0.468 | 692.571 | 8.577 | 6 | 32467750 | 32476327 | 0 |  |  |  |
| MFGE8 | CNVR6504.1 | A | 1.48E-05 | 0.00204 | -0.313 | 599.245 | 44.776 | 15 | 87871925 | 87916701 | 0 |  |  |  |
| C12orf11 | Variation_49038 | D | 1.50E-05 | 0.00207 | -0.429 | 950.8 | 6.695 | 12 | 26018497 | 26025192 | 0 |  |  |  |
| C9orf139 | Variation_57995 | A | 1.54E-05 | 0.00211 | -0.118 | 844.9605 | 3.95 | 9 | 139893331 | 139897281 | 0 |  |  |  |
| TRIML1 | IgH3.804 | D | 1.61E-05 | 0.00218 | 0.386 | 868.152 | 22.82 | 4 | 188444875 | 188467695 | 0 |  |  |  |
| TRIML1 | IgH3.806 | D | 1.61E-05 | 0.00218 | 0.26 | 88.0545 | 46.973 | 4 | 189413158 | 189460131 | 0 |  |  |  |
| TRIML1 | Variation_36253 | D | 1.61E-05 | 0.00218 | 0.309 | 254.1045 | 45.849 | 4 | 189578646 | 189624495 | 0 |  |  |  |
| IGSF10 | CNVR1586.1 | D | 1.62E-05 | 0.00219 | -0.539 | 326.247 | 39.511 | 3 | 152994179 | 153033690 | 0 |  |  |  |
| PPME1 | Variation_48880 | A | 1.62E-05 | 0.00219 | -0.213 | 244.9935 | 10.46 | 11 | 73361942 | 73372402 | 0 |  |  |  |
| SLFN12L | IgH3.2307 | A | 1.62E-05 | 0.00219 | 0.2 | 332.2665 | 472.07 | 17 | 31425819 | 31897893 | 0 |  |  |  |
| NFIL3 | IgH3.1550 | D | 1.68E-05 | 0.00225 | 0.478 | 189.0845 | 2.145 | 9 | 93408713 | 93410858 | 1 | 1 |  |  |
| ZNF318 | IgH3.990 | A | 1.72E-05 | 0.0023 | -0.538 | 281.9535 | 0.357 | 6 | 43146697 | 43147054 | 1 | 0 |  |  |
| FKBP3 | IgH3.2061 | D | 1.83E-05 | 0.00242 | -0.486 | 434.979 | 0.558 | 14 | 45099275 | 45099833 | 0 |  |  |  |
| GRHL3 | Variation_38985 | A | 1.90E-05 | 0.00249 | -0.144 | 156.5405 | 11.454 | 1 | 24387442 | 24398896 | 0 |  |  |  |
| SCFD2 | Variation_51414 | A | 1.92E-05 | 0.00251 | 0.538 | 341.19 | 9.64 | 4 | 54026463 | 54036103 | 0 |  |  |  |
| TCF19 | IgH3.982 | A | 1.93E-05 | 0.00252 | 0.52 | 923.4185 | 26.427 | 6 | 32173758 | 32200185 | 0 |  |  |  |
| MMP11 | IgH3.2576 | D | 1.97E-05 | 0.00256 | -0.354 | 2197.449 | 896.83 | 22 | 20701734 | 21598562 | 0 |  |  |  |
| FKBP4 | Variation_49056 | D | 2.01E-05 | 0.0026 | 0.449 | 443.33 | 2.372 | 12 | 3224123 | 3226495 | 0 |  |  |  |
| CSRP2BP | IgH3.2501 | D | 2.02E-05 | 0.00261 | 0.487 | 119.271 | 4.94 | 20 | 18215689 | 18220629 | 0 |  |  |  |
| PRMT3 | IgH3.1731 | D | 2.05E-05 | 0.00265 | -0.5 | 275.9695 | 0.051 | 11 | 20150608 | 20150659 | 1 |  |  |  |
| SF3B3 | IgH3.2236 | D | 2.05E-05 | 0.00265 | -0.471 | 564.9655 | 0.043 | 16 | 69707118 | 69707161 | 0 |  |  |  |
| C7orf28B | IgH3.1113 | D | 2.07E-05 | 0.00267 | -0.128 | 732.1145 | 10.537 | 7 | 6091895 | 6102432 | 0 |  |  |  |
| C7orf34 | IgH3.1242 | D | 2.12E-05 | 0.00272 | -0.25 | 601.969 | 1.431 | 7 | 141746148 | 141747579 | 0 |  |  |  |
| PROSC | IgH3.1406 | D | 2.14E-05 | 0.00274 | -0.514 | 319.6005 | 0.29 | 8 | 37428395 | 37428685 | 1 | 1 |  |  |
| UQCC | Variation_50650 | A | 2.25E-05 | 0.00285 | 0.49 | 195.6215 | 8.036 | 20 | 33608160 | 33616196 | 0 |  |  |  |
| MRPL1 | IgH3.683 | D | 2.32E-05 | 0.00291 | -0.465 | 436.659 | 0.046 | 4 | 79484580 | 79484626 | 0 |  |  |  |
| C20orf30 | Variation_56279 | D | 2.33E-05 | 0.00292 | 0.482 | 937.1825 | 2.309 | 20 | 5973445 | 5975754 | 0 |  |  |  |
| CDC45L | IgH3.2572 | D | 2.41E-05 | 0.003 | 0.362 | 293.895 | 0.306 | 22 | 18161823 | 18162129 | 0 |  |  |  |
| MRPL37 | CNVR185.1 | D | 2.42E-05 | 0.00301 | 0.44 | 263.016 | 0.713 | 1 | 54184876 | 54185589 | 0 |  |  |  |
| C1QTNF2 | CNVR2663.1 | D | 2.46E-05 | 0.00305 | -0.46 | 892.369 | 3.142 | 5 | 158827991 | 158831133 | 0 |  |  |  |
| MAS1L | Variation_57298 | D | 2.46E-05 | 0.00305 | -0.6 | 558.322 | 0.917 | 6 | 29005226 | 29006143 | 0 |  |  |  |
| CSH2 | CNVR7165.1 | A | 2.65E-05 | 0.00322 | -0.19 | 910.907 | 97.754 | 17 | 60263746 | 60361500 | 0 |  |  |  |
| KATNAL2 | IgH3.2372 | A | 2.72E-05 | 0.00328 | 0.43 | 976.0455 | 0.508 | 18 | 43807997 | 43808505 | 1 | 0 |  |  |
| C22orf29 | IgH3.2572 | D | 2.72E-05 | 0.00328 | 0.533 | 56.3455 | 0.306 | 22 | 18161823 | 18162129 | 0 |  |  |  |
| OSBPL2 | CNVR7909.1 | A | 2.74E-05 | 0.0033 | -0.415 | 729.6645 | 0.595 | 20 | 59546452 | 59547047 | 0 |  |  |  |
| EIF2AK3 | IgH3.291 | A | 2.77E-05 | 0.00333 | -0.272 | 2251.123 | 1063.5 | 2 | 86953419 | 88016919 | 0 |  |  |  |
| ZWINT | IgH3.1657 | A | 2.81E-05 | 0.00336 | 0.607 | 699.528 | 21.378 | 10 | 57100283 | 57121661 | 0 |  |  |  |
| APOBEC3B | IgH3.2594 | A | 2.81E-05 | 0.00336 | 0.494 | 56.3005 | 45.468 | 22 | 37679973 | 37725441 | 0 |  | CHB|JPT | JPT |
| TH1L | IgH3.2518 | A | 2.82E-05 | 0.00337 | -0.565 | 794.5465 | 0.441 | 20 | 56202318 | 56202759 | 1 | 0 |  |  |
| RPS6KB2 | CNVR5211.1 | A | 2.82E-05 | 0.00337 | 0.406 | 234.2 | 3.275 | 11 | 67191820 | 67195095 | 0 |  | CEU|CHB|JPT|YRI | |
| GLRX3 | IgH3.1707 | A | 2.83E-05 | 0.00338 | 0.406 | 35.8355 | 0.177 | 10 | 131882180 | 131882357 | 0 |  |  |  |
| GINS2 | IgH3.2262 | D | 2.86E-05 | 0.00341 | 0.559 | 533.135 | 1.406 | 16 | 83742003 | 83743409 | 1 | 1 |  |  |
| MRPL44 | IgH3.378 | A | 2.89E-05 | 0.00343 | 0.421 | 97.2505 | 2.636 | 2 | 224634088 | 224636724 | 1 | 0 |  |  |
| GCG | IgH3.337 | D | 2.91E-05 | 0.00345 | -0.324 | 743.4805 | 0.593 | 2 | 163456095 | 163456688 | 1 | 0 |  |  |
| C9orf140 | Variation_57995 | A | 2.91E-05 | 0.00345 | -0.213 | 810.732 | 3.95 | 9 | 139893331 | 139897281 | 0 |  |  |  |
| LYRM4 | IgH3.961 | A | 2.97E-05 | 0.0035 | 0.452 | 97.5365 | 0.248 | 6 | 5227572 | 5227820 | 1 | 0 |  |  |
| RDM1 | IgH3.2307 | A | 2.97E-05 | 0.0035 | 0.352 | 85.5255 | 472.07 | 17 | 31425819 | 31897893 | 0 |  |  |  |
| PPP1R15A | IgH3.2463 | D | 3.06E-05 | 0.00358 | 0.463 | 628.2965 | 0.082 | 19 | 53441040 | 53441122 | 0 |  |  |  |
| TARBP2 | CNVR5535.1 | A | 3.11E-05 | 0.00363 | 0.354 | 932.798 | 0.983 | 12 | 51251420 | 51252403 | 0 |  |  |  |
| C1orf158 | IgH3.14 | D | 3.16E-05 | 0.00367 | -0.244 | 145.579 | 343.02 | 1 | 12762151 | 13105174 | 0 |  |  |  |
| SIGLEC6 | Variation_50197 | A | 3.17E-05 | 0.00368 | -0.163 | 700.695 | 5.407 | 19 | 56022744 | 56028151 | 0 |  |  |  |
| TMEM126B | IgH3.1781 | A | 3.24E-05 | 0.00374 | 0.482 | 864.4505 | 0.569 | 11 | 85885982 | 85886551 | 1 | 0 |  |  |
| GSPT1 | CNVR6641.4 | A | 3.31E-05 | 0.0038 | -0.419 | 683.5995 | 3.07 | 16 | 12578540 | 12581610 | 0 |  |  |  |
| DDTL | CNVR8108.1 | A | 3.31E-05 | 0.0038 | -0.378 | 541.7145 | 2.662 | 22 | 22101503 | 22104165 | 0 |  |  |  |
| PRKAR1B | IgH3.1101 | A | 3.34E-05 | 0.00382 | 0.454 | 214.0175 | 0.379 | 7 | 851230 | 851609 | 1 | 1 |  |  |
| GINS2 | IgH3.2266 | D | 3.39E-05 | 0.00387 | -0.608 | 676.301 | 1.256 | 16 | 84951364 | 84952620 | 1 | 0 |  |  |
| ASF1A | IgH3.1044 | A | 3.41E-05 | 0.00388 | -0.474 | 275.633 | 0.971 | 6 | 118992683 | 118993654 | 1 | 1 |  |  |
| UTS2D | CNVR1678.1 | A | 3.43E-05 | 0.0039 | -0.249 | 873.0065 | 1.068 | 3 | 193372869 | 193373937 | 0 |  |  |  |
| KRTAP4-5 | Variation_49857 | D | 3.51E-05 | 0.00397 | 0.258 | 423.916 | 4.067 | 17 | 36137258 | 36141325 | 0 |  |  |  |
| PSMB8 | IgH3.982 | A | 3.54E-05 | 0.004 | 0.453 | 757.588 | 26.427 | 6 | 32173758 | 32200185 | 0 |  |  |  |
| TMEM91 | CNVR7646.1 | A | 3.65E-05 | 0.0041 | -0.311 | 197.0945 | 5.567 | 19 | 46383741 | 46389308 | 0 |  |  |  |
| CHORDC1 | Variation_31841 | D | 3.73E-05 | 0.00418 | -0.311 | 957.8185 | 6.602 | 11 | 90545833 | 90552435 | 0 |  |  |  |
| ATP5H | Variation_49925 | D | 3.76E-05 | 0.0042 | -0.337 | 209.0625 | 14.121 | 17 | 70348607 | 70362728 | 0 |  |  |  |
| NUDCD2 | IgH3.939 | A | 4.44E-05 | 0.00484 | 0.425 | 585.887 | 9.614 | 5 | 163407136 | 163416750 | 1 | 0 |  |  |
| ACN9 | IgH3.1199 | D | 4.45E-05 | 0.00485 | -0.063 | 473.489 | 4.119 | 7 | 97091974 | 97096093 | 0 |  | CHB|JPT|YRI | |
| OR4F6 | IgH3.2188 | D | 4.53E-05 | 0.00492 | -0.121 | 16.803 | 0.871 | 15 | 100181153 | 100182024 | 0 |  |  |  |
| OPN4 | CNVR4842.1 | A | 4.54E-05 | 0.00493 | 0.007 | 998.095 | 0.535 | 10 | 89408607 | 89409142 | 0 |  |  |  |
| IL4R | CNVR6680.1 | D | 4.57E-05 | 0.00496 | -0.473 | 275.692 | 0.625 | 16 | 26982796 | 26983421 | 0 |  |  |  |
| METTL13 | IgH3.118 | A | 4.62E-05 | 0.005 | 0.507 | 241.4455 | 0.343 | 1 | 170267048 | 170267391 | 1 | 1 |  |  |
| DDT | CNVR8108.1 | A | 4.71E-05 | 0.00508 | -0.385 | 547.614 | 2.662 | 22 | 22101503 | 22104165 | 0 |  |  |  |
| GOLSYN | Variation_52346 | D | 4.82E-05 | 0.00518 | -0.21 | 48.2365 | 10.683 | 8 | 110671493 | 110682176 | 0 |  |  |  |
| TIMM8B | CNVR5312.1 | A | 4.93E-05 | 0.00528 | 0.021 | 204.555 | 5.029 | 11 | 111668808 | 111673837 | 0 |  |  |  |
| VTCN1 | IgH3.83 | A | 4.94E-05 | 0.00528 | 0.223 | 725.2265 | 0 | 1 | 116796175 | 116796175 | 1 |  |  |  |
| HSP90AA1 | CNVR6266.1 | D | 5.03E-05 | 0.00536 | 0.393 | 399.9035 | 0.525 | 14 | 102020212 | 102020737 | 0 |  |  |  |
| SNHG5 | CNVR2988.1 | A | 5.04E-05 | 0.00537 | 0.353 | 718.902 | 5.019 | 6 | 87165718 | 87170737 | 0 |  |  |  |
| TMEM207 | IgH3.558 | D | 5.08E-05 | 0.00541 | -0.235 | 96.4835 | 0.911 | 3 | 191543721 | 191544632 | 1 | 1 |  |  |
| CMC1 | Variation_44105 | D | 5.08E-05 | 0.00541 | -0.302 | 69.2845 | 1.223 | 3 | 28367093 | 28368316 | 0 |  |  |  |
| TMEM207 | Variation_51043 | D | 5.08E-05 | 0.00541 | -0.144 | 624.676 | 3.152 | 3 | 192266001 | 192269153 | 0 |  |  |  |
| TET3 | Variation_30952 | A | 5.14E-05 | 0.00546 | -0.494 | 466.5925 | 1.788 | 2 | 73692185 | 73693973 | 0 |  |  |  |
| PARVA | Variation_43399 | A | 5.19E-05 | 0.0055 | -0.318 | 653.3675 | 1.95 | 11 | 11779461 | 11781411 | 0 |  |  |  |
| TTC23L | CNVR2429.1 | A | 5.40E-05 | 0.00569 | -0.096 | 800.2735 | 10.373 | 5 | 34110086 | 34120459 | 0 |  |  |  |
| EIF2B2 | Variation_49412 | A | 5.56E-05 | 0.00582 | 0.373 | 194.402 | 8.194 | 14 | 74741204 | 74749398 | 0 |  |  |  |
| MRPL14 | IgH3.992 | D | 5.73E-05 | 0.00597 | -0.519 | 875.1155 | 0.677 | 6 | 45071713 | 45072390 | 0 |  |  |  |
| CACYBP | IgH3.120 | D | 5.77E-05 | 0.006 | 0.394 | 171.4175 | 0.101 | 1 | 173070283 | 173070384 | 1 | 0 |  |  |
| HKR1 | CNVR7633.1 | D | 5.89E-05 | 0.00611 | -0.419 | 499.181 | 3.806 | 19 | 43033392 | 43037198 | 0 |  |  |  |
| ZNF85 | IgH3.2442 | D | 5.91E-05 | 0.00612 | -0.55 | 21.5745 | 0.843 | 19 | 20933627 | 20934470 | 1 | 1 | CEU|YRI | |
| PSMG3 | IgH3.1101 | D | 6.04E-05 | 0.00623 | 0.438 | 723.784 | 0.379 | 7 | 851230 | 851609 | 1 | 1 |  |  |
| CCDC50 | CNVR1674.1 | A | 6.06E-05 | 0.00625 | -0.177 | 71.641 | 2.411 | 3 | 192493924 | 192496335 | 0 |  |  |  |
| RPA1 | IgH3.2276 | A | 6.33E-05 | 0.00647 | -0.548 | 349.7585 | 1.333 | 17 | 2065235 | 2066568 | 1 | 0 |  |  |
| RB1 | IgH3.1973 | D | 6.51E-05 | 0.00662 | 0.328 | 68.8025 | 0.005 | 13 | 47796155 | 47796160 | 1 |  |  |  |
| TMOD3 | CNVR6390.1 | A | 6.62E-05 | 0.00671 | -0.256 | 99.3315 | 8.115 | 15 | 50052548 | 50060663 | 0 |  |  |  |
| MLX | IgH3.2314 | D | 6.63E-05 | 0.00672 | -0.542 | 988.7905 | 0.189 | 17 | 36986979 | 36987168 | 0 |  |  |  |
| KCNG3 | CNVR765.1 | A | 6.76E-05 | 0.00682 | -0.257 | 731.254 | 0.883 | 2 | 41817888 | 41818771 | 0 |  |  |  |
| DOCK1 | Variation_22563 | A | 6.80E-05 | 0.00685 | -0.427 | 287.6375 | 5.299 | 10 | 128577403 | 128582702 | 0 |  |  |  |
| PDLIM5 | CNVR2005.1 | A | 6.83E-05 | 0.00688 | -0.027 | 156.1545 | 0.874 | 4 | 95856821 | 95857695 | 0 |  |  |  |
| LMBR1 | IgH3.1276 | A | 6.84E-05 | 0.00688 | -0.258 | 189.152 | 2.389 | 7 | 156084539 | 156086928 | 0 |  |  |  |
| BCYRN1 | IgH3.2077 | A | 6.88E-05 | 0.00692 | -0.468 | 1115.365 | 0.2 | 14 | 76647920 | 76648120 | 0 |  |  |  |
| FUT5 | CNVR7515.1 | D | 6.97E-05 | 0.00699 | 0.306 | 968.022 | 33.719 | 19 | 6804075 | 6837794 | 0 |  |  |  |
| MLL4 | CNVR7629.1 | A | 7.00E-05 | 0.00701 | 0.503 | 515.409 | 45.309 | 19 | 41449253 | 41494562 | 0 |  |  |  |
| CCDC89 | IgH3.1779 | A | 7.28E-05 | 0.00724 | -0.344 | 826.8205 | 0.241 | 11 | 84247054 | 84247295 | 0 |  |  |  |
| RPRML | CNVR7116.3 | A | 7.50E-05 | 0.00742 | -0.288 | 43.951 | 51.468 | 17 | 42480752 | 42532220 | 0 |  |  |  |
| DDX39 | IgH3.2436 | A | 7.57E-05 | 0.00748 | 0.355 | 450.112 | 2.181 | 19 | 14837103 | 14839284 | 1 | 1 |  |  |
| ARTN | CNVR163.1 | D | 7.75E-05 | 0.00762 | -0.293 | 620.1555 | 0.727 | 1 | 44794057 | 44794784 | 0 |  |  |  |
| SSPO | IgH3.1256 | D | 7.96E-05 | 0.00779 | -0.212 | 406.4825 | 0.512 | 7 | 149539763 | 149540275 | 0 |  |  |  |
| MBP | IgH3.2407 | D | 8.01E-05 | 0.00783 | -0.427 | 490.245 | 0.092 | 18 | 72406570 | 72406662 | 1 | 1 |  |  |
| MCF2L | IgH3.2025 | A | 8.47E-05 | 0.00818 | -0.301 | 439.0305 | 18.317 | 13 | 113189448 | 113207765 | 0 |  |  |  |
| PNPO | CNVR7118.2 | A | 8.52E-05 | 0.00822 | -0.413 | 434.052 | 54.963 | 17 | 42971209 | 43026172 | 0 |  |  |  |
| PARD6A | CNVR6757.1 | D | 8.84E-05 | 0.00847 | -0.236 | 131.904 | 2.543 | 16 | 66122634 | 66125177 | 0 |  |  |  |
| MRPL52 | IgH3.2031 | A | 8.90E-05 | 0.00851 | -0.445 | 846.207 | 0.305 | 14 | 21525454 | 21525759 | 0 |  |  |  |
| JSRP1 | Variation_40252 | A | 9.06E-05 | 0.00863 | 0.132 | 337.8885 | 0.452 | 19 | 1867135 | 1867587 | 0 |  |  |  |
| SNORD41 | IgH3.2433 | A | 9.10E-05 | 0.00866 | -0.416 | 208.7495 | 0.873 | 19 | 12469984 | 12470857 | 0 |  |  |  |
| CCDC56 | IgH3.2315 | D | 9.23E-05 | 0.00876 | -0.421 | 124.9575 | 3.93 | 17 | 38330626 | 38334556 | 0 |  |  |  |
| HS3ST5 | Variation_46973 | A | 9.40E-05 | 0.00889 | -0.452 | 266.674 | 0.206 | 6 | 114753865 | 114754071 | 0 |  |  |  |
| TNFAIP3 | IgH3.1061 | A | 9.42E-05 | 0.0089 | -0.491 | 262.748 | 0.631 | 6 | 138501271 | 138501902 | 1 | 0 |  |  |
| PEBP1 | IgH3.1924 | D | 9.43E-05 | 0.00891 | 0.35 | 679.544 | 7.297 | 12 | 117746205 | 117753502 | 0 |  |  |  |
| TH1L | IgH3.2520 | D | 9.45E-05 | 0.00892 | -0.422 | 39.1915 | 0.947 | 20 | 56957926 | 56958873 | 1 | 0 |  |  |
| FAM98A | Variation_50462 | A | 9.49E-05 | 0.00895 | 0.516 | 147.2925 | 3.501 | 2 | 33524507 | 33528008 | 0 |  |  |  |
| NLRP6 | Variation_34903 | A | 9.54E-05 | 0.00899 | -0.352 | 728.0505 | 10.144 | 11 | 1005059 | 1015203 | 0 |  |  |  |
| HECW2 | CNVR1102.1 | D | 9.60E-05 | 0.00903 | -0.19 | 880.7435 | 1.112 | 2 | 197850200 | 197851312 | 0 |  |  |  |
| SUDS3 | IgH3.1926 | A | 9.65E-05 | 0.00907 | -0.439 | 830.2405 | 0.33 | 12 | 118149887 | 118150217 | 1 | 0 |  |  |
| MRPS14 | IgH3.120 | D | 9.70E-05 | 0.00911 | 0.415 | 184.2345 | 0.101 | 1 | 173070283 | 173070384 | 1 | 0 |  |  |
| LCN1 | IgH3.1588 | A | 9.78E-05 | 0.00916 | -0.417 | 995.313 | 0.397 | 9 | 138551164 | 138551561 | 0 |  |  |  |
| INSM2 | IgH3.2043 | D | 9.80E-05 | 0.00918 | -0.316 | 517.58 | 15.059 | 14 | 34564454 | 34579513 | 0 |  |  |  |
| KLHL22 | IgH3.2572 | D | 9.94E-05 | 0.00928 | 0.486 | 991.2935 | 0.306 | 22 | 18161823 | 18162129 | 0 |  |  |  |
| CCDC89 | IgH3.1782 | A | 0.0001 | 0.00954 | -0.472 | 909.5985 | 0.225 | 11 | 85983465 | 85983690 | 0 |  |  |  |
| ANO2 | Variation_39096 | A | 0.00011 | 0.00968 | -0.226 | 374.5105 | 6.641 | 12 | 6111699 | 6118340 | 0 |  |  |  |
| EEF1A1 | Variation_28363 | A | 0.00011 | 0.00975 | -0.129 | 72.11 | 0.325 | 6 | 74212887 | 74213212 | 0 |  |  |  |
| C14orf133 | IgH3.2077 | A | 0.00011 | 0.00975 | -0.474 | 330.3935 | 0.2 | 14 | 76647920 | 76648120 | 0 |  |  |  |
| OSGIN1 | IgH3.2259 | D | 0.00011 | 0.0099 | 0.49 | 106.574 | 5.745 | 16 | 82447181 | 82452926 | 0 |  |  |  |
| ADCK1 | IgH3.2077 | A | 0.00011 | 0.00997 | -0.448 | 755.2935 | 0.2 | 14 | 76647920 | 76648120 | 0 |  |  |  |
| LMTK2 | IgH3.1203 | A | 0.00011 | 0.0101 | 0.364 | 11.384 | 13.272 | 7 | 97620757 | 97634029 | 1 | 1 |  |  |
| DMRT1 | IgH3.1482 | A | 0.00011 | 0.0101 | -0.324 | 655.5625 | 0.784 | 9 | 240219 | 241003 | 0 |  |  |  |
| PRB1 | IgH3.1837 | D | 0.00011 | 0.0101 | -0.369 | 924.3625 | 9.011 | 12 | 10478050 | 10487061 | 0 |  |  |  |
| WIT1 | IgH3.1748 | A | 0.00012 | 0.01038 | -0.253 | 492.487 | 1.106 | 11 | 31924094 | 31925200 | 0 |  |  |  |
| PRSS8 | IgH3.2225 | D | 0.00012 | 0.01038 | 0.183 | 255.886 | 2172.2 | 16 | 31882658 | 34054840 | 0 |  |  |  |
| BRP44 | CNVR396.1 | D | 0.00012 | 0.01052 | 0.438 | 674.778 | 2.432 | 1 | 166838889 | 166841321 | 0 |  |  |  |
| TMEM14C | CNVR2776.1 | D | 0.00012 | 0.01052 | 0.39 | 11.8245 | 4.064 | 6 | 10849095 | 10853159 | 0 |  |  |  |
| RCSD1 | CNVR396.1 | A | 0.00012 | 0.01065 | 0.467 | 933.57 | 2.432 | 1 | 166838889 | 166841321 | 0 |  |  |  |
| SNORD75 | Variation_31668 | A | 0.00012 | 0.01085 | -0.357 | 28.3515 | 1.097 | 1 | 172131569 | 172132666 | 0 |  |  |  |
| PSMB8 | Variation_32788 | D | 0.00013 | 0.01111 | -0.393 | 454.671 | 8.577 | 6 | 32467750 | 32476327 | 0 |  |  |  |
| H6PD | IgH3.8 | D | 0.00013 | 0.01118 | -0.477 | 979.8625 | 0.027 | 1 | 8255866 | 8255893 | 0 |  |  |  |
| ANO3 | CNVR5120.1 | D | 0.00013 | 0.01118 | 0.329 | 87.224 | 2.955 | 11 | 26564534 | 26567489 | 0 |  |  |  |
| JAM3 | IgH3.1818 | A | 0.00013 | 0.01131 | 0.254 | 162.8105 | 0.441 | 11 | 133322854 | 133323295 | 1 | 0 |  | YRI |
| CDSN | IgH3.978 | D | 0.00013 | 0.01131 | -0.405 | 115.5335 | 19.043 | 6 | 31318580 | 31337623 | 0 |  |  |  |
| C8orf33 | CNVR4125.1 | A | 0.00013 | 0.01137 | 0.331 | 596.379 | 6.121 | 8 | 145657105 | 145663226 | 0 |  |  |  |
| HDGF | IgH3.104 | A | 0.00013 | 0.01156 | -0.436 | 230.7925 | 22.842 | 1 | 155225558 | 155248400 | 1 | 1 |  |  |
| C2orf28 | IgH3.234 | D | 0.00013 | 0.01156 | -0.369 | 3.738 | 0.186 | 2 | 27294806 | 27294992 | 1 | 1 |  |  |
| AMBP | CNVR4450.1 | D | 0.00013 | 0.01156 | 0.318 | 868.2735 | 1.62 | 9 | 116740484 | 116742104 | 0 |  |  |  |
| CACNB2 | IgH3.1621 | A | 0.00014 | 0.01169 | -0.067 | 900.2755 | 0.438 | 10 | 19570647 | 19571085 | 0 |  |  |  |
| SAMM50 | IgH3.2598 | A | 0.00014 | 0.01169 | -0.304 | 263.912 | 11.135 | 22 | 42972648 | 42983783 | 0 |  |  |  |
| ELAC2 | IgH3.2290 | A | 0.00014 | 0.01187 | 0.496 | 524.357 | 0.03 | 17 | 13373252 | 13373282 | 0 |  |  |  |
| LEFTY1 | IgH3.179 | A | 0.00014 | 0.01194 | -0.483 | 402.0265 | 0.268 | 1 | 223740139 | 223740407 | 0 |  |  |  |
| KLHL22 | IgH3.2574 | D | 0.00014 | 0.01194 | -0.437 | 637.879 | 2.781 | 22 | 19792233 | 19795014 | 0 |  |  |  |
| PA2G4 | Variation_43857 | A | 0.00014 | 0.012 | -0.467 | 501.611 | 1.972 | 12 | 54288540 | 54290512 | 0 |  |  |  |
| B9D1 | CNVR7015.1 | D | 0.00014 | 0.012 | -0.372 | 738.0745 | 21.625 | 17 | 18469595 | 18491220 | 0 |  |  |  |
| GLRX2 | IgH3.147 | A | 0.00014 | 0.01206 | -0.441 | 139.0395 | 8.524 | 1 | 191480002 | 191488526 | 1 | 1 |  |  |
| NLRP11 | Variation_43991 | D | 0.00014 | 0.01206 | -0.343 | 542.2705 | 3.471 | 19 | 61558263 | 61561734 | 0 |  |  |  |
| KAT2A | IgH3.2315 | D | 0.00015 | 0.01236 | -0.347 | 805.88 | 3.93 | 17 | 38330626 | 38334556 | 0 |  |  |  |
| C12orf11 | CNVR5464.1 | A | 0.00015 | 0.01242 | 0.401 | 968.8685 | 6.064 | 12 | 26000113 | 26006177 | 0 |  |  |  |
| STAMBP | IgH3.274 | D | 0.00015 | 0.01248 | -0.51 | 49.679 | 11.268 | 2 | 73981869 | 73993137 | 0 |  |  |  |
| RIBC2 | IgH3.2599 | D | 0.00015 | 0.01248 | -0.366 | 683.689 | 1.1 | 22 | 43514458 | 43515558 | 0 |  |  |  |
| SUMO2 | Variation_49925 | D | 0.00015 | 0.01248 | -0.338 | 341.5095 | 14.121 | 17 | 70348607 | 70362728 | 0 |  |  |  |
| HNRNPA2B1 | IgH3.1147 | A | 0.00015 | 0.01254 | 0.38 | 676.013 | 0.03 | 7 | 25525511 | 25525541 | 1 |  |  |  |
| AMFR | IgH3.2228 | D | 0.00015 | 0.0126 | -0.447 | 643.8165 | 25.58 | 16 | 54353878 | 54379458 | 0 |  |  |  |
| ALDH16A1 | Variation_35661 | A | 0.00015 | 0.0126 | 0.462 | 16.29 | 12.864 | 19 | 54647342 | 54660206 | 0 |  |  |  |
| TM4SF18 | IgH3.516 | A | 0.00015 | 0.01272 | -0.174 | 813.8745 | 0.176 | 3 | 151341789 | 151341965 | 1 | 0 |  |  |
| GH2 | CNVR7165.1 | A | 0.00015 | 0.01272 | -0.204 | 902.739 | 97.754 | 17 | 60263746 | 60361500 | 0 |  |  |  |
| IL17F | CNVR2903.1 | A | 0.00016 | 0.01302 | -0.332 | 109.1565 | 11.23 | 6 | 52328121 | 52339351 | 0 |  |  |  |
| GADD45B | CNVR7483.1 | A | 0.00016 | 0.0132 | -0.512 | 134.8955 | 5.94 | 19 | 2296270 | 2302210 | 0 |  |  |  |
| ATP5C1 | IgH3.1609 | D | 0.00016 | 0.0132 | -0.451 | 981.016 | 14.09 | 10 | 6905962 | 6920052 | 0 |  |  |  |
| PCID2 | CNVR6061.3 | A | 0.00016 | 0.01326 | 0.363 | 664.97 | 31.233 | 13 | 113576064 | 113607297 | 0 |  |  |  |
| HDAC4 | CNVR1205.1 | D | 0.00016 | 0.01332 | -0.106 | 53.6445 | 3.265 | 2 | 239866467 | 239869732 | 0 |  |  |  |
| NAPEPLD | CNVR3517.2 | D | 0.00016 | 0.01338 | 0.239 | 809.4725 | 32.188 | 7 | 101758653 | 101790841 | 0 |  |  |  |
| RAD51C | Variation_32188 | D | 0.00017 | 0.0135 | 0.429 | 334.885 | 7.719 | 17 | 54465072 | 54472791 | 0 |  | JPT | |
| SLBP | CNVR1749.1 | A | 0.00017 | 0.01356 | 0.447 | 396.262 | 2.82 | 4 | 1279224 | 1282044 | 0 |  |  |  |
| MRPL40 | IgH3.2572 | D | 0.00017 | 0.01373 | 0.327 | 359.8545 | 0.306 | 22 | 18161823 | 18162129 | 0 |  |  |  |
| FAM173B | Variation_38586 | A | 0.00017 | 0.01385 | -0.388 | 888.9365 | 2.632 | 5 | 11181478 | 11184110 | 0 |  |  |  |
| SLCO1A2 | IgH3.1849 | D | 0.00017 | 0.01397 | -0.179 | 760.3005 | 0.21 | 12 | 22134625 | 22134835 | 0 |  |  |  |
| COX6A2 | IgH3.2225 | D | 0.00017 | 0.01397 | 0.196 | 550.3215 | 2172.2 | 16 | 31882658 | 34054840 | 0 |  |  |  |
| PRMT5 | IgH3.2031 | D | 0.00018 | 0.01414 | -0.455 | 938.735 | 0.305 | 14 | 21525454 | 21525759 | 0 |  |  |  |
| OR10W1 | Variation_38860 | A | 0.00018 | 0.01419 | -0.296 | 392.4055 | 4.436 | 11 | 57401386 | 57405822 | 0 |  |  |  |
| DNAJB6 | Variation_52170 | D | 0.00018 | 0.01419 | -0.242 | 56.0595 | 5.776 | 7 | 156809511 | 156815287 | 0 |  |  |  |
| C7orf31 | IgH3.1147 | A | 0.00018 | 0.01425 | -0.335 | 361.905 | 0.03 | 7 | 25525511 | 25525541 | 1 |  |  |  |
| C18orf19 | IgH3.2355 | A | 0.00018 | 0.01436 | -0.504 | 912.236 | 0.058 | 18 | 12772761 | 12772819 | 1 | 0 |  |  |
| DCLRE1B | IgH3.80 | D | 0.00018 | 0.01436 | -0.513 | 370.375 | 7.467 | 1 | 114627997 | 114635464 | 0 |  | CEU|CHB|JPT | |
| C3orf31 | IgH3.409 | D | 0.00018 | 0.01436 | 0.387 | 81.348 | 6.402 | 3 | 11919684 | 11926086 | 0 |  |  |  |
| LSM2 | IgH3.982 | A | 0.00019 | 0.01464 | 0.361 | 282.6075 | 26.427 | 6 | 32173758 | 32200185 | 0 |  |  |  |
| ETNK1 | CNVR5451.1 | A | 0.00019 | 0.01464 | 0.444 | 996.087 | 1.489 | 12 | 21706766 | 21708255 | 0 |  |  |  |
| C3orf31 | Variation_46278 | A | 0.00019 | 0.0147 | -0.457 | 160.7565 | 0.485 | 3 | 11674621 | 11675106 | 0 |  |  |  |
| OMA1 | IgH3.40 | A | 0.00019 | 0.0148 | -0.41 | 529.434 | 0.09 | 1 | 58222617 | 58222707 | 1 | 0 |  |  |
| GPAA1 | CNVR4125.1 | A | 0.00019 | 0.01513 | 0.397 | 442.7355 | 6.121 | 8 | 145657105 | 145663226 | 0 |  |  |  |
| TM7SF3 | Variation_49038 | A | 0.0002 | 0.01518 | -0.369 | 1022.041 | 6.695 | 12 | 26018497 | 26025192 | 0 |  |  |  |
| PPP1R3B | IgH3.1368 | A | 0.0002 | 0.01524 | 0.04 | 52.2945 | 6.135 | 8 | 9093765 | 9099900 | 0 |  |  |  |
| KBTBD7 | Variation_49211 | A | 0.0002 | 0.01534 | -0.556 | 990.6495 | 2.795 | 13 | 41657253 | 41660048 | 0 |  |  |  |
| S100A16 | IgH3.100 | D | 0.0002 | 0.01539 | -0.263 | 710.2695 | 0.247 | 1 | 151138918 | 151139165 | 0 |  |  |  |
| CCDC64 | IgH3.1927 | D | 0.0002 | 0.01539 | 0.251 | 493.8955 | 5.595 | 12 | 118473258 | 118478853 | 0 |  |  |  |
| MAST4 | Variation_51723 | A | 0.0002 | 0.0156 | -0.059 | 1101.536 | 12.985 | 5 | 65027976 | 65040961 | 0 |  |  |  |
| VARS | IgH3.982 | A | 0.00021 | 0.01571 | 0.383 | 298.0615 | 26.427 | 6 | 32173758 | 32200185 | 0 |  |  |  |
| TNFAIP3 | IgH3.1062 | D | 0.00021 | 0.01576 | 0.447 | 278.91 | 4.111 | 6 | 138519173 | 138523284 | 1 | 1 |  |  |
| SLC7A6OS | IgH3.2234 | A | 0.00021 | 0.01591 | 0.359 | 285.7005 | 2.356 | 16 | 67184073 | 67186429 | 1 | 0 |  |  |
| GTF2I | Variation_40989 | D | 0.00021 | 0.01601 | 0.301 | 78.108 | 0.241 | 7 | 73683474 | 73683715 | 0 |  |  |  |
| LMTK2 | IgH3.1204 | A | 0.00021 | 0.01606 | -0.367 | 98.352 | 0.044 | 7 | 97723879 | 97723923 | 1 | 1 |  |  |
| TMPRSS13 | IgH3.1801 | A | 0.00022 | 0.01641 | 0.323 | 205.694 | 0.748 | 11 | 117497015 | 117497763 | 1 | 1 |  |  |
| IDH1 | Variation_50385 | A | 0.00022 | 0.01641 | 0.081 | 394.153 | 1.038 | 2 | 208424990 | 208426028 | 0 |  |  |  |
| LCE3C | IgH3.99 | A | 0.00022 | 0.01646 | -0.533 | 249.966 | 7.037 | 1 | 151093458 | 151100495 | 0 |  |  |  |
| SYT14 | Variation_24051 | A | 0.00022 | 0.01646 | -0.395 | 70.5755 | 0.319 | 1 | 208361943 | 208362262 | 0 |  |  |  |
| TTRAP | IgH3.972 | A | 0.00022 | 0.01646 | 0.241 | 289.102 | 0.021 | 6 | 25055751 | 25055772 | 1 |  |  |  |
| EXOSC4 | CNVR4125.1 | A | 0.00022 | 0.01651 | 0.405 | 447.5205 | 6.121 | 8 | 145657105 | 145663226 | 0 |  |  |  |
| NUP133 | IgH3.181 | D | 0.00022 | 0.01661 | 0.462 | 590.261 | 0.203 | 1 | 228267551 | 228267754 | 1 | 0 |  |  |
| FMNL1 | IgH3.2319 | A | 0.00022 | 0.01661 | -0.495 | 83.271 | 1.166 | 17 | 40585082 | 40586248 | 0 |  |  |  |
| CDKN2D | CNVR7533.1 | A | 0.00022 | 0.01666 | -0.113 | 467.3285 | 2.239 | 19 | 10073175 | 10075414 | 0 |  |  |  |
| TMEM52 | CNVR23.3 | A | 0.00023 | 0.01671 | -0.312 | 692.9015 | 60.931 | 1 | 2563111 | 2624042 | 0 |  |  |  |
| DHRS11 | IgH3.2307 | A | 0.00023 | 0.0168 | 0.374 | 837.06 | 472.07 | 17 | 31425819 | 31897893 | 0 |  |  |  |
| BAT5 | IgH3.982 | A | 0.00023 | 0.01685 | 0.576 | 389.654 | 26.427 | 6 | 32173758 | 32200185 | 0 |  |  |  |
| UBAC2 | Variation_49283 | A | 0.00023 | 0.01685 | -0.564 | 858.779 | 8.913 | 13 | 97889635 | 97898548 | 0 |  |  |  |
| FAM155A | IgH3.2021 | D | 0.00023 | 0.01685 | -0.434 | 269.6165 | 0.944 | 13 | 106699026 | 106699970 | 0 |  |  |  |
| METTL3 | IgH3.2031 | D | 0.00023 | 0.01685 | -0.468 | 482.5925 | 0.305 | 14 | 21525454 | 21525759 | 0 |  |  |  |
| DRD1 | Variation_57050 | A | 0.00023 | 0.0169 | 0.133 | 921.282 | 1.089 | 5 | 173881287 | 173882376 | 0 |  |  |  |
| LYPD5 | Variation_55862 | A | 0.00023 | 0.01695 | -0.257 | 641.9055 | 6.337 | 19 | 49649357 | 49655694 | 0 |  |  |  |
| KCNJ2 | IgH3.2331 | A | 0.00023 | 0.017 | -0.29 | 288.6795 | 6.443 | 17 | 65397066 | 65403509 | 1 | 1 |  |  |
| FAM129B | CNVR4481.1 | D | 0.00023 | 0.01709 | -0.361 | 507.2355 | 2.942 | 9 | 129852970 | 129855912 | 0 |  |  |  |
| KIAA0182 | IgH3.2260 | A | 0.00023 | 0.01714 | -0.452 | 587.872 | 2.23 | 16 | 83648163 | 83650393 | 1 | 1 |  |  |
| MRPS25 | IgH3.411 | A | 0.00024 | 0.01728 | 0.543 | 415.529 | 0.234 | 3 | 15489067 | 15489301 | 1 | 1 |  |  |
| REXO1 | CNVR7483.1 | A | 0.00024 | 0.01733 | -0.465 | 510.452 | 5.94 | 19 | 2296270 | 2302210 | 0 |  |  |  |
| UFD1L | IgH3.2572 | D | 0.00024 | 0.01733 | 0.354 | 329.5695 | 0.306 | 22 | 18161823 | 18162129 | 0 |  |  |  |
| PSMD5 | IgH3.1574 | A | 0.00024 | 0.01742 | 0.249 | 582.1025 | 7.766 | 9 | 123217575 | 123225341 | 1 | 0 |  |  |
| CLCA1 | IgH3.55 | A | 0.00024 | 0.01742 | -0.157 | 211.7915 | 2.36 | 1 | 86935809 | 86938169 | 1 |  |  |  |
| DENND4B | Variation_43741 | A | 0.00024 | 0.01742 | -0.464 | 168.4535 | 3.779 | 1 | 152347532 | 152351311 | 0 |  |  |  |
| HCP5 | IgH3.982 | D | 0.00024 | 0.01751 | 0.368 | 620.2955 | 26.427 | 6 | 32173758 | 32200185 | 0 |  |  |  |
| SNORD36A | IgH3.1584 | D | 0.00024 | 0.01751 | 0.286 | 439.949 | 0.202 | 9 | 135647217 | 135647419 | 0 |  |  |  |
| MTG1 | IgH3.1712 | A | 0.00024 | 0.01756 | 0.274 | 99.203 | 224.97 | 10 | 135084170 | 135309142 | 0 |  |  |  |
| TARBP1 | IgH3.185 | A | 0.00024 | 0.0176 | 0.334 | 1020.62 | 11.358 | 1 | 233663875 | 233675233 | 0 |  |  |  |
| PINX1 | IgH3.1371 | A | 0.00025 | 0.01765 | -0.422 | 217.729 | 0.106 | 8 | 10479825 | 10479931 | 1 | 0 |  |  |
| EIF3K | Variation_30904 | A | 0.00025 | 0.01774 | -0.419 | 156.9665 | 4.603 | 19 | 43969766 | 43974369 | 0 |  |  |  |
| CYP2U1 | IgH3.707 | A | 0.00025 | 0.01779 | -0.445 | 145.97 | 0.041 | 4 | 109229104 | 109229145 | 1 |  |  |  |
| CACNG3 | IgH3.2220 | D | 0.00025 | 0.01783 | 0.279 | 805.8805 | 4.497 | 16 | 23424175 | 23428672 | 1 | 0 |  |  |
| MLX | IgH3.2315 | D | 0.00025 | 0.01792 | -0.366 | 352.986 | 3.93 | 17 | 38330626 | 38334556 | 0 |  |  |  |
| CRLS1 | IgH3.2490 | A | 0.00025 | 0.01792 | -0.518 | 383.2115 | 43.896 | 20 | 6356877 | 6400773 | 0 |  |  |  |
| CNOT6L | IgH3.683 | D | 0.00026 | 0.01826 | -0.182 | 577.991 | 0.046 | 4 | 79484580 | 79484626 | 0 |  |  |  |
| C1orf161 | IgH3.83 | A | 0.00026 | 0.01831 | 0.304 | 328.534 | 0 | 1 | 116796175 | 116796175 | 1 |  |  |  |
| VANGL1 | IgH3.82 | A | 0.00026 | 0.01835 | -0.46 | 830.6625 | 0.205 | 1 | 115183672 | 115183877 | 0 |  |  |  |
| HSPB11 | CNVR185.1 | D | 0.00026 | 0.0184 | 0.412 | 12.671 | 0.713 | 1 | 54184876 | 54185589 | 0 |  |  |  |
| SGK269 | IgH3.2161 | A | 0.00026 | 0.0185 | 0.014 | 195.079 | 1.541 | 15 | 75081648 | 75083189 | 0 |  |  |  |
| C15orf33 | CNVR6384.1 | D | 0.00027 | 0.01864 | -0.387 | 223.626 | 3.095 | 15 | 47332068 | 47335163 | 0 |  |  |  |
| SNX5 | IgH3.2501 | D | 0.00027 | 0.01878 | 0.364 | 329.5205 | 4.94 | 20 | 18215689 | 18220629 | 0 |  |  |  |
| IKZF2 | IgH3.372 | A | 0.00027 | 0.01892 | -0.162 | 569.554 | 0.34 | 2 | 213078595 | 213078935 | 0 |  |  |  |
| COL28A1 | Variation_57667 | D | 0.00027 | 0.01892 | -0.349 | 745.3305 | 0.852 | 7 | 6708472 | 6709324 | 0 |  |  |  |
| FZD8 | IgH3.1638 | A | 0.00027 | 0.01896 | -0.33 | 290.9995 | 0.227 | 10 | 36259888 | 36260115 | 0 |  |  |  |
| SYNGR3 | CNVR6589.2 | D | 0.00028 | 0.0191 | 0.306 | 807.9085 | 1.32 | 16 | 1174874 | 1176194 | 0 |  | CEU | |
| ANKK1 | CNVR5313.1 | D | 0.00028 | 0.01919 | -0.25 | 841.2895 | 0.925 | 11 | 111929209 | 111930134 | 0 |  |  |  |
| AARSD1 | IgH3.2315 | A | 0.00028 | 0.01924 | -0.333 | 42.147 | 3.93 | 17 | 38330626 | 38334556 | 0 |  |  |  |
| TPM4 | Variation_50089 | D | 0.00028 | 0.01947 | 0.247 | 157.56 | 22.753 | 19 | 16230410 | 16253163 | 0 |  |  |  |
| KRT15 | Variation_49857 | A | 0.00029 | 0.01951 | 0.188 | 790.935 | 4.067 | 17 | 36137258 | 36141325 | 0 |  |  |  |
| FKBPL | IgH3.982 | A | 0.00029 | 0.0196 | 0.423 | 44.7085 | 26.427 | 6 | 32173758 | 32200185 | 0 |  |  |  |
| YARS2 | IgH3.1861 | A | 0.00029 | 0.01969 | 0.453 | 575.146 | 0.202 | 12 | 32220397 | 32220599 | 0 |  |  |  |
| RWDD2B | IgH3.2554 | D | 0.00029 | 0.01973 | 0.509 | 150.3125 | 10.713 | 21 | 29161798 | 29172511 | 0 |  |  |  |
| PSMA2 | IgH3.1156 | A | 0.00029 | 0.01982 | -0.439 | 825.5825 | 0.609 | 7 | 42105380 | 42105989 | 1 | 0 |  |  |
| C18orf22 | IgH3.2413 | A | 0.00029 | 0.01982 | 0.459 | 67.5465 | 13.722 | 18 | 75977894 | 75991616 | 1 | 1 |  |  |
| EMILIN2 | IgH3.2347 | A | 0.00029 | 0.01991 | 0.402 | 373.7145 | 0.374 | 18 | 3244460 | 3244834 | 1 | 0 |  |  |
| DLGAP2 | IgH3.1284 | A | 0.00029 | 0.01991 | 0.368 | 972.426 | 19.48 | 8 | 577826 | 597306 | 0 |  |  |  |
| OR13C8 | Variation_52659 | A | 0.00029 | 0.01991 | -0.112 | 40.9385 | 2.02 | 9 | 106331822 | 106333842 | 0 |  |  |  |
| TP53I3 | Variation_50455 | A | 0.0003 | 0.01995 | 0.431 | 847.8585 | 4.295 | 2 | 25007490 | 25011785 | 0 |  |  |  |
| KIF27 | Variation_31520 | D | 0.0003 | 0.01995 | 0.276 | 84.9285 | 122.64 | 9 | 85660189 | 85782828 | 0 |  |  |  |
| MTMR11 | Variation_48133 | A | 0.0003 | 0.02 | -0.475 | 763.419 | 3.732 | 1 | 148936576 | 148940308 | 0 |  |  |  |
| SLC37A1 | CNVR8020.1 | A | 0.0003 | 0.02004 | -0.449 | 676.691 | 1.075 | 21 | 42157561 | 42158636 | 0 |  |  |  |
| ITIH2 | Variation_48628 | D | 0.0003 | 0.02008 | -0.283 | 451.4775 | 0.163 | 10 | 7356969 | 7357132 | 0 |  |  |  |
| OR7D2 | IgH3.2427 | D | 0.0003 | 0.02021 | -0.284 | 24.9455 | 3.449 | 19 | 9135660 | 9139109 | 0 |  |  |  |
| DAB1 | IgH3.40 | A | 0.0003 | 0.02034 | -0.422 | 360.0895 | 0.09 | 1 | 58222617 | 58222707 | 1 | 0 |  |  |
| SNORD108 | IgH3.2113 | D | 0.0003 | 0.02034 | 0.285 | 1110.708 | 438.32 | 15 | 21891651 | 22329972 | 0 |  |  |  |
| PI4KAP2 | IgH3.2573 | A | 0.0003 | 0.02034 | -0.341 | 896.841 | 0.41 | 22 | 19282897 | 19283307 | 0 |  |  |  |
| HSPA8 | IgH3.1803 | D | 0.00031 | 0.02039 | 0.45 | 910.824 | 0.203 | 11 | 121525009 | 121525212 | 1 | 1 |  |  |
| RGS1 | IgH3.145 | A | 0.00031 | 0.02071 | 0.057 | 608.0745 | 0.836 | 1 | 191422123 | 191422959 | 1 | 1 |  |  |
| SIGLEC10 | IgH3.2468 | D | 0.00032 | 0.02099 | -0.389 | 202.8005 | 23.669 | 19 | 56823613 | 56847282 | 0 |  |  |  |
| SIGLEC7 | Variation_50197 | A | 0.00032 | 0.02104 | -0.214 | 322.9415 | 5.407 | 19 | 56022744 | 56028151 | 0 |  |  |  |
| SCARNA21 | IgH3.2282 | D | 0.00032 | 0.02122 | -0.452 | 437.933 | 0.186 | 17 | 8188260 | 8188446 | 0 |  |  |  |
| TRAF3IP3 | IgH3.167 | A | 0.00032 | 0.02127 | 0.455 | 779.601 | 1.318 | 1 | 208789477 | 208790795 | 0 |  |  |  |
| C16orf61 | IgH3.2252 | D | 0.00033 | 0.02131 | 0.458 | 2.932 | 2.98 | 16 | 79581160 | 79584140 | 0 |  |  |  |
| NEUROD1 | IgH3.347 | A | 0.00033 | 0.0214 | 0.191 | 726.0975 | 0.323 | 2 | 181525596 | 181525919 | 1 | 0 |  |  |
| CHCHD8 | Variation_48880 | A | 0.00033 | 0.0214 | -0.388 | 93.263 | 10.46 | 11 | 73361942 | 73372402 | 0 |  |  |  |
| ZNF718 | CNVR1731.1 | D | 0.00033 | 0.02145 | 0.334 | 445.15 | 7.17 | 4 | 543618 | 550788 | 0 |  |  |  |
| S100A11 | IgH3.99 | D | 0.00033 | 0.02167 | -0.169 | 816.0695 | 7.037 | 1 | 151093458 | 151100495 | 0 |  |  |  |
| PRSS36 | IgH3.2225 | D | 0.00033 | 0.02167 | 0.163 | 266.765 | 2172.2 | 16 | 31882658 | 34054840 | 0 |  |  |  |
| FAM122A | IgH3.1536 | D | 0.00034 | 0.02189 | -0.415 | 120.1695 | 0.253 | 9 | 70706902 | 70707155 | 0 |  |  |  |
| ETFB | Variation_50198 | D | 0.00034 | 0.02189 | 0.272 | 106.9585 | 7.889 | 19 | 56442050 | 56449939 | 0 |  |  |  |
| VPS29 | CNVR5667.1 | A | 0.00034 | 0.02198 | 0.547 | 798.594 | 2.945 | 12 | 108621884 | 108624829 | 0 |  |  |  |
| AKAP8 | IgH3.2439 | D | 0.00034 | 0.02198 | -0.362 | 167.423 | 0.125 | 19 | 15171108 | 15171233 | 0 |  |  |  |
| STT3A | IgH3.1807 | D | 0.00034 | 0.02198 | 0.331 | 255.237 | 4.248 | 11 | 125239580 | 125243828 | 0 |  |  |  |
| RBL2 | IgH3.2227 | D | 0.00034 | 0.02198 | 0.231 | 908.1225 | 9.703 | 16 | 52967430 | 52977133 | 0 |  |  |  |
| C2orf16 | IgH3.234 | D | 0.00034 | 0.02216 | -0.392 | 361.2795 | 0.186 | 2 | 27294806 | 27294992 | 1 | 1 |  |  |
| C7orf30 | IgH3.1145 | A | 0.00034 | 0.02216 | 0.412 | 966.4285 | 0.914 | 7 | 24277470 | 24278384 | 0 |  |  |  |
| TUBG1 | IgH3.2315 | D | 0.00035 | 0.02233 | -0.33 | 310.8295 | 3.93 | 17 | 38330626 | 38334556 | 0 |  |  |  |
| DLX6 | IgH3.1201 | A | 0.00035 | 0.02237 | -0.074 | 779.773 | 0.003 | 7 | 97255531 | 97255534 | 0 |  |  |  |
| TMEM132B | CNVR5716.1 | D | 0.00035 | 0.02237 | -0.332 | 597.9355 | 4.771 | 12 | 123947778 | 123952549 | 0 |  |  |  |
| CCDC77 | IgH3.1826 | D | 0.00035 | 0.0225 | 0.404 | 397.725 | 4.017 | 12 | 795155 | 799172 | 0 |  |  |  |
| ADAM7 | IgH3.1399 | A | 0.00035 | 0.02259 | -0.423 | 629.494 | 19.486 | 8 | 25027546 | 25047032 | 0 |  |  |  |
| GBP3 | IgH3.56 | D | 0.00036 | 0.02263 | 0.45 | 4.91 | 1.766 | 1 | 89249015 | 89250781 | 0 |  | CEU|CHB|JPT | CEU|JPT |
| SLC35A3 | CNVR256.1 | A | 0.00036 | 0.02292 | -0.363 | 610.152 | 8.809 | 1 | 99629113 | 99637922 | 0 |  |  |  |
| OR13C3 | Variation_52659 | A | 0.00036 | 0.02297 | -0.173 | 7.581 | 2.02 | 9 | 106331822 | 106333842 | 0 |  |  |  |
| SLMO2 | IgH3.2518 | A | 0.00037 | 0.02325 | -0.501 | 844.3475 | 0.441 | 20 | 56202318 | 56202759 | 1 | 0 |  |  |
| LASS2 | Variation_33510 | D | 0.00037 | 0.0233 | -0.35 | 946.6835 | 0.139 | 1 | 148262554 | 148262693 | 0 |  |  |  |
| EPB42 | CNVR6363.1 | A | 0.00037 | 0.02349 | -0.313 | 781.2465 | 2.287 | 15 | 40508563 | 40510850 | 0 |  |  |  |
| SNX5 | Variation_56238 | A | 0.00038 | 0.02372 | 0.424 | 832.936 | 0.863 | 20 | 17051194 | 17052057 | 0 |  |  |  |
| EFTUD2 | IgH3.2319 | D | 0.00038 | 0.02395 | 0.377 | 276.6495 | 1.166 | 17 | 40585082 | 40586248 | 0 |  |  |  |
| KIAA0415 | CNVR3259.1 | D | 0.00039 | 0.02405 | 0.231 | 718.093 | 0.65 | 7 | 4072089 | 4072739 | 0 |  | YRI | |
| RASGRP4 | CNVR7633.1 | D | 0.00039 | 0.02405 | -0.465 | 568.672 | 3.806 | 19 | 43033392 | 43037198 | 0 |  |  |  |
| GGH | IgH3.1423 | D | 0.00039 | 0.02409 | -0.346 | 612.4825 | 0.693 | 8 | 63490044 | 63490737 | 1 | 0 |  |  |
| EXOSC2 | Variation_57977 | D | 0.00039 | 0.02409 | -0.303 | 187.7955 | 9.416 | 9 | 132381538 | 132390954 | 0 |  |  |  |
| NOXA1 | CNVR4553.1 | D | 0.0004 | 0.02469 | 0.32 | 573.335 | 171.12 | 9 | 140102069 | 140273191 | 0 |  |  |  |
| TNFAIP3 | Variation_57221 | D | 0.0004 | 0.02491 | 0.424 | 883.544 | 0.951 | 6 | 137355139 | 137356090 | 0 |  |  |  |
| C16orf38 | IgH3.2191 | A | 0.00041 | 0.02496 | -0.355 | 532.0085 | 0.306 | 16 | 945349 | 945655 | 0 |  |  |  |
| HCG22 | IgH3.978 | A | 0.00041 | 0.02518 | -0.415 | 176.2615 | 19.043 | 6 | 31318580 | 31337623 | 0 |  |  |  |
| PAR1 | IgH3.2113 | D | 0.00041 | 0.02523 | 0.424 | 1260.597 | 438.32 | 15 | 21891651 | 22329972 | 0 |  |  |  |
| TMEM208 | CNVR6757.1 | D | 0.00041 | 0.02532 | -0.279 | 301.763 | 2.543 | 16 | 66122634 | 66125177 | 0 |  |  |  |
| PPP4R1L | IgH3.2518 | A | 0.00042 | 0.02549 | 0.395 | 77.472 | 0.441 | 20 | 56202318 | 56202759 | 1 | 0 |  |  |
| C7orf31 | IgH3.1145 | A | 0.00043 | 0.02593 | -0.487 | 886.578 | 0.914 | 7 | 24277470 | 24278384 | 0 |  |  |  |
| C15orf57 | IgH3.2130 | D | 0.00043 | 0.02593 | 0.273 | 1.6535 | 0.13 | 15 | 38636978 | 38637108 | 0 |  |  |  |
| RYBP | IgH3.452 | D | 0.00044 | 0.02628 | -0.435 | 111.1825 | 2.569 | 3 | 72432553 | 72435122 | 1 | 1 |  |  |
| PSG5 | IgH3.2460 | A | 0.00044 | 0.02628 | -0.409 | 985.6295 | 0.683 | 19 | 47387843 | 47388526 | 1 | 1 |  |  |
| C12orf66 | Variation_43862 | D | 0.00044 | 0.02628 | 0.371 | 415.6785 | 1.053 | 12 | 63303719 | 63304772 | 0 |  |  |  |
| WTIP | IgH3.2457 | A | 0.00044 | 0.02632 | -0.246 | 857.9445 | 17.565 | 19 | 40541049 | 40558614 | 0 |  |  |  |
| RBM18 | IgH3.1574 | A | 0.00044 | 0.02649 | 0.281 | 840.617 | 7.766 | 9 | 123217575 | 123225341 | 1 | 0 |  |  |
| SLITRK6 | Variation_45209 | D | 0.00044 | 0.02662 | -0.439 | 433.9705 | 0.933 | 13 | 84834699 | 84835632 | 0 |  |  |  |
| ATP5C1 | Variation_48636 | A | 0.00044 | 0.02667 | 0.357 | 242.7355 | 7.015 | 10 | 8126176 | 8133191 | 0 |  |  |  |
| FLI1 | IgH3.1812 | A | 0.00045 | 0.02696 | 0.434 | 281.054 | 0.305 | 11 | 127847083 | 127847388 | 1 | 0 |  |  |
| CCT8 | IgH3.2554 | D | 0.00045 | 0.02696 | 0.302 | 202.758 | 10.713 | 21 | 29161798 | 29172511 | 0 |  |  |  |
| MMP14 | CNVR6087.1 | D | 0.00046 | 0.02713 | -0.103 | 626.853 | 0.459 | 14 | 23008220 | 23008679 | 0 |  |  |  |
| OR10Q1 | Variation_38860 | A | 0.00046 | 0.02726 | -0.303 | 353.2795 | 4.436 | 11 | 57401386 | 57405822 | 0 |  |  |  |
| C2orf83 | CNVR1159.1 | D | 0.00046 | 0.0273 | -0.264 | 961.6175 | 2.708 | 2 | 229157636 | 229160344 | 0 |  |  |  |
| PEX6 | IgH3.990 | A | 0.00046 | 0.02751 | 0.43 | 99.245 | 0.357 | 6 | 43146697 | 43147054 | 1 | 0 | CEU|CHB|JPT | |
| ANAPC7 | CNVR5667.1 | A | 0.00047 | 0.02755 | 0.485 | 690.091 | 2.945 | 12 | 108621884 | 108624829 | 0 |  |  |  |
| CDK4 | IgH3.1884 | D | 0.00047 | 0.02755 | 0.355 | 924.151 | 2.494 | 12 | 57355748 | 57358242 | 0 |  |  |  |
| FLJ35220 | Variation_30854 | D | 0.00047 | 0.02759 | 0.364 | 311.0005 | 3.769 | 17 | 75702047 | 75705816 | 0 |  |  |  |
| BDH1 | CNVR1724.1 | D | 0.00047 | 0.02763 | 0.357 | 224.57 | 2.869 | 3 | 198970157 | 198973026 | 0 |  |  |  |
| THYN1 | IgH3.1818 | A | 0.00047 | 0.02772 | 0.368 | 303.2925 | 0.441 | 11 | 133322854 | 133323295 | 1 | 0 |  |  |
| LACRT | Variation_43857 | A | 0.00047 | 0.02772 | -0.4 | 974.6445 | 1.972 | 12 | 54288540 | 54290512 | 0 |  |  |  |
| CSNK1E | IgH3.2594 | A | 0.00047 | 0.02772 | -0.459 | 627.2385 | 45.468 | 22 | 37679973 | 37725441 | 0 |  |  |  |
| C4BPA | CNVR480.1 | A | 0.00048 | 0.02796 | -0.22 | 917.75 | 5.315 | 1 | 204449492 | 204454807 | 0 |  |  |  |
| SPCS3 | CNVR2178.1 | D | 0.00048 | 0.02796 | -0.319 | 198.311 | 1.281 | 4 | 177286565 | 177287846 | 0 |  |  |  |
| MYH2 | IgH3.2283 | D | 0.00048 | 0.028 | -0.448 | 568.276 | 0 | 17 | 9811151 | 9811151 | 0 |  |  |  |
| HUS1B | IgH3.957 | A | 0.00048 | 0.02804 | -0.184 | 532.0465 | 186.26 | 6 | 162536 | 348799 | 0 |  |  |  |
| PARD6A | IgH3.2234 | D | 0.00048 | 0.02809 | 0.421 | 929.6285 | 2.356 | 16 | 67184073 | 67186429 | 1 | 0 |  |  |
| TARBP1 | CNVR543.1 | D | 0.00048 | 0.02817 | -0.323 | 805.0785 | 0.78 | 1 | 231832888 | 231833668 | 0 |  |  |  |
| C2orf44 | Variation_50455 | A | 0.00049 | 0.02873 | 0.478 | 890.588 | 4.295 | 2 | 25007490 | 25011785 | 0 |  |  |  |
| C1orf123 | CNVR185.1 | D | 0.0005 | 0.02877 | 0.372 | 728.9015 | 0.713 | 1 | 54184876 | 54185589 | 0 |  |  |  |
| PRUNE | Variation_33510 | D | 0.0005 | 0.02877 | -0.251 | 998.72 | 0.139 | 1 | 148262554 | 148262693 | 0 |  |  |  |
| C5orf49 | CNVR2336.1 | A | 0.0005 | 0.02877 | -0.309 | 73.9585 | 3.447 | 5 | 7970069 | 7973516 | 0 |  |  |  |
| RBM16 | Variation_51828 | A | 0.0005 | 0.02905 | 0.467 | 557.704 | 23.545 | 6 | 155716021 | 155739566 | 0 |  |  |  |
| SPIRE1 | IgH3.2353 | A | 0.00051 | 0.02917 | -0.144 | 686.097 | 0.737 | 18 | 11855892 | 11856629 | 0 |  |  |  |
| ERLIN2 | IgH3.1406 | D | 0.00051 | 0.02921 | -0.366 | 295.6155 | 0.29 | 8 | 37428395 | 37428685 | 1 | 1 |  |  |
| HSBP1 | IgH3.2255 | D | 0.00051 | 0.02921 | -0.43 | 959.04 | 0.72 | 16 | 81442914 | 81443634 | 1 | 0 |  |  |
| PLOD3 | IgH3.1207 | A | 0.00051 | 0.02921 | -0.434 | 204.4775 | 0.217 | 7 | 100846440 | 100846657 | 0 |  |  |  |
| TRMT61B | IgH3.236 | D | 0.00051 | 0.02937 | -0.306 | 882.9745 | 3.335 | 2 | 28055129 | 28058464 | 0 |  |  |  |
| USP18 | IgH3.2570 | A | 0.00052 | 0.02956 | -0.27 | 42.187 | 288.31 | 22 | 17128428 | 17416739 | 0 |  |  |  |
| DOT1L | CNVR7483.1 | A | 0.00052 | 0.02964 | -0.415 | 143.938 | 5.94 | 19 | 2296270 | 2302210 | 0 |  |  |  |
| YIF1B | Variation_30904 | A | 0.00052 | 0.02968 | -0.397 | 475.3025 | 4.603 | 19 | 43969766 | 43974369 | 0 |  |  |  |
| SLC2A9 | IgH3.584 | A | 0.00052 | 0.02972 | -0.265 | 563.0215 | 0.044 | 4 | 10097622 | 10097666 | 0 |  |  |  |
| POLR1C | IgH3.990 | A | 0.00052 | 0.02983 | 0.205 | 448.4775 | 0.357 | 6 | 43146697 | 43147054 | 1 | 0 |  | JPT |
| C1orf94 | CNVR139.1 | D | 0.00053 | 0.02995 | 0.297 | 517.5505 | 2.044 | 1 | 34954800 | 34956844 | 0 |  |  |  |
| TAS2R3 | IgH3.1245 | A | 0.00053 | 0.02995 | -0.469 | 792.838 | 206.43 | 7 | 142006970 | 142213403 | 0 |  |  |  |
| RCSD1 | IgH3.114 | A | 0.00053 | 0.03006 | -0.456 | 1016.635 | 0.1 | 1 | 164887518 | 164887618 | 0 |  |  |  |
| TBCE | IgH3.185 | A | 0.00053 | 0.03018 | 0.327 | 20.0695 | 11.358 | 1 | 233663875 | 233675233 | 0 |  |  |  |
| ALG1 | Variation_42108 | A | 0.00053 | 0.03018 | 0.175 | 643.495 | 0.445 | 16 | 5713312 | 5713757 | 0 |  |  |  |
| MRPL24 | IgH3.104 | D | 0.00055 | 0.03074 | -0.454 | 238.505 | 22.842 | 1 | 155225558 | 155248400 | 1 | 1 |  |  |
| ZBTB38 | Variation_50947 | D | 0.00055 | 0.03078 | -0.451 | 296.0195 | 1.565 | 3 | 142885335 | 142886900 | 0 |  |  |  |
| HLA-DQB2 | IgH3.985 | A | 0.00055 | 0.03078 | -0.38 | 395.427 | 194.94 | 6 | 32537621 | 32732557 | 0 |  |  |  |
| POMT2 | IgH3.2077 | A | 0.00055 | 0.03089 | -0.405 | 186.1955 | 0.2 | 14 | 76647920 | 76648120 | 0 |  |  |  |
| PSORS1C1 | IgH3.979 | A | 0.00056 | 0.03136 | -0.331 | 166.322 | 22.957 | 6 | 31381009 | 31403966 | 0 |  |  |  |
| SLC6A17 | IgH3.77 | A | 0.00056 | 0.0314 | -0.218 | 639.9385 | 22.913 | 1 | 111171895 | 111194808 | 0 |  |  |  |
| GAMT | CNVR7483.1 | A | 0.00057 | 0.03167 | 0.212 | 942.9805 | 5.94 | 19 | 2296270 | 2302210 | 0 |  |  |  |
| OR4A47 | Variation_23169 | D | 0.00057 | 0.03179 | 0.227 | 147.8 | 8.566 | 11 | 48323868 | 48332434 | 0 |  |  |  |
| OR5P2 | IgH3.1725 | D | 0.00057 | 0.03179 | -0.522 | 19.248 | 28.543 | 11 | 7769604 | 7798147 | 0 |  |  |  |
| MMP2 | IgH3.2228 | A | 0.00057 | 0.03179 | -0.144 | 255.5575 | 25.58 | 16 | 54353878 | 54379458 | 0 |  |  |  |
| SLC36A2 | CNVR2648.1 | A | 0.00058 | 0.03202 | -0.335 | 77.518 | 2.88 | 5 | 150769996 | 150772876 | 0 |  |  |  |
| DUOXA2 | CNVR6378.1 | A | 0.00058 | 0.03202 | -0.314 | 826.5945 | 0.556 | 15 | 44022575 | 44023131 | 0 |  |  |  |
| LDHB | CNVR5451.1 | D | 0.00058 | 0.03217 | 0.336 | 15.229 | 1.489 | 12 | 21706766 | 21708255 | 0 |  |  |  |
| S100A11 | IgH3.95 | A | 0.00059 | 0.03228 | 0.341 | 692.1385 | 4.257 | 1 | 149583860 | 149588117 | 0 |  |  |  |
| MGC13005 | IgH3.301 | D | 0.00059 | 0.0324 | -0.371 | 4.5225 | 0.002 | 2 | 114078872 | 114078874 | 0 |  |  |  |
| FUZ | IgH3.2467 | A | 0.00059 | 0.03243 | 0.368 | 420.6705 | 1.968 | 19 | 55426812 | 55428780 | 0 |  |  |  |
| NDUFC1 | IgH3.750 | D | 0.00059 | 0.03251 | 0.245 | 215.6005 | 0.01 | 4 | 140217878 | 140217888 | 1 | 1 |  |  |
| AKR7A2 | CNVR104.1 | A | 0.00059 | 0.03259 | -0.298 | 979.3625 | 1.436 | 1 | 20487218 | 20488654 | 0 |  |  |  |
| ZNF236 | IgH3.2407 | D | 0.0006 | 0.0327 | 0.43 | 331.8625 | 0.092 | 18 | 72406570 | 72406662 | 1 | 1 |  |  |
| RAX | IgH3.2381 | A | 0.0006 | 0.033 | -0.213 | 204.34 | 0.663 | 18 | 55293097 | 55293760 | 1 | 0 |  |  |
| RARS | IgH3.942 | A | 0.00061 | 0.03325 | -0.373 | 234.531 | 0.655 | 5 | 168097322 | 168097977 | 1 | 0 |  |  |
| BACE1 | Variation_48706 | D | 0.00061 | 0.03325 | 0.274 | 787.9935 | 1.481 | 11 | 117465637 | 117467118 | 0 |  |  |  |
| ZNF252 | CNVR4130.1 | D | 0.00061 | 0.03329 | 0.382 | 389.6675 | 3.214 | 8 | 145796373 | 145799587 | 0 |  |  |  |
| LCN10 | CNVR4543.1 | A | 0.00061 | 0.03336 | -0.287 | 637.521 | 0.981 | 9 | 139392847 | 139393828 | 0 |  |  |  |
| GMNN | IgH3.972 | A | 0.00062 | 0.0334 | 0.185 | 167.041 | 0.021 | 6 | 25055751 | 25055772 | 1 |  |  |  |
| AKAP8L | IgH3.2436 | A | 0.00062 | 0.03344 | -0.364 | 535.333 | 2.181 | 19 | 14837103 | 14839284 | 1 | 1 |  |  |
| FGF12 | CNVR1678.1 | A | 0.00062 | 0.03347 | -0.279 | 103.6195 | 1.068 | 3 | 193372869 | 193373937 | 0 |  |  |  |
| AFMID | IgH3.2335 | A | 0.00062 | 0.03355 | 0.099 | 90.1495 | 5.375 | 17 | 73792951 | 73798326 | 0 |  |  |  |
| C7orf34 | IgH3.1249 | A | 0.00062 | 0.03358 | -0.295 | 993.35 | 0.531 | 7 | 143341017 | 143341548 | 0 |  |  |  |
| NEGR1 | IgH3.49 | D | 0.00063 | 0.03387 | 0.237 | 400.1565 | 75.924 | 1 | 72519157 | 72595081 | 0 |  |  |  |
| CCT2 | IgH3.1892 | A | 0.00063 | 0.03398 | 0.267 | 797.3105 | 0.067 | 12 | 69070893 | 69070960 | 1 | 0 |  |  |
| TC2N | IgH3.2095 | A | 0.00063 | 0.03398 | 0.341 | 76.766 | 0.527 | 14 | 91421340 | 91421867 | 1 | 1 |  |  |
| DPPA5 | IgH3.1015 | A | 0.00063 | 0.03398 | 0.121 | 642.6705 | 7.312 | 6 | 74766417 | 74773729 | 0 |  |  |  |
| IGF2R | Variation_36473 | D | 0.00063 | 0.03402 | 0.267 | 736.725 | 13.251 | 6 | 161122197 | 161135448 | 0 |  |  |  |
| C6orf221 | Variation_33814 | D | 0.00064 | 0.03428 | -0.257 | 956.908 | 0.769 | 6 | 75087162 | 75087931 | 0 |  |  |  |
| LSM7 | CNVR7483.1 | A | 0.00065 | 0.03461 | 0.283 | 17.2335 | 5.94 | 19 | 2296270 | 2302210 | 0 |  |  |  |
| SIPA1L3 | Variation_30904 | D | 0.00065 | 0.03464 | 0.357 | 727.187 | 4.603 | 19 | 43969766 | 43974369 | 0 |  |  |  |
| HMMR | IgH3.939 | A | 0.00067 | 0.0353 | 0.413 | 566.5185 | 9.614 | 5 | 163407136 | 163416750 | 1 | 0 |  |  |
| PIH1D1 | IgH3.2467 | A | 0.00067 | 0.0354 | 0.419 | 781.684 | 1.968 | 19 | 55426812 | 55428780 | 0 |  |  |  |
| HLX | IgH3.175 | A | 0.00068 | 0.03565 | 0.17 | 460.254 | 0.156 | 1 | 219582526 | 219582682 | 1 | 0 |  |  |
| UCK2 | IgH3.114 | A | 0.00069 | 0.03604 | -0.485 | 783.73 | 0.1 | 1 | 164887518 | 164887618 | 0 |  |  |  |
| CDH7 | IgH3.2394 | A | 0.00069 | 0.03608 | -0.331 | 280.0695 | 8.455 | 18 | 61918108 | 61926563 | 0 |  |  |  |
| UACA | IgH3.2155 | A | 0.00069 | 0.03611 | 0.043 | 642.094 | 0.644 | 15 | 69430841 | 69431485 | 1 | 1 |  |  |
| CBFA2T3 | CNVR6874.2 | A | 0.00069 | 0.03611 | -0.452 | 68.744 | 9.905 | 16 | 87593531 | 87603436 | 0 |  |  |  |
| PGM2 | IgH3.622 | A | 0.00069 | 0.03618 | -0.42 | 413.635 | 0.264 | 4 | 37936582 | 37936846 | 1 | 0 |  |  |
| C12orf36 | IgH3.1842 | A | 0.0007 | 0.03636 | -0.225 | 993.957 | 6.661 | 12 | 12427474 | 12434135 | 0 |  |  |  |
| FETUB | IgH3.552 | A | 0.0007 | 0.0366 | 0.295 | 112.732 | 2.061 | 3 | 187735465 | 187737526 | 1 | 1 |  |  |
| DLC1 | IgH3.1373 | A | 0.0007 | 0.0366 | -0.391 | 1589.385 | 642.01 | 8 | 11932642 | 12574653 | 0 |  |  |  |
| KIAA0556 | CNVR6680.1 | D | 0.00071 | 0.03667 | -0.46 | 601.597 | 0.625 | 16 | 26982796 | 26983421 | 0 |  |  |  |
| ZNF613 | CNVR7705.2 | A | 0.00071 | 0.03695 | 0.341 | 422.11 | 1.465 | 19 | 57554521 | 57555986 | 0 |  |  |  |
| LARP1 | CNVR2657.1 | A | 0.00072 | 0.03698 | -0.173 | 246.115 | 4.828 | 5 | 154373534 | 154378362 | 0 |  |  |  |
| OSR1 | IgH3.231 | A | 0.00072 | 0.03729 | 0.236 | 402.413 | 0.141 | 2 | 19820773 | 19820914 | 1 | 0 |  |  |
| CTBP2 | Variation_48450 | A | 0.00074 | 0.03766 | -0.15 | 239.888 | 1.308 | 10 | 126993292 | 126994600 | 0 |  |  |  |
| NDUFB7 | IgH3.2439 | D | 0.00074 | 0.03776 | 0.334 | 630.158 | 0.125 | 19 | 15171108 | 15171233 | 0 |  |  |  |
| KIAA1609 | IgH3.2259 | A | 0.00074 | 0.03783 | -0.309 | 637.32 | 5.745 | 16 | 82447181 | 82452926 | 0 |  |  |  |
| PSMB9 | IgH3.982 | A | 0.00074 | 0.0379 | 0.371 | 772.216 | 26.427 | 6 | 32173758 | 32200185 | 0 |  |  |  |
| JAG2 | CNVR6291.2 | A | 0.00074 | 0.0379 | -0.264 | 117.817 | 5.73 | 14 | 104813345 | 104819075 | 0 |  |  |  |
| HOXB8 | IgH3.2324 | A | 0.00075 | 0.03803 | -0.277 | 847.9425 | 0.151 | 17 | 44894021 | 44894172 | 0 |  |  |  |
| KIAA0182 | IgH3.2266 | D | 0.00075 | 0.03806 | 0.444 | 715.816 | 1.256 | 16 | 84951364 | 84952620 | 1 | 0 |  |  |
| COG4 | IgH3.2236 | D | 0.00075 | 0.03813 | -0.456 | 613.6315 | 0.043 | 16 | 69707118 | 69707161 | 0 |  | JPT | |
| PSMB9 | Variation_32788 | D | 0.00075 | 0.03826 | -0.168 | 469.299 | 8.577 | 6 | 32467750 | 32476327 | 0 |  |  |  |
| TIMM50 | Variation_30904 | A | 0.00076 | 0.03833 | -0.396 | 700.665 | 4.603 | 19 | 43969766 | 43974369 | 0 |  |  |  |
| RAD51AP1 | Variation_43856 | A | 0.00076 | 0.03839 | -0.413 | 562.4275 | 1.63 | 12 | 5092084 | 5093714 | 0 |  |  |  |
| C7orf25 | IgH3.1156 | A | 0.00076 | 0.03846 | -0.391 | 811.7295 | 0.609 | 7 | 42105380 | 42105989 | 1 | 0 |  |  |
| SRA1 | Variation_57007 | D | 0.00076 | 0.03849 | -0.236 | 235.8285 | 34.429 | 5 | 140166892 | 140201321 | 0 |  |  |  |
| NUTF2 | IgH3.2234 | D | 0.00076 | 0.03853 | 0.512 | 732.3755 | 2.356 | 16 | 67184073 | 67186429 | 1 | 0 |  |  |
| CCT3 | IgH3.104 | A | 0.00076 | 0.03862 | -0.494 | 654.04 | 22.842 | 1 | 155225558 | 155248400 | 1 | 1 |  |  |
| BCYRN1 | Variation_49412 | A | 0.00077 | 0.03869 | 0.356 | 795.348 | 8.194 | 14 | 74741204 | 74749398 | 0 |  |  |  |
| NDUFV1 | CNVR5211.1 | A | 0.00077 | 0.03875 | 0.32 | 56.4395 | 3.275 | 11 | 67191820 | 67195095 | 0 |  |  |  |
| TH1L | IgH3.2519 | A | 0.00077 | 0.03879 | 0.326 | 77.696 | 3.326 | 20 | 56920611 | 56923937 | 1 | 0 |  |  |
| VMAC | IgH3.2419 | A | 0.00077 | 0.03885 | -0.296 | 397.537 | 0.414 | 19 | 5461227 | 5461641 | 0 |  |  |  |
| ZNF483 | Variation_36907 | A | 0.00078 | 0.03914 | 0.159 | 954.5535 | 1.089 | 9 | 114308704 | 114309793 | 0 |  |  |  |
| CCDC78 | CNVR6591.1 | D | 0.00078 | 0.03921 | 0.592 | 486.528 | 29.513 | 16 | 1215813 | 1245326 | 0 |  |  |  |
| SFRS2IP | IgH3.1876 | D | 0.00079 | 0.03937 | -0.101 | 354.4825 | 1.911 | 12 | 44281397 | 44283308 | 0 |  |  |  |
| CACYBP | IgH3.121 | A | 0.00079 | 0.03953 | -0.277 | 53.651 | 0.836 | 1 | 173295719 | 173296555 | 1 | 1 |  |  |
| SKIV2L | IgH3.982 | A | 0.0008 | 0.03963 | 0.301 | 120.5095 | 26.427 | 6 | 32173758 | 32200185 | 0 |  |  |  |
| SAP30 | IgH3.789 | D | 0.0008 | 0.03972 | -0.361 | 591.682 | 0.731 | 4 | 173940646 | 173941377 | 1 | 1 |  |  |
| PBK | CNVR3837.1 | A | 0.00081 | 0.04004 | 0.388 | 300.0725 | 1.225 | 8 | 27437702 | 27438927 | 0 |  |  |  |
| FLAD1 | Variation_43741 | A | 0.00081 | 0.0401 | 0.508 | 881.683 | 3.779 | 1 | 152347532 | 152351311 | 0 |  |  |  |
| PCDH8 | CNVR5874.1 | D | 0.00081 | 0.0401 | 0.289 | 542.6825 | 11.417 | 13 | 51781468 | 51792885 | 0 |  |  |  |
| NOSIP | Variation_35661 | A | 0.00081 | 0.0401 | 0.382 | 122.287 | 12.864 | 19 | 54647342 | 54660206 | 0 |  |  |  |
| CCDC62 | CNVR5709.1 | D | 0.00081 | 0.04019 | -0.207 | 433.698 | 0.453 | 12 | 122285376 | 122285829 | 0 |  |  |  |
| GNAS | IgH3.2520 | D | 0.00082 | 0.04035 | 0.358 | 66.915 | 0.947 | 20 | 56957926 | 56958873 | 1 | 0 |  |  |
| TMEM141 | CNVR4543.1 | D | 0.00082 | 0.04048 | -0.223 | 585.763 | 0.981 | 9 | 139392847 | 139393828 | 0 |  |  |  |
| RAB14 | IgH3.1574 | A | 0.00082 | 0.04054 | 0.193 | 221.481 | 7.766 | 9 | 123217575 | 123225341 | 1 | 0 |  |  |
| SNRPD2 | CNVR7667.1 | A | 0.00083 | 0.0406 | -0.513 | 607.3995 | 6.466 | 19 | 50280751 | 50287217 | 0 |  |  |  |
| NLRP4 | IgH3.2478 | A | 0.00083 | 0.0406 | -0.198 | 105.218 | 0.601 | 19 | 60957476 | 60958077 | 0 |  |  |  |
| PPP1CA | CNVR5211.1 | A | 0.00083 | 0.04063 | 0.331 | 266.093 | 3.275 | 11 | 67191820 | 67195095 | 0 |  |  |  |
| MRPL41 | Variation_57995 | A | 0.00083 | 0.04066 | -0.355 | 324.8775 | 3.95 | 9 | 139893331 | 139897281 | 0 |  |  |  |
| C12orf57 | IgH3.1833 | A | 0.00083 | 0.04066 | 0.244 | 840.7675 | 171.34 | 12 | 7850883 | 8022225 | 0 |  |  |  |
| DYX1C1 | IgH3.2145 | A | 0.00083 | 0.04066 | -0.273 | 586.33 | 2.143 | 15 | 52963502 | 52965645 | 0 |  |  |  |
| OR7G2 | IgH3.2426 | D | 0.00083 | 0.04072 | -0.441 | 39.7665 | 0.225 | 19 | 9114342 | 9114567 | 1 | 1 |  |  |
| THAP11 | IgH3.2234 | D | 0.00083 | 0.04085 | 0.424 | 748.239 | 2.356 | 16 | 67184073 | 67186429 | 1 | 0 |  |  |
| HLA-DRB5 | IgH3.985 | A | 0.00083 | 0.04085 | 0.335 | 159.4045 | 194.94 | 6 | 32537621 | 32732557 | 0 |  | YRI | CHB|JPT|YRI |
| ACN9 | IgH3.1203 | A | 0.00084 | 0.04094 | -0.451 | 997.6955 | 13.272 | 7 | 97620757 | 97634029 | 1 | 1 | CHB|JPT|YRI | |
| C7orf29 | Variation_23122 | A | 0.00084 | 0.04094 | -0.359 | 891.767 | 7.063 | 7 | 150554605 | 150561668 | 0 |  | CEU | |
| LINGO3 | IgH3.2417 | A | 0.00084 | 0.04094 | -0.375 | 268.9135 | 0.77 | 19 | 2519263 | 2520033 | 0 |  |  |  |
| C6orf142 | Variation_36536 | D | 0.00084 | 0.04109 | -0.239 | 817.1965 | 20.758 | 6 | 54942930 | 54963688 | 0 |  |  |  |
| PDCD2L | IgH3.2456 | A | 0.00084 | 0.04109 | -0.202 | 747.896 | 6.632 | 19 | 40349239 | 40355871 | 0 |  |  |  |
| DBX1 | IgH3.1731 | A | 0.00085 | 0.04121 | 0.014 | 14.192 | 0.051 | 11 | 20150608 | 20150659 | 1 |  |  |  |
| TUB | IgH3.1725 | D | 0.00085 | 0.04131 | -0.255 | 295.1595 | 28.543 | 11 | 7769604 | 7798147 | 0 |  |  |  |
| VWC2L | CNVR1131.1 | A | 0.00085 | 0.04137 | -0.274 | 687.051 | 0.889 | 2 | 214380195 | 214381084 | 0 |  |  |  |
| TUBB2C | CNVR4543.1 | D | 0.00086 | 0.04161 | -0.263 | 135.601 | 0.981 | 9 | 139392847 | 139393828 | 0 |  |  |  |
| CDCA7 | CNVR1055.1 | D | 0.00086 | 0.04164 | -0.424 | 190.069 | 0.566 | 2 | 174125237 | 174125803 | 0 |  |  |  |
| TBCB | CNVR7629.1 | A | 0.00086 | 0.04164 | -0.327 | 123.3905 | 45.309 | 19 | 41449253 | 41494562 | 0 |  |  |  |
| IMPA2 | IgH3.2352 | D | 0.00086 | 0.04176 | -0.389 | 582.9745 | 0.713 | 18 | 11413547 | 11414260 | 1 | 1 |  |  |
| ZNF550 | CNVR7747.1 | D | 0.00086 | 0.04176 | -0.289 | 227.627 | 0.739 | 19 | 62527588 | 62528327 | 0 |  |  |  |
| CCT7 | IgH3.274 | A | 0.00088 | 0.04214 | -0.414 | 651.971 | 11.268 | 2 | 73981869 | 73993137 | 0 |  |  |  |
| MAT2B | IgH3.939 | A | 0.00088 | 0.04227 | 0.442 | 531.472 | 9.614 | 5 | 163407136 | 163416750 | 1 | 0 |  |  |
| PAAF1 | Variation_48880 | A | 0.00088 | 0.04227 | -0.213 | 65.6585 | 10.46 | 11 | 73361942 | 73372402 | 0 |  |  |  |
| C6orf167 | IgH3.1030 | A | 0.00088 | 0.04237 | -0.288 | 362.0235 | 0.267 | 6 | 98129422 | 98129689 | 1 | 1 |  |  |
| C14orf159 | IgH3.2095 | A | 0.00088 | 0.04237 | -0.399 | 715.294 | 0.527 | 14 | 91421340 | 91421867 | 1 | 1 |  |  |
| BHLHE22 | CNVR3914.1 | D | 0.00089 | 0.04246 | -0.354 | 595.973 | 2.757 | 8 | 66254405 | 66257162 | 0 |  |  |  |
| HIST1H3H | IgH3.975 | A | 0.00089 | 0.04266 | 0.384 | 1153.726 | 235.47 | 6 | 26850066 | 27085536 | 0 |  |  |  |
| C12orf24 | CNVR5667.1 | A | 0.00091 | 0.04323 | 0.401 | 781.183 | 2.945 | 12 | 108621884 | 108624829 | 0 |  |  |  |
| FOXD1 | Variation_57125 | A | 0.00091 | 0.04326 | -0.268 | 905.139 | 0.78 | 5 | 73684503 | 73685283 | 0 |  |  |  |
| LPAR2 | IgH3.2441 | D | 0.00091 | 0.04329 | -0.378 | 713.495 | 137.46 | 19 | 20379977 | 20517437 | 0 |  |  |  |
| KIAA1467 | IgH3.1842 | D | 0.00092 | 0.04342 | -0.19 | 683.972 | 6.661 | 12 | 12427474 | 12434135 | 0 |  |  |  |
| SLC25A24 | CNVR287.1 | D | 0.00092 | 0.04358 | 0.247 | 535.287 | 0.938 | 1 | 107976915 | 107977853 | 0 |  |  |  |
| PTPRJ | IgH3.1757 | D | 0.00092 | 0.04361 | -0.446 | 684.889 | 0.679 | 11 | 47350214 | 47350893 | 1 | 0 |  |  |
| HORMAD1 | IgH3.93 | D | 0.00093 | 0.04389 | -0.408 | 3565.637 | 1823 | 1 | 146294433 | 148117432 | 0 |  |  |  |
| SYNCRIP | CNVR2988.1 | A | 0.00093 | 0.04389 | 0.218 | 772.154 | 5.019 | 6 | 87165718 | 87170737 | 0 |  |  |  |
| CYC1 | CNVR4125.1 | A | 0.00093 | 0.04395 | 0.341 | 430.863 | 6.121 | 8 | 145657105 | 145663226 | 0 |  |  |  |
| C6orf162 | CNVR2988.1 | D | 0.00093 | 0.04399 | 0.41 | 935.6845 | 5.019 | 6 | 87165718 | 87170737 | 0 |  |  |  |
| KIRREL2 | IgH3.2458 | D | 0.00094 | 0.0442 | -0.397 | 468.777 | 17.187 | 19 | 41522139 | 41539326 | 0 |  |  |  |
| DIRAS2 | IgH3.1549 | D | 0.00094 | 0.0443 | -0.421 | 180.782 | 0.509 | 9 | 92609467 | 92609976 | 1 | 1 |  |  |
| SPRR4 | IgH3.100 | D | 0.00094 | 0.0443 | -0.152 | 71.9275 | 0.247 | 1 | 151138918 | 151139165 | 0 |  |  |  |
| UTP6 | IgH3.2305 | A | 0.00095 | 0.04457 | -0.339 | 803.5805 | 0.025 | 17 | 26430004 | 26430029 | 1 |  |  |  |
| RGPD2 | IgH3.291 | A | 0.00096 | 0.04473 | -0.348 | 622.2385 | 1063.5 | 2 | 86953419 | 88016919 | 0 |  |  |  |
| IMPA2 | IgH3.2354 | A | 0.00096 | 0.04479 | 0.303 | 104.32 | 0.086 | 18 | 11891888 | 11891974 | 0 |  |  |  |
| SCFD2 | IgH3.639 | A | 0.00097 | 0.045 | -0.518 | 1156.362 | 1.358 | 4 | 52524770 | 52526128 | 0 |  |  |  |
| ABCA12 | Variation_43421 | D | 0.00097 | 0.04509 | 0.296 | 118.883 | 2.153 | 2 | 215436976 | 215439129 | 0 |  |  |  |
| SLITRK1 | IgH3.2007 | D | 0.00097 | 0.04509 | -0.353 | 209.2995 | 0.043 | 13 | 83142658 | 83142701 | 0 |  |  |  |
| PPP2R3C | IgH3.2042 | D | 0.00097 | 0.04512 | -0.327 | 144.4905 | 0.751 | 14 | 34498734 | 34499485 | 0 |  |  |  |
| NLRP3 | IgH3.201 | A | 0.00098 | 0.04524 | -0.428 | 645.581 | 1.476 | 1 | 246309819 | 246311295 | 0 |  |  |  |
| RDH10 | IgH3.1428 | D | 0.00098 | 0.04533 | -0.404 | 402.266 | 4.384 | 8 | 73984869 | 73989253 | 1 | 1 |  |  |
| COX16 | IgH3.2070 | A | 0.00098 | 0.04548 | 0.307 | 342.0455 | 2.533 | 14 | 70222186 | 70224719 | 1 | 1 |  |  |
| HSPE1 | CNVR1101.1 | D | 0.00099 | 0.04569 | -0.311 | 291.354 | 0.52 | 2 | 197783604 | 197784124 | 0 |  |  |  |
| PTPLA | CNVR4632.1 | D | 0.001 | 0.04584 | 0.544 | 630.598 | 2.432 | 10 | 17056289 | 17058721 | 0 |  |  | CEU |
| TMEM48 | CNVR185.1 | A | 0.001 | 0.04599 | 0.414 | 144.2515 | 0.713 | 1 | 54184876 | 54185589 | 0 |  |  |  |
| C12orf10 | CNVR5535.1 | A | 0.001 | 0.04599 | 0.226 | 732.5555 | 0.983 | 12 | 51251420 | 51252403 | 0 |  |  |  |
| MAP1LC3B | IgH3.2270 | A | 0.001 | 0.04599 | -0.045 | 680.6975 | 1.728 | 16 | 86671152 | 86672880 | 0 |  |  |  |
| NSL1 | IgH3.169 | D | 0.00101 | 0.04629 | 0.476 | 450.4765 | 6.868 | 1 | 211452850 | 211459718 | 1 | 0 |  |  |
| SRR | IgH3.2276 | A | 0.00101 | 0.04629 | -0.349 | 100.0815 | 1.333 | 17 | 2065235 | 2066568 | 1 | 0 |  |  |
| OR4A47 | IgH3.1758 | D | 0.00101 | 0.04629 | 0.181 | 89.992 | 1.242 | 11 | 48557998 | 48559240 | 0 |  |  |  |
| COASY | IgH3.2315 | A | 0.00101 | 0.04629 | -0.186 | 358.9425 | 3.93 | 17 | 38330626 | 38334556 | 0 |  |  |  |
| IFI35 | IgH3.2315 | A | 0.00102 | 0.04661 | -0.226 | 87.4865 | 3.93 | 17 | 38330626 | 38334556 | 0 |  |  |  |
| IKZF2 | IgH3.373 | A | 0.00103 | 0.04693 | 0.329 | 349.1565 | 1.019 | 2 | 213997645 | 213998664 | 0 |  |  |  |
| TMEM14B | CNVR2776.1 | A | 0.00105 | 0.04756 | 0.347 | 13.527 | 4.064 | 6 | 10849095 | 10853159 | 0 |  |  |  |
| CA1 | Variation_33047 | A | 0.00107 | 0.04818 | -0.272 | 567.0175 | 2.728 | 8 | 87021033 | 87023761 | 0 |  |  |  |
| KLF6 | IgH3.1597 | A | 0.00107 | 0.04818 | -0.249 | 973.8985 | 0.654 | 10 | 2839259 | 2839913 | 0 |  |  |  |
| UNG | CNVR5667.1 | A | 0.00108 | 0.04849 | 0.544 | 593.9225 | 2.945 | 12 | 108621884 | 108624829 | 0 |  |  |  |
| PYCRL | CNVR4125.1 | A | 0.00108 | 0.04849 | 0.384 | 893.9785 | 6.121 | 8 | 145657105 | 145663226 | 0 |  |  |  |
| HSP90AA1 | Variation_55135 | A | 0.00108 | 0.04849 | 0.359 | 27.272 | 0.446 | 14 | 101592997 | 101593443 | 0 |  |  |  |
| HIST1H2BD | IgH3.975 | A | 0.00109 | 0.0488 | 0.415 | 465.7425 | 235.47 | 6 | 26850066 | 27085536 | 0 |  |  |  |
| SPR | Variation_30952 | D | 0.00109 | 0.0488 | 0.432 | 720.883 | 1.788 | 2 | 73692185 | 73693973 | 0 |  |  |  |
| AUP1 | Variation_30952 | A | 0.00109 | 0.0488 | 0.352 | 917.591 | 1.788 | 2 | 73692185 | 73693973 | 0 |  |  |  |
| MUC20 | IgH3.565 | D | 0.00109 | 0.0488 | -0.394 | 829.898 | 7.892 | 3 | 196115865 | 196123757 | 0 |  | CHB | |
| SSBP1 | IgH3.1239 | D | 0.00109 | 0.0488 | -0.482 | 496.1605 | 8.483 | 7 | 141591087 | 141599570 | 0 |  |  |  |
| BFAR | IgH3.2207 | A | 0.0011 | 0.0491 | 0.356 | 500.136 | 3.599 | 16 | 14154044 | 14157643 | 1 | 0 |  |  |
| CLEC3A | IgH3.2245 | A | 0.0011 | 0.0491 | 0.399 | 916.9175 | 0.279 | 16 | 77535778 | 77536057 | 0 |  |  |  |
| TBL1XR1 | Variation_32537 | A | 0.00111 | 0.04941 | 0.213 | 841.503 | 4.281 | 3 | 177470126 | 177474407 | 0 |  |  |  |
| SMCR8 | CNVR7015.1 | A | 0.00111 | 0.04941 | 0.13 | 293.076 | 21.625 | 17 | 18469595 | 18491220 | 0 |  |  |  |
| PIH1D1 | Variation_35661 | A | 0.00112 | 0.04971 | 0.45 | 3.234 | 12.864 | 19 | 54647342 | 54660206 | 0 |  |  |  |
| SERPINA1 | IgH3.2097 | D | 0.00113 | 0.05001 | 0.358 | 530.1855 | 1.759 | 14 | 94450874 | 94452633 | 1 | 1 |  |  |
| C14orf156 | IgH3.2077 | A | 0.00113 | 0.05001 | -0.406 | 601.1165 | 0.2 | 14 | 76647920 | 76648120 | 0 |  |  |  |
| TNIP1 | CNVR2648.1 | D | 0.00113 | 0.05001 | 0.491 | 353.112 | 2.88 | 5 | 150769996 | 150772876 | 0 |  |  |  |
| IRF2BP2 | IgH3.185 | A | 0.00114 | 0.05031 | -0.383 | 848.9305 | 11.358 | 1 | 233663875 | 233675233 | 0 |  |  |  |
| KIAA1244 | IgH3.1065 | A | 0.00114 | 0.05031 | 0.395 | 1030.677 | 0.43 | 6 | 139647008 | 139647438 | 0 |  |  |  |
| CALR | IgH3.2433 | A | 0.00114 | 0.05031 | -0.392 | 443.811 | 0.873 | 19 | 12469984 | 12470857 | 0 |  |  |  |
| LRRN2 | IgH3.162 | A | 0.00115 | 0.05061 | 0.274 | 67.202 | 2.449 | 1 | 202955499 | 202957948 | 1 | 1 |  |  |
| AP2S1 | CNVR7674.1 | D | 0.00116 | 0.0509 | -0.341 | 561.236 | 6.117 | 19 | 51481475 | 51487592 | 0 |  |  |  |
| PIPOX | IgH3.2304 | D | 0.00116 | 0.0509 | -0.413 | 596.2355 | 0.978 | 17 | 23805456 | 23806434 | 0 |  |  |  |
| KCNJ14 | IgH3.2463 | D | 0.00116 | 0.0509 | 0.41 | 217.749 | 0.082 | 19 | 53441040 | 53441122 | 0 |  |  |  |
| ZNF709 | IgH3.2433 | A | 0.00117 | 0.0512 | -0.523 | 10.2145 | 0.873 | 19 | 12469984 | 12470857 | 0 |  |  |  |
| LRRC37B | Variation_30822 | A | 0.00118 | 0.05149 | -0.197 | 12.503 | 23.703 | 17 | 27412804 | 27436507 | 0 |  |  |  |
| TMEM121 | IgH3.2106 | A | 0.00119 | 0.05178 | -0.012 | 655.425 | 1297.4 | 14 | 105059071 | 106356482 | 0 |  |  |  |
| GLRX5 | CNVR6237.1 | A | 0.00119 | 0.05178 | -0.361 | 75.967 | 4.143 | 14 | 95153980 | 95158123 | 0 |  |  |  |
| B3GAT2 | IgH3.1010 | D | 0.00121 | 0.05235 | -0.372 | 4.559 | 17.79 | 6 | 71689103 | 71706893 | 1 | 1 |  |  |
| TRIM32 | IgH3.1571 | D | 0.00121 | 0.05235 | -0.462 | 90.4035 | 0.656 | 9 | 118587132 | 118587788 | 1 | 0 |  |  |
| LCE3D | IgH3.99 | D | 0.00121 | 0.05235 | -0.486 | 270.896 | 7.037 | 1 | 151093458 | 151100495 | 0 |  |  |  |
| C20orf72 | IgH3.2501 | D | 0.00121 | 0.05235 | 0.388 | 304.4575 | 4.94 | 20 | 18215689 | 18220629 | 0 |  |  |  |
| DNAJB1 | IgH3.2436 | A | 0.00122 | 0.05264 | 0.258 | 347.6215 | 2.181 | 19 | 14837103 | 14839284 | 1 | 1 |  |  |
| FANCD2 | CNVR1283.1 | A | 0.00122 | 0.05264 | 0.326 | 544.5025 | 1.755 | 3 | 9536103 | 9537858 | 0 |  |  |  |
| C7orf11 | IgH3.1155 | A | 0.00122 | 0.05264 | -0.404 | 32.7235 | 0.088 | 7 | 40107142 | 40107230 | 0 |  |  |  |
| PLEKHG2 | Variation_30904 | A | 0.00122 | 0.05264 | 0.374 | 635.7775 | 4.603 | 19 | 43969766 | 43974369 | 0 |  |  |  |
| UBB | IgH3.2298 | A | 0.00124 | 0.05321 | 0.412 | 559.434 | 1.646 | 17 | 16786192 | 16787838 | 1 | 0 |  |  |
| PLCD1 | IgH3.427 | A | 0.00124 | 0.05321 | -0.414 | 88.479 | 10.216 | 3 | 37951704 | 37961920 | 0 |  |  |  |
| GAS1 | IgH3.1548 | A | 0.00124 | 0.05321 | -0.401 | 932.524 | 40.662 | 9 | 89703365 | 89744027 | 0 |  |  |  |
| RNF44 | CNVR2697.1 | A | 0.00124 | 0.05321 | -0.289 | 574.5055 | 1.049 | 5 | 176466696 | 176467745 | 0 |  |  |  |
| ERG | Variation_56332 | A | 0.00124 | 0.05321 | -0.313 | 302.971 | 0.857 | 21 | 38512155 | 38513012 | 0 |  |  |  |
| PINX1 | IgH3.1372 | A | 0.00125 | 0.05349 | -0.396 | 346.864 | 6.326 | 8 | 11047528 | 11053854 | 1 | 1 |  |  |
| RASSF9 | CNVR5611.1 | A | 0.00125 | 0.05349 | 0.042 | 207.899 | 7.742 | 12 | 84950225 | 84957967 | 0 |  |  |  |
| LENG8 | Variation_33674 | A | 0.00125 | 0.05349 | -0.143 | 72.6975 | 0.859 | 19 | 59586174 | 59587033 | 0 |  |  |  |
| PQLC2 | IgH3.21 | A | 0.00126 | 0.05377 | -0.395 | 843.181 | 0.039 | 1 | 18676692 | 18676731 | 1 | 1 |  |  |
| TKTL2 | IgH3.778 | A | 0.00126 | 0.05377 | -0.366 | 676.9155 | 0.896 | 4 | 163936629 | 163937525 | 1 | 1 |  |  |
| DHCR24 | CNVR185.1 | A | 0.00126 | 0.05377 | 0.383 | 922.1785 | 0.713 | 1 | 54184876 | 54185589 | 0 |  |  |  |
| PTH2R | Variation_50385 | A | 0.00126 | 0.05377 | 0.155 | 599.167 | 1.038 | 2 | 208424990 | 208426028 | 0 |  |  |  |
| PLXNC1 | CNVR5627.1 | D | 0.00126 | 0.05377 | -0.351 | 179.3835 | 3.92 | 12 | 92967569 | 92971489 | 0 |  |  |  |
| CSNK2A2 | IgH3.2230 | D | 0.00126 | 0.05377 | 0.296 | 735.2885 | 0.756 | 16 | 57504964 | 57505720 | 0 |  |  |  |
| SLMO2 | IgH3.2519 | A | 0.00127 | 0.05404 | 0.385 | 127.497 | 3.326 | 20 | 56920611 | 56923937 | 1 | 0 |  |  |
| C2orf34 | IgH3.256 | A | 0.00127 | 0.05404 | -0.349 | 1015.679 | 2.774 | 2 | 43633598 | 43636372 | 0 |  |  |  |
| CTDSPL | IgH3.428 | D | 0.00128 | 0.05432 | 0.05 | 284.06 | 0.708 | 3 | 38224232 | 38224940 | 1 | 1 |  |  |
| ANGEL1 | IgH3.2077 | A | 0.00128 | 0.05432 | -0.356 | 311.633 | 0.2 | 14 | 76647920 | 76648120 | 0 |  |  |  |
| SNORD24 | IgH3.1584 | D | 0.00128 | 0.05432 | 0.277 | 441.0075 | 0.202 | 9 | 135647217 | 135647419 | 0 |  |  |  |
| NTAN1 | IgH3.2207 | A | 0.00129 | 0.05459 | 0.448 | 896.03 | 3.599 | 16 | 14154044 | 14157643 | 1 | 0 |  |  |
| RPUSD3 | IgH3.407 | A | 0.0013 | 0.05487 | -0.552 | 926.63 | 0.287 | 3 | 8931131 | 8931418 | 1 | 0 |  |  |
| C3orf57 | IgH3.527 | D | 0.0013 | 0.05487 | 0.448 | 439.379 | 0.022 | 3 | 162998309 | 162998331 | 0 |  |  |  |
| KIAA1530 | IgH3.572 | A | 0.0013 | 0.05487 | 0.414 | 390.8725 | 0.317 | 4 | 960756 | 961073 | 0 |  |  |  |
| FXYD7 | IgH3.2457 | A | 0.0013 | 0.05487 | -0.315 | 200.7475 | 17.565 | 19 | 40541049 | 40558614 | 0 |  |  |  |
| RRM2 | CNVR678.1 | A | 0.00131 | 0.05514 | 0.557 | 120.216 | 7.132 | 2 | 10068005 | 10075137 | 0 |  |  |  |
| C19orf55 | CNVR7629.1 | A | 0.00131 | 0.05514 | 0.317 | 480.1985 | 45.309 | 19 | 41449253 | 41494562 | 0 |  |  |  |
| TMEM147 | IgH3.2456 | D | 0.00131 | 0.05514 | -0.116 | 383.403 | 6.632 | 19 | 40349239 | 40355871 | 0 |  |  |  |
| BTNL9 | IgH3.953 | D | 0.00132 | 0.05542 | 0.356 | 320.8975 | 192.94 | 5 | 180186049 | 180378984 | 0 |  |  |  |
| DGKG | IgH3.554 | D | 0.00132 | 0.05542 | -0.341 | 728.276 | 0.086 | 3 | 188183519 | 188183605 | 0 |  |  |  |
| KIAA1530 | CNVR1749.1 | A | 0.00133 | 0.05571 | -0.337 | 73.656 | 2.82 | 4 | 1279224 | 1282044 | 0 |  |  |  |
| RBMS2 | CNVR5552.1 | A | 0.00134 | 0.05599 | -0.378 | 354.189 | 48.398 | 12 | 55617449 | 55665847 | 0 |  | CEU|CHB|JPT | |
| SNORA46 | CNVR6743.1 | D | 0.00134 | 0.05599 | 0.113 | 120.0165 | 0.614 | 16 | 57020261 | 57020875 | 0 |  |  |  |
| CGREF1 | IgH3.236 | D | 0.00136 | 0.05655 | -0.236 | 871.862 | 3.335 | 2 | 28055129 | 28058464 | 0 |  |  |  |
| SNORD116-3 | IgH3.2113 | D | 0.00136 | 0.05655 | 0.323 | 1180.656 | 438.32 | 15 | 21891651 | 22329972 | 0 |  |  |  |
| POP4 | IgH3.2454 | D | 0.00137 | 0.05683 | 0.378 | 965.126 | 0.161 | 19 | 35759712 | 35759873 | 1 | 1 |  |  |
| PAPSS1 | IgH3.707 | A | 0.00137 | 0.05683 | -0.397 | 421.5145 | 0.041 | 4 | 109229104 | 109229145 | 1 |  |  |  |
| C1orf212 | CNVR139.1 | A | 0.00138 | 0.05711 | 0.265 | 139.4995 | 2.044 | 1 | 34954800 | 34956844 | 0 |  |  |  |
| CAMK1D | IgH3.1614 | D | 0.00139 | 0.05739 | -0.347 | 425.5335 | 2.29 | 10 | 13096544 | 13098834 | 0 |  |  |  |
| AHDC1 | IgH3.25 | A | 0.0014 | 0.05766 | -0.48 | 904.235 | 15.972 | 1 | 26871787 | 26887759 | 1 | 1 |  |  |
| MLLT4 | CNVR3183.1 | A | 0.0014 | 0.05766 | -0.372 | 719.18 | 4.944 | 6 | 168754807 | 168759751 | 0 |  |  |  |
| OBFC2B | Variation_43857 | A | 0.0014 | 0.05766 | -0.449 | 619.5935 | 1.972 | 12 | 54288540 | 54290512 | 0 |  |  |  |
| TMED10 | Variation_49412 | A | 0.0014 | 0.05766 | 0.391 | 46.5945 | 8.194 | 14 | 74741204 | 74749398 | 0 |  |  |  |
| WDR5 | CNVR4519.1 | A | 0.0014 | 0.05766 | -0.314 | 489.384 | 3.275 | 9 | 136495846 | 136499121 | 0 |  |  |  |
| WDR90 | CNVR6591.1 | A | 0.0014 | 0.05766 | 0.487 | 552.46 | 29.513 | 16 | 1215813 | 1245326 | 0 |  |  |  |
| COX19 | IgH3.1101 | D | 0.00141 | 0.05794 | -0.398 | 125.3455 | 0.379 | 7 | 851230 | 851609 | 1 | 1 |  |  |
| FBXO6 | CNVR70.1 | A | 0.00141 | 0.05794 | -0.325 | 258.782 | 3.616 | 1 | 11912455 | 11916071 | 0 |  |  |  |
| MAP4K1 | Variation_30904 | A | 0.00141 | 0.05794 | -0.373 | 182.163 | 4.603 | 19 | 43969766 | 43974369 | 0 |  |  |  |
| PLA2G1B | Variation_38248 | D | 0.00142 | 0.05821 | -0.026 | 771.915 | 2.557 | 12 | 120020329 | 120022886 | 0 |  |  |  |
| MRPL3 | CNVR1542.1 | D | 0.00144 | 0.05875 | -0.407 | 472.258 | 1.671 | 3 | 133157220 | 133158891 | 0 |  |  |  |
| NDUFA11 | CNVR7510.1 | D | 0.00144 | 0.05875 | 0.254 | 823.636 | 0.75 | 19 | 5027094 | 5027844 | 0 |  |  |  |
| ABCB10 | IgH3.181 | D | 0.00145 | 0.05902 | 0.342 | 527.4415 | 0.203 | 1 | 228267551 | 228267754 | 1 | 0 |  |  |
| ROMO1 | Variation_50650 | A | 0.00145 | 0.05902 | 0.457 | 147.3385 | 8.036 | 20 | 33608160 | 33616196 | 0 |  |  |  |
| C5orf32 | Variation_57007 | D | 0.00146 | 0.05929 | -0.043 | 580.4805 | 34.429 | 5 | 140166892 | 140201321 | 0 |  |  |  |
| ACTL6A | IgH3.547 | D | 0.00147 | 0.05955 | 0.326 | 494.8225 | 8.623 | 3 | 181275278 | 181283901 | 1 | 0 |  |  |
| USP39 | IgH3.289 | A | 0.00147 | 0.05955 | 0.384 | 447.738 | 0.084 | 2 | 86161135 | 86161219 | 0 |  |  |  |
| PSMD8 | Variation_30904 | A | 0.00147 | 0.05955 | -0.328 | 405.798 | 4.603 | 19 | 43969766 | 43974369 | 0 |  |  |  |
| S100A7L2 | IgH3.100 | D | 0.00148 | 0.05982 | -0.167 | 538.816 | 0.247 | 1 | 151138918 | 151139165 | 0 |  |  |  |
| ADAMDEC1 | IgH3.1398 | D | 0.00148 | 0.05982 | -0.302 | 110.0615 | 5.636 | 8 | 24201363 | 24206999 | 0 |  |  |  |
| TRHR | Variation_57741 | A | 0.00148 | 0.05982 | -0.119 | 393.877 | 0.821 | 8 | 110579231 | 110580052 | 0 |  |  |  |
| FBXL4 | IgH3.1031 | A | 0.00149 | 0.06008 | 0.342 | 1027.031 | 2.33 | 6 | 98439580 | 98441910 | 1 | 0 |  |  |
| RTN2 | CNVR7674.1 | D | 0.00149 | 0.06008 | -0.319 | 792.1465 | 6.117 | 19 | 51481475 | 51487592 | 0 |  |  |  |
| OR10J1 | Variation_54102 | A | 0.00151 | 0.0606 | -0.184 | 237.6805 | 1.443 | 1 | 157915081 | 157916524 | 0 |  |  |  |
| MYEOV2 | CNVR1208.1 | A | 0.00151 | 0.0606 | -0.305 | 400.3005 | 4.671 | 2 | 240323702 | 240328373 | 0 |  |  |  |
| RBM33 | IgH3.1273 | A | 0.00151 | 0.0606 | 0.471 | 605.337 | 3.289 | 7 | 154594759 | 154598048 | 0 |  |  |  |
| ELF2 | IgH3.750 | A | 0.00152 | 0.06086 | 0.061 | 21.315 | 0.01 | 4 | 140217878 | 140217888 | 1 | 1 |  |  |
| WIZ | IgH3.2439 | D | 0.00153 | 0.06112 | -0.333 | 236.494 | 0.125 | 19 | 15171108 | 15171233 | 0 |  |  |  |
| SNRNP70 | IgH3.2463 | D | 0.00154 | 0.06138 | -0.407 | 850.98 | 0.082 | 19 | 53441040 | 53441122 | 0 |  |  |  |
| B3GNT8 | CNVR7646.1 | A | 0.00155 | 0.06164 | -0.058 | 243.8315 | 5.567 | 19 | 46383741 | 46389308 | 0 |  |  |  |
| ARHGDIB | Variation_22705 | A | 0.00155 | 0.06164 | -0.298 | 907.315 | 6.845 | 12 | 15906760 | 15913605 | 0 |  |  |  |
| IDH3A | IgH3.2162 | D | 0.00156 | 0.06189 | -0.459 | 56.4345 | 3.959 | 15 | 76184901 | 76188860 | 1 | 0 |  |  |
| DMRTA1 | Variation_58005 | D | 0.00156 | 0.06189 | -0.189 | 678.5165 | 0.17 | 9 | 23118257 | 23118427 | 0 |  |  |  |
| PSME2 | Variation_49328 | A | 0.00156 | 0.06189 | -0.123 | 176.75 | 5.596 | 14 | 23863602 | 23869198 | 0 |  |  |  |
| FUT5 | CNVR7510.1 | D | 0.00156 | 0.06189 | 0.263 | 792.4745 | 0.75 | 19 | 5027094 | 5027844 | 0 |  |  |  |
| FAM98B | IgH3.2129 | A | 0.00157 | 0.06215 | -0.357 | 653.6215 | 1.127 | 15 | 35895929 | 35897056 | 1 | 1 |  |  |
| LARS2 | CNVR1371.1 | A | 0.00157 | 0.06215 | -0.284 | 28.6975 | 8.777 | 3 | 45518291 | 45527068 | 0 |  |  |  |
| GRXCR1 | Variation_38555 | D | 0.00157 | 0.06215 | -0.118 | 773.583 | 2.872 | 4 | 43433755 | 43436627 | 0 |  |  |  |
| CBX4 | Variation_30854 | A | 0.00157 | 0.06215 | 0.365 | 275.484 | 3.769 | 17 | 75702047 | 75705816 | 0 |  |  |  |
| SFTPD | CNVR4815.2 | A | 0.00158 | 0.0624 | -0.33 | 613.5065 | 98.077 | 10 | 81128690 | 81226767 | 0 |  |  |  |
| GSS | Variation_50650 | A | 0.00158 | 0.0624 | 0.368 | 610.563 | 8.036 | 20 | 33608160 | 33616196 | 0 |  |  |  |
| ZNF160 | CNVR7705.2 | A | 0.00158 | 0.0624 | 0.453 | 726.3 | 1.465 | 19 | 57554521 | 57555986 | 0 |  | YRI | |
| OSTC | IgH3.707 | A | 0.00159 | 0.06265 | -0.401 | 570.7255 | 0.041 | 4 | 109229104 | 109229145 | 1 |  |  |  |
| CNPY2 | IgH3.1880 | A | 0.00159 | 0.06265 | 0.465 | 720.0505 | 6.611 | 12 | 54276690 | 54283301 | 0 |  |  |  |
| PAX9 | IgH3.2045 | A | 0.0016 | 0.0629 | -0.36 | 652.136 | 0.451 | 14 | 35554731 | 35555182 | 1 | 0 |  |  |
| SEC11C | IgH3.2380 | D | 0.0016 | 0.0629 | -0.391 | 314.81 | 0.659 | 18 | 55282713 | 55283372 | 1 | 0 |  |  |
| H2AFY | IgH3.922 | D | 0.00161 | 0.06315 | -0.412 | 80.1095 | 0.873 | 5 | 134650725 | 134651598 | 1 | 1 |  |  |
| IRF3 | Variation_35661 | A | 0.00161 | 0.06315 | 0.435 | 216.882 | 12.864 | 19 | 54647342 | 54660206 | 0 |  |  |  |
| ZNF517 | CNVR4125.1 | A | 0.00162 | 0.0634 | -0.335 | 346.154 | 6.121 | 8 | 145657105 | 145663226 | 0 |  |  |  |
| OR52N4 | IgH3.1718 | A | 0.00162 | 0.0634 | 0.365 | 858.4025 | 60.245 | 11 | 4904736 | 4964981 | 0 |  |  |  |
| NUP210 | CNVR1299.1 | A | 0.00163 | 0.06365 | -0.363 | 270.8675 | 1.092 | 3 | 13656186 | 13657278 | 0 |  |  |  |
| ILVBL | CNVR7552.1 | A | 0.00164 | 0.06389 | -0.441 | 530.2325 | 1.234 | 19 | 15623030 | 15624264 | 0 |  |  |  |
| WDR4 | CNVR8020.1 | A | 0.00164 | 0.06389 | 0.383 | 997.486 | 1.075 | 21 | 42157561 | 42158636 | 0 |  | CHB | |
| PRDM15 | CNVR8020.1 | D | 0.00164 | 0.06389 | -0.315 | 24.9715 | 1.075 | 21 | 42157561 | 42158636 | 0 |  |  |  |
| RNF24 | IgH3.2487 | D | 0.00164 | 0.06389 | -0.198 | 489.0165 | 5.919 | 20 | 4394037 | 4399956 | 0 |  |  |  |
| JUN | IgH3.40 | A | 0.00165 | 0.06414 | 0.302 | 798.1395 | 0.09 | 1 | 58222617 | 58222707 | 1 | 0 |  |  |
| ITLN2 | CNVR382.1 | A | 0.00165 | 0.06414 | 0.085 | 30.6065 | 6.867 | 1 | 159220366 | 159227233 | 0 |  |  |  |
| CENPP | IgH3.1550 | A | 0.00167 | 0.06464 | 0.051 | 864.769 | 2.145 | 9 | 93408713 | 93410858 | 1 | 1 |  |  |
| MICB | IgH3.982 | A | 0.00168 | 0.06489 | 0.294 | 580.188 | 26.427 | 6 | 32173758 | 32200185 | 0 |  |  |  |
| HLA-DQA2 | IgH3.984 | D | 0.00169 | 0.06514 | 0.445 | 323.696 | 6.987 | 6 | 32499966 | 32506953 | 0 |  | CEU|CHB | CEU|CHB|JPT |
| C7orf46 | IgH3.1145 | A | 0.00169 | 0.06514 | 0.347 | 579.4795 | 0.914 | 7 | 24277470 | 24278384 | 0 |  |  |  |
| TINF2 | Variation_49328 | A | 0.00169 | 0.06514 | -0.254 | 80.599 | 5.596 | 14 | 23863602 | 23869198 | 0 |  |  |  |
| PEX26 | IgH3.2570 | D | 0.00169 | 0.06514 | 0.36 | 37.8265 | 288.31 | 22 | 17128428 | 17416739 | 0 |  |  |  |
| ZNF610 | IgH3.2468 | D | 0.0017 | 0.06539 | -0.391 | 739.5615 | 23.669 | 19 | 56823613 | 56847282 | 0 |  |  |  |
| VAV2 | IgH3.1584 | D | 0.0017 | 0.06539 | -0.361 | 84.936 | 0.202 | 9 | 135647217 | 135647419 | 0 |  |  |  |
| CACNG6 | IgH3.2471 | D | 0.0017 | 0.06539 | 0.337 | 1003.719 | 33.47 | 19 | 58210559 | 58244029 | 0 |  |  |  |
| OR5B12 | IgH3.1764 | D | 0.00171 | 0.06564 | 0.397 | 582.2325 | 43.778 | 11 | 58567817 | 58611595 | 0 |  |  |  |
| GPR32 | Variation_50197 | A | 0.00171 | 0.06564 | -0.142 | 53.836 | 5.407 | 19 | 56022744 | 56028151 | 0 |  |  |  |
| ABR | CNVR6916.1 | D | 0.00172 | 0.06589 | -0.426 | 491.6705 | 20.927 | 17 | 425084 | 446011 | 0 |  |  |  |
| ATP5SL | CNVR7646.1 | A | 0.00172 | 0.06589 | -0.307 | 252.4155 | 5.567 | 19 | 46383741 | 46389308 | 0 |  |  |  |
| RBKS | IgH3.233 | A | 0.00173 | 0.06613 | -0.338 | 910.3315 | 4.957 | 2 | 27004395 | 27009352 | 1 | 1 |  |  |
| C6orf27 | IgH3.984 | D | 0.00173 | 0.06613 | -0.392 | 649.2545 | 6.987 | 6 | 32499966 | 32506953 | 0 |  |  |  |
| KRT39 | IgH3.2314 | A | 0.00175 | 0.06663 | -0.054 | 614.4525 | 0.189 | 17 | 36986979 | 36987168 | 0 |  |  |  |
| SLC9A1 | IgH3.25 | A | 0.00177 | 0.06712 | -0.387 | 462.1615 | 15.972 | 1 | 26871787 | 26887759 | 1 | 1 |  |  |
| TMEM126A | IgH3.1781 | A | 0.00177 | 0.06712 | 0.393 | 844.734 | 0.569 | 11 | 85885982 | 85886551 | 1 | 0 |  |  |
| LOC286367 | IgH3.1558 | A | 0.00177 | 0.06712 | -0.107 | 969.9515 | 0.594 | 9 | 105608505 | 105609099 | 0 |  |  |  |
| HN1 | Variation_49925 | D | 0.00177 | 0.06712 | -0.301 | 311.107 | 14.121 | 17 | 70348607 | 70362728 | 0 |  |  |  |
| MCCC2 | IgH3.880 | A | 0.00178 | 0.06736 | 0.44 | 53.6765 | 1.065 | 5 | 71008787 | 71009852 | 1 | 1 |  |  |
| MAEA | CNVR1749.1 | A | 0.00179 | 0.06761 | 0.383 | 20.984 | 2.82 | 4 | 1279224 | 1282044 | 0 |  |  |  |
| RBBP6 | Variation_35367 | A | 0.00179 | 0.06761 | -0.394 | 793.373 | 8.139 | 16 | 25260758 | 25268897 | 0 |  |  |  |
| YIPF3 | IgH3.990 | A | 0.00181 | 0.06809 | 0.231 | 443.5925 | 0.357 | 6 | 43146697 | 43147054 | 1 | 0 |  |  |
| HLA-DQA2 | IgH3.985 | A | 0.00182 | 0.06833 | -0.191 | 380.0155 | 194.94 | 6 | 32537621 | 32732557 | 0 |  | CEU|CHB | CEU|CHB|JPT |
| EMG1 | IgH3.1833 | D | 0.00183 | 0.06857 | 0.264 | 812.408 | 171.34 | 12 | 7850883 | 8022225 | 0 |  |  |  |
| CBLN2 | IgH3.2404 | A | 0.00184 | 0.06881 | -0.181 | 740.337 | 0.047 | 18 | 69099159 | 69099206 | 1 | 0 |  |  |
| CBX3 | IgH3.1147 | A | 0.00184 | 0.06881 | 0.306 | 688.1785 | 0.03 | 7 | 25525511 | 25525541 | 1 |  |  |  |
| NCOA6 | Variation_50650 | A | 0.00184 | 0.06881 | -0.373 | 782.476 | 8.036 | 20 | 33608160 | 33616196 | 0 |  |  |  |
| MYBL2 | CNVR7851.1 | D | 0.00184 | 0.06881 | -0.269 | 572.43 | 1.118 | 20 | 42326818 | 42327936 | 0 |  | CHB|JPT | |
| LGALS4 | CNVR7633.1 | D | 0.00184 | 0.06881 | -0.267 | 958.376 | 3.806 | 19 | 43033392 | 43037198 | 0 |  |  |  |
| OGG1 | IgH3.407 | A | 0.00185 | 0.06905 | -0.485 | 839.3695 | 0.287 | 3 | 8931131 | 8931418 | 1 | 0 |  |  |
| OR7D2 | IgH3.2425 | D | 0.00185 | 0.06905 | 0.129 | 456.197 | 0.082 | 19 | 8702725 | 8702807 | 0 |  |  |  |
| ANXA11 | IgH3.1683 | D | 0.00187 | 0.06952 | -0.41 | 395.369 | 34.881 | 10 | 81552155 | 81587036 | 0 |  |  |  |
| PEX1 | IgH3.1195 | A | 0.00187 | 0.06952 | 0.342 | 781.0485 | 2.358 | 7 | 91195157 | 91197515 | 0 |  |  |  |
| METT11D1 | IgH3.2031 | D | 0.00187 | 0.06952 | -0.385 | 993.8825 | 0.305 | 14 | 21525454 | 21525759 | 0 |  |  |  |
| OR10G7 | IgH3.1805 | D | 0.00188 | 0.06976 | -0.252 | 866.0195 | 1.431 | 11 | 124281185 | 124282616 | 1 | 1 |  |  |
| WNT1 | IgH3.1877 | D | 0.00188 | 0.06976 | -0.39 | 649.176 | 0.176 | 12 | 47011494 | 47011670 | 0 |  |  |  |
| LRRC14 | Variation_41992 | A | 0.00189 | 0.06999 | -0.412 | 445.637 | 0.425 | 8 | 146163631 | 146164056 | 0 |  |  |  |
| ENAH | CNVR523.1 | A | 0.0019 | 0.07022 | -0.575 | 614.082 | 8.158 | 1 | 224442473 | 224450631 | 0 |  |  |  |
| KLHDC8A | CNVR480.1 | A | 0.00191 | 0.07046 | -0.274 | 864.3685 | 5.315 | 1 | 204449492 | 204454807 | 0 |  |  |  |
| C17orf53 | IgH3.2319 | D | 0.00191 | 0.07046 | 0.358 | 999.4145 | 1.166 | 17 | 40585082 | 40586248 | 0 |  |  |  |
| NAPSA | Variation_35661 | A | 0.00191 | 0.07046 | 0.313 | 916.234 | 12.864 | 19 | 54647342 | 54660206 | 0 |  |  |  |
| LMF1 | Variation_49688 | D | 0.00191 | 0.07046 | 0.319 | 603.698 | 2.685 | 16 | 299954 | 302639 | 0 |  |  |  |
| COL5A3 | CNVR7533.1 | A | 0.00192 | 0.07069 | -0.339 | 115.364 | 2.239 | 19 | 10073175 | 10075414 | 0 |  |  |  |
| KCNQ2 | IgH3.2527 | D | 0.00192 | 0.07069 | -0.358 | 883.4 | 1.316 | 20 | 62425269 | 62426585 | 0 |  |  |  |
| BAT2 | IgH3.982 | D | 0.00193 | 0.07092 | -0.336 | 455.564 | 26.427 | 6 | 32173758 | 32200185 | 0 |  |  |  |
| ALOX15B | Variation_35517 | A | 0.00193 | 0.07092 | -0.109 | 360.9655 | 5.201 | 17 | 8251695 | 8256896 | 0 |  |  |  |
| CHD8 | IgH3.2031 | A | 0.00193 | 0.07092 | 0.346 | 576.0565 | 0.305 | 14 | 21525454 | 21525759 | 0 |  |  |  |
| PREP | IgH3.1039 | A | 0.00194 | 0.07115 | 0.153 | 260.1475 | 3.169 | 6 | 105636367 | 105639536 | 0 |  |  |  |
| AKAP7 | CNVR3066.1 | A | 0.00195 | 0.07138 | 0.293 | 201.354 | 20.974 | 6 | 131438914 | 131459888 | 0 |  | JPT | |
| UNC13C | IgH3.2142 | D | 0.00196 | 0.07161 | -0.339 | 769.0595 | 0.095 | 15 | 51631233 | 51631328 | 1 | 0 |  |  |
| YWHAB | Variation_50664 | D | 0.00196 | 0.07161 | 0.466 | 807.8395 | 4.449 | 20 | 42153551 | 42158000 | 0 |  |  |  |
| ITGA11 | IgH3.2152 | D | 0.00197 | 0.07183 | 0.341 | 671.963 | 0.087 | 15 | 67118327 | 67118414 | 1 | 0 |  |  |
| C3orf59 | IgH3.561 | D | 0.00197 | 0.07183 | -0.357 | 227.2195 | 0.033 | 3 | 194285208 | 194285241 | 0 |  |  |  |
| COPB2 | IgH3.505 | A | 0.00198 | 0.07206 | 0.409 | 438.233 | 0.368 | 3 | 141013584 | 141013952 | 1 | 0 |  |  |
| RAPGEF1 | IgH3.1580 | D | 0.00198 | 0.07206 | -0.416 | 490.249 | 1.229 | 9 | 133032727 | 133033956 | 1 | 0 |  |  |
| TTC21A | IgH3.428 | A | 0.00199 | 0.07228 | -0.408 | 915.8985 | 0.708 | 3 | 38224232 | 38224940 | 1 | 1 |  |  |
| ZBTB4 | CNVR6975.1 | D | 0.00199 | 0.07228 | -0.433 | 108.5665 | 1.037 | 17 | 7205490 | 7206527 | 0 |  |  |  |
| OLFM2 | CNVR7533.1 | A | 0.00202 | 0.07296 | -0.273 | 205.324 | 2.239 | 19 | 10073175 | 10075414 | 0 |  |  |  |
| PRODH | IgH3.2570 | D | 0.00202 | 0.07296 | 0.404 | 307.781 | 288.31 | 22 | 17128428 | 17416739 | 0 |  |  |  |
| SELL | IgH3.117 | A | 0.00203 | 0.07318 | 0.267 | 713.235 | 11.932 | 1 | 168656149 | 168668081 | 0 |  |  |  |
| RASSF3 | Variation_43862 | D | 0.00203 | 0.07318 | -0.446 | 29.8165 | 1.053 | 12 | 63303719 | 63304772 | 0 |  |  |  |
| FAM27L | IgH3.2302 | D | 0.00203 | 0.07318 | -0.235 | 451.9725 | 0 | 17 | 21298088 | 21298088 | 0 |  |  |  |
| DFNA5 | IgH3.1147 | A | 0.00204 | 0.0734 | 0.25 | 791.443 | 0.03 | 7 | 25525511 | 25525541 | 1 |  | CHB | |
| PSMA6 | IgH3.2045 | A | 0.00205 | 0.07362 | -0.368 | 710.628 | 0.451 | 14 | 35554731 | 35555182 | 1 | 0 |  |  |
| MRPS22 | IgH3.505 | A | 0.00207 | 0.07406 | 0.408 | 461.3365 | 0.368 | 3 | 141013584 | 141013952 | 1 | 0 |  | YRI |
| CRB3 | IgH3.2420 | D | 0.00207 | 0.07406 | 0.224 | 829.3845 | 3.388 | 19 | 5589054 | 5592442 | 1 | 0 |  |  |
| TRIM69 | IgH3.2135 | D | 0.00208 | 0.07427 | 0.331 | 935.4025 | 0.465 | 15 | 43767219 | 43767684 | 1 | 0 |  |  |
| TRIM21 | Variation_48809 | D | 0.00208 | 0.07427 | -0.235 | 277.5975 | 3.237 | 11 | 4646318 | 4649555 | 0 |  |  |  |
| DFNA5 | IgH3.1145 | A | 0.00208 | 0.07427 | 0.273 | 457.04 | 0.914 | 7 | 24277470 | 24278384 | 0 |  | CHB | |
| CHID1 | IgH3.1714 | D | 0.00208 | 0.07427 | 0.235 | 718.784 | 8.783 | 11 | 1602542 | 1611325 | 0 |  |  |  |
| NANP | Variation_35913 | A | 0.00208 | 0.07427 | 0.332 | 81.327 | 9.762 | 20 | 25470665 | 25480427 | 0 |  |  |  |
| FAM174B | IgH3.2182 | D | 0.00208 | 0.07427 | -0.355 | 978.023 | 0.725 | 15 | 91959244 | 91959969 | 0 |  |  |  |
| CCDC99 | IgH3.942 | A | 0.00209 | 0.07449 | -0.335 | 856.7925 | 0.655 | 5 | 168097322 | 168097977 | 1 | 0 |  |  |
| CCL3L3 | IgH3.2307 | D | 0.00209 | 0.07449 | 0.241 | 357.5385 | 472.07 | 17 | 31425819 | 31897893 | 0 |  |  |  |
| ZNF543 | CNVR7747.1 | A | 0.00209 | 0.07449 | -0.393 | 1.6035 | 0.739 | 19 | 62527588 | 62528327 | 0 |  |  |  |
| RCSD1 | Variation_38150 | A | 0.0021 | 0.07471 | -0.531 | 450.4865 | 2.299 | 1 | 166355739 | 166358038 | 0 |  |  |  |
| DENND5B | IgH3.1857 | A | 0.0021 | 0.07471 | 0.345 | 450.5625 | 145.41 | 12 | 31152965 | 31298378 | 0 |  |  |  |
| RTEL1 | CNVR7929.1 | D | 0.0021 | 0.07471 | -0.29 | 173.6405 | 1.168 | 20 | 61606009 | 61607177 | 0 |  |  |  |
| GALK2 | CNVR6384.1 | D | 0.00211 | 0.07492 | -0.348 | 1.017 | 3.095 | 15 | 47332068 | 47335163 | 0 |  |  |  |
| PSMD4 | IgH3.95 | A | 0.00212 | 0.07514 | 0.539 | 81.5325 | 4.257 | 1 | 149583860 | 149588117 | 0 |  |  |  |
| MTX2 | Variation_50336 | D | 0.00212 | 0.07514 | 0.436 | 964.6425 | 2.904 | 2 | 175913492 | 175916396 | 0 |  |  |  |
| IFT57 | IgH3.482 | A | 0.00212 | 0.07514 | -0.29 | 211.0515 | 2.852 | 3 | 109183602 | 109186454 | 0 |  |  |  |
| ISCA2 | Variation_49412 | D | 0.00212 | 0.07514 | 0.362 | 705.994 | 8.194 | 14 | 74741204 | 74749398 | 0 |  |  |  |
| RALGDS | IgH3.1584 | D | 0.00212 | 0.07514 | -0.349 | 658.448 | 0.202 | 9 | 135647217 | 135647419 | 0 |  |  |  |
| SNORD45C | Variation_23155 | A | 0.00214 | 0.07563 | -0.222 | 416.0005 | 8.121 | 1 | 75613443 | 75621564 | 0 |  |  |  |
| DPY19L2P2 | CNVR3517.2 | D | 0.00214 | 0.07563 | 0.249 | 912.865 | 32.188 | 7 | 101758653 | 101790841 | 0 |  |  |  |
| C20orf30 | IgH3.2487 | D | 0.00217 | 0.07636 | -0.249 | 644.0305 | 5.919 | 20 | 4394037 | 4399956 | 0 |  |  |  |
| FHIT | IgH3.438 | D | 0.00218 | 0.0766 | -0.372 | 8.68 | 0.018 | 3 | 60452453 | 60452471 | 1 | 0 |  |  |
| FAM86C | IgH3.1768 | A | 0.00218 | 0.0766 | -0.354 | 27.757 | 2.668 | 11 | 71156643 | 71159311 | 0 |  |  |  |
| TRPV3 | IgH3.2278 | A | 0.00219 | 0.07684 | 0.329 | 909.903 | 0.552 | 17 | 2476010 | 2476562 | 1 | 1 |  |  |
| F5 | IgH3.117 | A | 0.00219 | 0.07684 | 0.391 | 865.079 | 11.932 | 1 | 168656149 | 168668081 | 0 |  |  |  |
| SNORA48 | CNVR6975.1 | D | 0.00219 | 0.07684 | -0.341 | 213.85 | 1.037 | 17 | 7205490 | 7206527 | 0 |  |  |  |
| RAD9A | CNVR5211.1 | A | 0.00222 | 0.07756 | 0.278 | 270.954 | 3.275 | 11 | 67191820 | 67195095 | 0 |  |  | JPT |
| HTR1B | CNVR2975.1 | D | 0.00223 | 0.0778 | -0.237 | 163.329 | 1.515 | 6 | 78066681 | 78068196 | 0 |  |  |  |
| EPM2AIP1 | CNVR1349.1 | D | 0.00223 | 0.0778 | 0.199 | 323.1445 | 7.697 | 3 | 36686784 | 36694481 | 0 |  |  |  |
| FOS | Variation_49412 | A | 0.00223 | 0.0778 | -0.435 | 79.8535 | 8.194 | 14 | 74741204 | 74749398 | 0 |  |  |  |
| ATP13A4 | IgH3.562 | D | 0.00224 | 0.07804 | 0.113 | 329.2735 | 12.944 | 3 | 194356173 | 194369117 | 0 |  |  |  |
| GSTM4 | IgH3.75 | D | 0.00225 | 0.07828 | 0.426 | 2.7885 | 32.552 | 1 | 110022101 | 110054653 | 0 |  | JPT | |
| KLF1 | Variation_50072 | A | 0.00225 | 0.07828 | -0.297 | 437.552 | 3.301 | 19 | 13296829 | 13300130 | 0 |  |  |  |
| GPR63 | IgH3.1030 | A | 0.00226 | 0.07852 | -0.346 | 756.947 | 0.267 | 6 | 98129422 | 98129689 | 1 | 1 | YRI | |
| ZNF500 | Variation_42108 | A | 0.00226 | 0.07852 | -0.295 | 964.0985 | 0.445 | 16 | 5713312 | 5713757 | 0 |  |  |  |
| COTL1 | IgH3.2260 | A | 0.00227 | 0.07875 | 0.388 | 464.111 | 2.23 | 16 | 83648163 | 83650393 | 1 | 1 |  |  |
| ZNF320 | CNVR7705.2 | A | 0.00227 | 0.07875 | 0.371 | 525.035 | 1.465 | 19 | 57554521 | 57555986 | 0 |  |  |  |
| LASS4 | IgH3.2426 | D | 0.00228 | 0.07899 | -0.359 | 907.4705 | 0.225 | 19 | 9114342 | 9114567 | 1 | 1 |  |  |
| GPR157 | IgH3.8 | D | 0.00228 | 0.07899 | -0.28 | 843.5865 | 0.027 | 1 | 8255866 | 8255893 | 0 |  |  |  |
| BCAS2 | IgH3.80 | D | 0.00228 | 0.07899 | -0.412 | 294.482 | 7.467 | 1 | 114627997 | 114635464 | 0 |  |  |  |
| MYEOV2 | IgH3.391 | A | 0.00229 | 0.07922 | 0.439 | 223.754 | 0.062 | 2 | 240497944 | 240498006 | 1 |  |  |  |
| SNHG5 | Variation_39463 | D | 0.0023 | 0.07946 | -0.37 | 21.1915 | 8.138 | 6 | 86469567 | 86477705 | 0 |  |  |  |
| RNASE9 | IgH3.2026 | A | 0.0023 | 0.07946 | 0.181 | 2772.944 | 1497.1 | 14 | 18072112 | 19569202 | 0 |  |  |  |
| CCDC34 | IgH3.1743 | D | 0.00231 | 0.07969 | -0.421 | 706.826 | 1.255 | 11 | 26622805 | 26624060 | 1 | 2 |  |  |
| CTNND2 | Variation_38586 | A | 0.00233 | 0.08016 | -0.237 | 310.8685 | 2.632 | 5 | 11181478 | 11184110 | 0 |  |  |  |
| C6orf15 | IgH3.978 | D | 0.00234 | 0.08039 | -0.341 | 121.414 | 19.043 | 6 | 31318580 | 31337623 | 0 |  |  |  |
| HES7 | IgH3.2282 | D | 0.00235 | 0.08062 | -0.324 | 221.7835 | 0.186 | 17 | 8188260 | 8188446 | 0 |  |  |  |
| TMEM68 | IgH3.1419 | D | 0.00236 | 0.08085 | -0.361 | 947.2515 | 0.075 | 8 | 57778445 | 57778520 | 1 | 0 |  |  |
| CYHR1 | Variation_41139 | A | 0.00238 | 0.08131 | -0.359 | 373.7455 | 0.811 | 8 | 146027825 | 146028636 | 0 |  |  |  |
| RHCG | CNVR6504.1 | A | 0.00238 | 0.08131 | -0.235 | 21.314 | 44.776 | 15 | 87871925 | 87916701 | 0 |  |  |  |
| ST13 | CNVR8166.1 | D | 0.00238 | 0.08131 | 0.427 | 647.9385 | 7.324 | 22 | 38922313 | 38929637 | 0 |  |  |  |
| CFH | IgH3.156 | A | 0.00239 | 0.08154 | -0.006 | 92.4435 | 226.45 | 1 | 194956227 | 195182681 | 0 |  |  |  |
| QDPR | IgH3.591 | D | 0.0024 | 0.08176 | -0.436 | 590.718 | 1.784 | 4 | 16520210 | 16521994 | 1 | 1 |  |  |
| FLI1 | IgH3.1814 | A | 0.00242 | 0.08222 | 0.419 | 232.797 | 0.773 | 11 | 127895574 | 127896347 | 1 | 1 |  |  |
| KIAA0391 | IgH3.2045 | A | 0.00242 | 0.08222 | -0.389 | 817.232 | 0.451 | 14 | 35554731 | 35555182 | 1 | 0 |  |  |
| PIRT | IgH3.2284 | D | 0.00242 | 0.08222 | -0.217 | 126.8385 | 1.296 | 17 | 10801816 | 10803112 | 1 | 1 |  |  |
| TRPV6 | IgH3.1240 | A | 0.00242 | 0.08222 | -0.067 | 629.5875 | 4.067 | 7 | 141658786 | 141662853 | 0 |  |  |  |
| KIF18A | IgH3.1744 | A | 0.00242 | 0.08222 | 0.213 | 981.6635 | 0.005 | 11 | 29024196 | 29024201 | 0 |  |  |  |
| SPIN1 | Variation_23000 | A | 0.00243 | 0.08244 | -0.278 | 193.2095 | 4.063 | 9 | 90047101 | 90051164 | 0 |  |  |  |
| C1orf112 | IgH3.117 | A | 0.00244 | 0.08267 | 0.435 | 590.17 | 11.932 | 1 | 168656149 | 168668081 | 0 |  |  |  |
| GLRX2 | IgH3.148 | A | 0.00245 | 0.08289 | 0.371 | 607.8185 | 13.516 | 1 | 191951277 | 191964793 | 0 |  |  |  |
| CCR10 | IgH3.2315 | A | 0.00245 | 0.08289 | -0.366 | 242.503 | 3.93 | 17 | 38330626 | 38334556 | 0 |  |  |  |
| TAPBP | IgH3.988 | D | 0.00246 | 0.08312 | -0.34 | 659.971 | 5.269 | 6 | 34045402 | 34050671 | 0 |  | YRI | |
| MICALCL | Variation_43399 | A | 0.00246 | 0.08312 | -0.243 | 522.6585 | 1.95 | 11 | 11779461 | 11781411 | 0 |  |  |  |
| CDK2 | IgH3.1880 | A | 0.00246 | 0.08312 | 0.521 | 376.444 | 6.611 | 12 | 54276690 | 54283301 | 0 |  |  |  |
| PF4V1 | IgH3.676 | A | 0.00247 | 0.08334 | 0.079 | 952.314 | 0.08 | 4 | 73986195 | 73986275 | 0 |  |  |  |
| LIG1 | IgH3.2464 | D | 0.00247 | 0.08334 | 0.18 | 395.4335 | 15.471 | 19 | 53741112 | 53756583 | 0 |  |  |  |
| ELAC2 | Variation_55517 | A | 0.00249 | 0.08379 | 0.377 | 796.01 | 0.556 | 17 | 13645168 | 13645724 | 0 |  |  |  |
| ADORA2A | IgH3.2582 | D | 0.00249 | 0.08379 | 0.302 | 690.9275 | 268.77 | 22 | 23986238 | 24255005 | 0 |  |  |  |
| DNAJC11 | IgH3.6 | A | 0.0025 | 0.08401 | -0.188 | 596.735 | 0.254 | 1 | 7247546 | 7247800 | 1 | 0 |  |  |
| ACTL6A | IgH3.546 | D | 0.00252 | 0.08445 | -0.391 | 408.573 | 4.89 | 3 | 180370016 | 180374906 | 1 | 1 |  |  |
| CTDSPL | IgH3.427 | A | 0.00252 | 0.08445 | -0.394 | 6.778 | 10.216 | 3 | 37951704 | 37961920 | 0 |  |  |  |
| TPD52L1 | IgH3.1048 | A | 0.00253 | 0.08467 | -0.525 | 277.345 | 0.21 | 6 | 125294220 | 125294430 | 1 | 0 |  |  |
| NTNG1 | IgH3.73 | A | 0.00253 | 0.08467 | -0.389 | 879.041 | 3.261 | 1 | 108535746 | 108539007 | 0 |  |  |  |
| NDUFA11 | CNVR7515.1 | D | 0.00253 | 0.08467 | 0.282 | 936.8605 | 33.719 | 19 | 6804075 | 6837794 | 0 |  |  |  |
| KIFC1 | IgH3.988 | D | 0.00255 | 0.08511 | 0.4 | 566.284 | 5.269 | 6 | 34045402 | 34050671 | 0 |  |  |  |
| FAM21C | IgH3.1643 | D | 0.00256 | 0.08533 | 0.372 | 184.3385 | 3.363 | 10 | 45392878 | 45396241 | 0 |  |  |  |
| PPPDE1 | CNVR571.1 | A | 0.00257 | 0.08554 | -0.317 | 144.179 | 138.92 | 1 | 243124604 | 243263523 | 0 |  |  |  |
| NDUFB5 | IgH3.546 | A | 0.00258 | 0.08576 | -0.304 | 447.554 | 4.89 | 3 | 180370016 | 180374906 | 1 | 1 |  |  |
| C4B | Variation_32788 | D | 0.00259 | 0.08598 | -0.285 | 395.3375 | 8.577 | 6 | 32467750 | 32476327 | 0 |  |  |  |
| UCK1 | Variation_57977 | A | 0.00259 | 0.08598 | -0.224 | 1015.914 | 9.416 | 9 | 132381538 | 132390954 | 0 |  |  |  |
| ZFP36 | Variation_30904 | D | 0.00259 | 0.08598 | 0.374 | 623.141 | 4.603 | 19 | 43969766 | 43974369 | 0 |  |  |  |
| SLBP | IgH3.572 | A | 0.0026 | 0.08619 | -0.54 | 713.4785 | 0.317 | 4 | 960756 | 961073 | 0 |  |  |  |
| AVP | Variation_38443 | D | 0.0026 | 0.08619 | -0.26 | 971.6365 | 3.212 | 20 | 3985528 | 3988740 | 0 |  |  |  |
| PHF17 | IgH3.729 | A | 0.00261 | 0.08641 | -0.263 | 396.5215 | 0.242 | 4 | 129586628 | 129586870 | 1 | 1 |  |  |
| SSNA1 | Variation_57995 | A | 0.00261 | 0.08641 | -0.223 | 687.5975 | 3.95 | 9 | 139893331 | 139897281 | 0 |  |  | JPT |
| RERE | IgH3.8 | D | 0.00262 | 0.08662 | -0.433 | 114.8395 | 0.027 | 1 | 8255866 | 8255893 | 0 |  |  |  |
| HIST1H4J | Variation_23078 | A | 0.00263 | 0.08684 | -0.233 | 712.6085 | 6.383 | 6 | 28615859 | 28622242 | 0 |  |  |  |
| PER1 | CNVR6975.1 | D | 0.00263 | 0.08684 | -0.358 | 785.5235 | 1.037 | 17 | 7205490 | 7206527 | 0 |  |  |  |
| PTPN2 | IgH3.2355 | A | 0.00264 | 0.08705 | -0.402 | 56.058 | 0.058 | 18 | 12772761 | 12772819 | 1 | 0 |  |  |
| CTPS | CNVR154.1 | A | 0.00264 | 0.08705 | 0.22 | 526.5925 | 6.514 | 1 | 41764057 | 41770571 | 0 |  |  |  |
| CCDC83 | IgH3.1779 | D | 0.00266 | 0.08749 | -0.214 | 1029.312 | 0.241 | 11 | 84247054 | 84247295 | 0 |  |  |  |
| THAP3 | IgH3.6 | A | 0.0027 | 0.08837 | -0.277 | 634.384 | 0.254 | 1 | 7247546 | 7247800 | 1 | 0 |  |  |
| CALR | IgH3.2432 | D | 0.0027 | 0.08837 | 0.296 | 506.1695 | 0 | 19 | 12407189 | 12407189 | 0 |  |  |  |
| SGSH | IgH3.2339 | D | 0.00271 | 0.08859 | -0.422 | 438.9495 | 4.386 | 17 | 76244376 | 76248762 | 0 |  |  |  |
| DCAKD | IgH3.2319 | A | 0.00272 | 0.08881 | 0.34 | 114.1315 | 1.166 | 17 | 40585082 | 40586248 | 0 |  |  |  |
| NDUFB7 | IgH3.2436 | A | 0.00274 | 0.08924 | 0.311 | 295.125 | 2.181 | 19 | 14837103 | 14839284 | 1 | 1 |  |  |
| STMN1 | IgH3.24 | D | 0.00274 | 0.08924 | 0.386 | 686.099 | 95.086 | 1 | 25455866 | 25550952 | 0 |  |  |  |
| KCNQ1 | CNVR5024.1 | A | 0.00275 | 0.08946 | -0.327 | 229.328 | 1.372 | 11 | 2396214 | 2397586 | 0 |  |  |  |
| BRD4 | IgH3.2436 | A | 0.00278 | 0.09011 | -0.357 | 399.5415 | 2.181 | 19 | 14837103 | 14839284 | 1 | 1 |  |  |
| PLA2G4D | Variation_33284 | D | 0.00281 | 0.09075 | 0.23 | 271.036 | 0.564 | 15 | 39889854 | 39890418 | 0 |  |  |  |
| OPA3 | CNVR7667.1 | D | 0.00282 | 0.09096 | 0.37 | 473.895 | 6.466 | 19 | 50280751 | 50287217 | 0 |  |  |  |
| URB2 | IgH3.181 | A | 0.00284 | 0.09139 | 0.196 | 421.8635 | 0.203 | 1 | 228267551 | 228267754 | 1 | 0 |  |  |
| PIK3CG | IgH3.1212 | D | 0.00284 | 0.09139 | -0.326 | 937.408 | 0.027 | 7 | 107251415 | 107251442 | 0 |  |  |  |
| C14orf139 | IgH3.2098 | D | 0.00285 | 0.0916 | 0.43 | 835.8535 | 0.498 | 14 | 95780871 | 95781369 | 1 | 0 |  |  |
| EVX2 | IgH3.343 | D | 0.00285 | 0.0916 | 0.32 | 324.2425 | 1.011 | 2 | 176979756 | 176980767 | 0 |  |  |  |
| SNX10 | IgH3.1147 | A | 0.00285 | 0.0916 | 0.35 | 813.7605 | 0.03 | 7 | 25525511 | 25525541 | 1 |  |  |  |
| CD109 | IgH3.1014 | D | 0.00286 | 0.09181 | -0.345 | 118.234 | 4.884 | 6 | 74649173 | 74654057 | 0 |  |  |  |
| C14orf21 | CNVR6096.1 | D | 0.00286 | 0.09181 | 0.414 | 986.021 | 0.939 | 14 | 24828066 | 24829005 | 0 |  |  |  |
| UNC13C | IgH3.2144 | D | 0.00288 | 0.09223 | -0.452 | 201.042 | 0.004 | 15 | 52601289 | 52601293 | 1 |  |  |  |
| IL1RAP | CNVR1674.1 | A | 0.00289 | 0.09244 | -0.178 | 720.0695 | 2.411 | 3 | 192493924 | 192496335 | 0 |  |  |  |
| EGFL8 | IgH3.982 | D | 0.00289 | 0.09244 | 0.358 | 81.6665 | 26.427 | 6 | 32173758 | 32200185 | 0 |  |  |  |
| MED28 | IgH3.591 | D | 0.0029 | 0.09265 | -0.276 | 710.996 | 1.784 | 4 | 16520210 | 16521994 | 1 | 1 |  |  |
| ST5 | Variation_38243 | A | 0.0029 | 0.09265 | -0.384 | 658.5645 | 6.189 | 11 | 9391796 | 9397985 | 0 |  |  |  |
| BRD4 | IgH3.2439 | D | 0.0029 | 0.09265 | -0.382 | 64.5085 | 0.125 | 19 | 15171108 | 15171233 | 0 |  |  |  |
| MLC1 | IgH3.2604 | A | 0.0029 | 0.09265 | -0.095 | 979.9575 | 0.301 | 22 | 47873120 | 47873421 | 0 |  |  |  |
| PTPRM | CNVR7244.1 | D | 0.00291 | 0.09285 | 0.236 | 1115.566 | 0.481 | 18 | 6861761 | 6862242 | 0 |  |  |  |
| GSTT1 | CNVR8108.1 | A | 0.00294 | 0.09348 | -0.143 | 610.039 | 2.662 | 22 | 22101503 | 22104165 | 0 |  | CEU|CHB|JPT|YRI | |
| HLA-DRB5 | IgH3.984 | D | 0.00295 | 0.09368 | -0.313 | 103.085 | 6.987 | 6 | 32499966 | 32506953 | 0 |  | YRI | CHB|JPT|YRI |
| TMEM147 | CNVR7629.1 | A | 0.00295 | 0.09368 | -0.253 | 697.2725 | 45.309 | 19 | 41449253 | 41494562 | 0 |  |  |  |
| ACOT8 | IgH3.2510 | A | 0.00296 | 0.09389 | -0.417 | 604.5145 | 0.268 | 20 | 43307224 | 43307492 | 1 | 0 |  |  |
| PURA | Variation_51580 | D | 0.00296 | 0.09389 | 0.407 | 980.1495 | 3.461 | 5 | 140457078 | 140460539 | 0 |  |  |  |
| RASSF2 | Variation_44048 | A | 0.00296 | 0.09389 | -0.245 | 959.1755 | 4.056 | 20 | 3769071 | 3773127 | 0 |  |  |  |
| MYH2 | IgH3.2284 | D | 0.00297 | 0.09409 | -0.201 | 421.741 | 1.296 | 17 | 10801816 | 10803112 | 1 | 1 |  |  |
| DPH3 | IgH3.411 | D | 0.00298 | 0.0943 | 0.358 | 788.586 | 0.234 | 3 | 15489067 | 15489301 | 1 | 1 |  |  |
| SYNGAP1 | IgH3.984 | D | 0.003 | 0.09471 | -0.363 | 1016.162 | 6.987 | 6 | 32499966 | 32506953 | 0 |  |  |  |
| TYRO3 | Variation_40086 | A | 0.003 | 0.09471 | -0.178 | 1.985 | 0.679 | 15 | 39647025 | 39647704 | 0 |  |  |  |
| NFATC1 | IgH3.2413 | D | 0.00301 | 0.09491 | -0.344 | 647.498 | 13.722 | 18 | 75977894 | 75991616 | 1 | 1 |  |  |
| SNX24 | CNVR2592.1 | A | 0.00301 | 0.09491 | -0.295 | 868.8855 | 5.799 | 5 | 123162714 | 123168513 | 0 |  |  |  |
| WISP1 | CNVR4057.1 | D | 0.00302 | 0.09511 | -0.288 | 105.639 | 2.154 | 8 | 134397580 | 134399734 | 0 |  |  |  |
| BOP1 | CNVR4125.1 | A | 0.00302 | 0.09511 | 0.348 | 182.649 | 6.121 | 8 | 145657105 | 145663226 | 0 |  |  |  |
| BRD7 | CNVR6723.1 | A | 0.00302 | 0.09511 | -0.169 | 653.31 | 0.471 | 16 | 49588931 | 49589402 | 0 |  |  |  |
| ICOSLG | CNVR8035.1 | A | 0.00303 | 0.09532 | -0.14 | 331.4475 | 7.818 | 21 | 44150667 | 44158485 | 0 |  |  |  |
| SLC7A6 | IgH3.2234 | A | 0.00304 | 0.09552 | 0.17 | 308.322 | 2.356 | 16 | 67184073 | 67186429 | 1 | 0 |  |  |
| RNF32 | Variation_52170 | D | 0.00304 | 0.09552 | -0.349 | 662.252 | 5.776 | 7 | 156809511 | 156815287 | 0 |  |  |  |
| POLR3K | Variation_49688 | D | 0.00305 | 0.09572 | -0.35 | 258.2995 | 2.685 | 16 | 299954 | 302639 | 0 |  |  |  |
| POLR1B | CNVR933.1 | D | 0.00306 | 0.09592 | 0.367 | 800.7195 | 99.653 | 2 | 113884126 | 113983779 | 0 |  |  |  |
| TMCO1 | IgH3.114 | A | 0.00307 | 0.09612 | -0.341 | 905.013 | 0.1 | 1 | 164887518 | 164887618 | 0 |  |  |  |
| EFHA2 | IgH3.1392 | A | 0.00307 | 0.09612 | 0.029 | 13.589 | 0.532 | 8 | 16963495 | 16964027 | 0 |  |  |  |
| ITPR1 | IgH3.400 | D | 0.00308 | 0.09632 | 0.265 | 1074.232 | 1.476 | 3 | 3613783 | 3615259 | 0 |  |  |  |
| MEX3A | IgH3.104 | A | 0.0031 | 0.09672 | -0.302 | 900.717 | 22.842 | 1 | 155225558 | 155248400 | 1 | 1 |  |  |
| NMT1 | IgH3.2319 | A | 0.0031 | 0.09672 | 0.269 | 66.4425 | 1.166 | 17 | 40585082 | 40586248 | 0 |  |  | JPT |
| PPP1R15A | Variation_35661 | A | 0.0031 | 0.09672 | -0.369 | 571.6145 | 12.864 | 19 | 54647342 | 54660206 | 0 |  |  |  |
| ZNF517 | CNVR4130.1 | A | 0.00311 | 0.09692 | -0.347 | 205.4325 | 3.214 | 8 | 145796373 | 145799587 | 0 |  |  |  |
| ZNF511 | CNVR4985.1 | A | 0.00313 | 0.09731 | 0.366 | 137.462 | 1.78 | 10 | 134837962 | 134839742 | 0 |  |  |  |
| MRPL10 | CNVR7121.1 | A | 0.00313 | 0.09731 | -0.284 | 801.5345 | 51.609 | 17 | 44087108 | 44138717 | 0 |  |  |  |
| FAM158A | Variation_35245 | A | 0.00314 | 0.09751 | 0.153 | 37.333 | 12.77 | 14 | 23723043 | 23735813 | 0 |  |  |  |
| SNAPC1 | IgH3.2067 | D | 0.00314 | 0.09751 | 0.41 | 332.9995 | 2.475 | 14 | 61650100 | 61652575 | 0 |  |  |  |
| BHLHE41 | Variation_49036 | A | 0.00315 | 0.09771 | -0.334 | 627.667 | 3.085 | 12 | 25540624 | 25543709 | 0 |  |  |  |
| HMP19 | CNVR2688.1 | A | 0.00316 | 0.09791 | -0.249 | 929.269 | 4.559 | 5 | 174368607 | 174373166 | 0 |  |  |  |
| SUDS3 | IgH3.1924 | A | 0.00316 | 0.09791 | 0.412 | 423.075 | 7.297 | 12 | 117746205 | 117753502 | 0 |  |  |  |
| SUV420H1 | CNVR5211.1 | A | 0.00316 | 0.09791 | -0.401 | 518.5385 | 3.275 | 11 | 67191820 | 67195095 | 0 |  |  |  |
| PIP | IgH3.1249 | A | 0.00317 | 0.0981 | -0.508 | 797.626 | 0.531 | 7 | 143341017 | 143341548 | 0 |  |  |  |
| DNAJC15 | Variation_49211 | D | 0.00317 | 0.0981 | -0.389 | 882.478 | 2.795 | 13 | 41657253 | 41660048 | 0 |  |  |  |
| ME3 | IgH3.1782 | A | 0.00317 | 0.0981 | -0.32 | 37.9165 | 0.225 | 11 | 85983465 | 85983690 | 0 |  |  |  |
| C15orf32 | IgH3.2178 | D | 0.00317 | 0.0981 | -0.349 | 1012.19 | 0.364 | 15 | 89818623 | 89818987 | 0 |  |  |  |
| C6orf167 | IgH3.1031 | A | 0.00318 | 0.0983 | 0.313 | 671.15 | 2.33 | 6 | 98439580 | 98441910 | 1 | 0 |  |  |
| DUOXA1 | CNVR6378.1 | D | 0.00319 | 0.09849 | -0.139 | 819.134 | 0.556 | 15 | 44022575 | 44023131 | 0 |  |  |  |
| MPDU1 | CNVR6975.1 | D | 0.0032 | 0.09869 | 0.438 | 224.997 | 1.037 | 17 | 7205490 | 7206527 | 0 |  |  |  |
| ERI1 | Variation_52642 | A | 0.00321 | 0.09888 | 0.34 | 438.6915 | 1.023 | 8 | 9352194 | 9353217 | 0 |  |  |  |
| FDXR | Variation_49925 | A | 0.00321 | 0.09888 | -0.216 | 33.9355 | 14.121 | 17 | 70348607 | 70362728 | 0 |  |  |  |
| RNASE3 | IgH3.2026 | D | 0.00322 | 0.09907 | 0.31 | 3106.307 | 1497.1 | 14 | 18072112 | 19569202 | 0 |  |  |  |
| RPA3 | IgH3.1119 | A | 0.00322 | 0.09907 | 0.45 | 91.246 | 0.98 | 7 | 7775667 | 7776647 | 0 |  |  |  |
| PRKG1 | IgH3.1648 | D | 0.00322 | 0.09907 | 0.236 | 405.327 | 3.444 | 10 | 52669510 | 52672954 | 0 |  |  |  |
| BIRC8 | IgH3.2471 | D | 0.00324 | 0.09946 | 0.315 | 291.852 | 33.47 | 19 | 58210559 | 58244029 | 0 |  |  |  |
| NOL9 | Variation_54304 | A | 0.00326 | 0.09984 | -0.395 | 970.805 | 1.952 | 1 | 7492401 | 7494353 | 0 |  |  |  |
| DNAJB6 | IgH3.1278 | A | 0.00326 | 0.09984 | 0.355 | 957.2025 | 0.968 | 7 | 157820369 | 157821337 | 0 |  |  |  |

**Supporting Methods**

***Permutation and Binomial Test Comparison:***

Following is the table comparing the p-values using the Binomial test and the Permutation test (described in the main text) for detecting CNVs using IgC2N. Table S3 presents the average correlation between p-values of permutation and binomial tests described in the Methods section of the main text.

**Table S8: Comparison of P-values Between Permutation and Binomial Tests**

| **Qauntiles** | **N=200** | **N=400** |
| --- | --- | --- |
| 0% | 0.999994 | 0.999994 |
| 25% | 0.999997 | 0.999997 |
| 50% | 0.999998 | 0.999998 |
| 75% | 0.999999 | 0.999999 |
| 100% | 1 | 1 |

***Genotyping Errors:***

0-1 Genotypes and 3-4 genotypes:

There are two types of probable genotyping errors that can occur. The first is called the 3-4 genotyping error. This mostly occurs in segmental duplication or high marker density areas where the relative distance between copy number classes (1 CN Class difference, refer to the main text) is smaller from the “population” of differences (the 1 CN class difference for all CNVs). Hence the IgC2N algorithm renders these as CN classes 3 and 4 (example provided in **Figure S9** left panel). We should note that these still may be 3-4 CN genotypes. The other genotyping error makes an accurate CN genotype assignment is difficult. An example is shown in the right panel of **Figure S9**, where there are two classes with high frequency in each of them and the zero is roughly at the junction between the two classes. One could hypothesize both ways that a frequent deletion (amplification) class could result in the reference model having deletions (amplifications) thereby inducing a positive (negative) location bias. Since a 1-2 CN genotype class is not feasible owing to the existence of the “0” class based on the “zero-presence” hypothesis described in the text, IgC2N calls it 0-1.

A breakdown of the genotyping errors is given with respect to the novel CNVs and the non-novel CNVs.

**Table S9: Genotyping Errors**

|  | **Novel CNVs** | **Known CNVs** |  |
| --- | --- | --- | --- |
| **0-1** | 62 | 104 |  |
| **3-4** | 3 | 21 |  |
|  | | |  |

**Figure S9: Examples of 0-1 and 3-4 genotyping errors.** Left panel shows an example of 3-4 genotyping error while the right panel shows that of 0-1 genotyping error.
